# Association between gut microbiota and menstrual disorders: a two-sample Mendelian randomization study

**DOI:** 10.3389/fmicb.2024.1321268

**Published:** 2024-03-07

**Authors:** Yufan Yao, Haoran Hu, Longhao Chen, Hong Zheng

**Affiliations:** ^1^College of Basic Medical Science, Zhejiang Chinese Medical University, Hangzhou, China; ^2^Hangzhou TCM Hospital of Zhejiang Chinese Medical University (Hangzhou Hospital of Traditional Chinese Medicine), Hangzhou, China; ^3^The Third School of Clinical Medicine, Zhejiang Chinese Medical University, Hangzhou, China

**Keywords:** genetics, gut microbiota, menstrual disorder, Mendelian randomization, SNPs

## Abstract

**Background:**

Evidence from observational studies and clinical trials suggests that the gut microbiota is associated with gynecological diseases. However, the causal relationship between gut microbiota and menstrual disorders remains to be determined.

**Methods:**

We obtained summary data of gut microbiota from the global consortium MiBio-Gen’s genome-wide association study (GWAS) dataset and data on menstrual disorders from the IEU Open GWAS project. MR-Egger, weighted median, inverse variance weighted, simple mode, and weighted mode were used to examine the causal association between gut microbiota and menstrual disorders. Thorough sensitivity studies were performed to confirm the data’s horizontal pleiotropy, heterogeneity, and robustness.

**Results:**

Through MR analysis of 119 kinds of gut microbiota and 4 kinds of clinical phenotypes, it was discovered that 23 different kinds of gut microbiota were loosely connected to menstrual disorders. After FDR correction, the results showed that only Escherichia/Shigella (*p* = 0.00032, *P*_FDR_ = 0.0382, OR = 1.004, 95%CI = 1.002–1.006) is related to menstrual disorders.

**Conclusion:**

According to our MR Analysis, there are indications of a causal relationship between menstrual disorders and gut microbiota. This finding could lead to new discoveries into the mechanisms behind menstrual disorders and clinical research involving the microbiota.

## 1 Introduction

Menstrual disorders are a common gynecological condition. Irregular menstrual cycles, long menstrual cycles, and increased or decreased menstrual blood volume are the main related manifestations (Chen W. et al., 2023). It causes considerable worry for women.

During the menstrual cycle, hormone and physiological changes can affect the richness and diversity of the urinary and reproductive microbiota (Holdcroft et al., 2023; Cao et al., 2024). Song et al. investigated vaginal microbiota changes at various periods of the menstrual cycle and discovered increased alpha diversity with decreased relative abundance of *Lactobacillus* spp. and a greater percentage of other bacteria, including *Peptostreptococcus*, *Anaerococcus*, and *Streptococcus* species (Chen et al., 2017; Song et al., 2020). Research by Krog et al. demonstrated that during menstruation, women’s vaginal microbiomes are more diverse. Furthermore, they discovered a rise in the non-resident bacteria that cause bacterial vaginosis; however, these bacteria decreased in the luteal and follicular phases (Krog et al., 2022; Grobeisen-Duque et al., 2023). According to a recent study, multiple gut microbiota functional pathways are significantly correlated with the severity score of dysmenorrhea symptoms (Chen C. et al., 2023). Women with more severe symptoms of dysmenorrhea had higher percentages of possibly pro-inflammatory bacteria in their vaginal microbiograms and lower percentages of *Lactobacillus* (Nabeh, 2023).

Some research has also been carried out on the effect of microbiota on menstruation. One study reported that deficiencies in the salivary and fecal microbiota led to significant changes in menstruation and that the diversity of the vaginal microbiota increased during menstruation due to the expansion of *Lactobacillus* during the follicular and luteal phases (Krog et al., 2022). Furthermore, microbial dysbiosis itself can result in elevated insulin levels and a condition known as insulin resistance because it triggers the immune system and stimulates the release of several pro-inflammatory cytokines. Menstrual issues result from this as it raises androgen production and throws off the natural balance between estrogen and progesterone (Tremellen and Pearce, 2012; Nabeh, 2023). Thus, menstruation and microorganisms are inextricably linked.

In the gastrointestinal system, the gut microbiota plays an important role in digestion regulation, but its importance goes much beyond that. The structure of the gut microbiota affects the onset and progression of metabolic and endocrine diseases (Mukherjee et al., 2023). Randomized controlled trials of the gut microbiota, as opposed to observational studies, may aid in proving causation. Unfortunately, due to objective elements such as technology, study methodology, and other confounding factors including age, environment, eating habits, and lifestyle, screening for strains still has substantial limits in early diagnosis and prognosis. It might be challenging to effectively control for these factors in observational studies (Sher et al., 2021). There is growing evidence that the human gut microbiota plays a role in gynecologic diseases (Flores et al., 2012; Lindheim et al., 2017; Parida and Sharma, 2019; Zhao et al., 2020; Zhou et al., 2020; Cao et al., 2024; Huang et al., 2024). If changes in the composition and/or function of the microbiota can be demonstrated to have clinically advantageous effects, then using the gut microbiota’s functioning as an alternative to pharmaceutical intervention is possible.

Several studies have recently been conducted to study the association between the hormonal variations associated with the menstrual cycle and the gut microbiota. Reports claim that estrogen affects the gut microbiota in all parts of the body (Vieira et al., 2017). When women have sufficient estrogen in their bodies, their intestinal microbiota exhibits species diversity, with beneficial bacteria dominating and the growth of harmful bacteria being inhibited. Due to the presence of sex hormone receptors in the digestive tract, many healthy women suffer changes in gastrointestinal symptoms during the menstrual cycle (Bharadwaj et al., 2015; Mohib et al., 2018). For instance, early menstruation is characterized by lower stool consistency than mid-menstruation; visceral somatic impulses may be perceived more strongly, resulting in pain, bloating, and nausea, particularly on the first day of the menstrual cycle. In addition, hormonal changes during menstruation can lead to alterations in the function and activity of the body’s microbiota. This is due to the microbiota’s control over steroid hormone levels, including estrogen (Parida and Sharma, 2019), microbiota can metabolize sex hormones through numerous enzymes, such as hydroxysteroid dehydrogenase, which controls the balance of active and inactive steroids.

An analytical technique called Mendelian randomization (MR) is utilized to determine the causal connection between the observed relationships between modifiable exposures or risk factors and clinically relevant outcomes. To correlate with SNP outcome connections and merge them into a single causal estimate, two-sample MR analysis can utilize SNPs (exposure) from independent genome-wide association studies (GWASs). As the number of GWASs linking illnesses and the gut microbiota has risen rapidly (MiBioGen Consortium Initiative et al., 2018; Kurilshikov et al., 2021), large-scale pooled measurements have become more widespread, allowing two-sample MR analysis to have significantly enhanced statistical power (Kurilshikov et al., 2021). MR allows us to understand whether there is a causal relationship between intestinal flora and menstrual disorders, which can inform clinical and research studies.

GWASs have revolutionized the study of complex disease genetics by analyzing millions of genetic variants present in the genomes of several individuals to determine the connections between genotype and phenotype over the past decade (Visscher et al., 2017). A GWAS provides an agnostic method for studying the genetic basis of complex diseases. The GWAS directory has 427,870 associations and 6,041 articles as of October 2022.

## 2 Methods

### 2.1 Study design

This study is reported following the Strengthening the Reporting of Observational Studies in Epidemiology Using Mendelian Randomization guidelines (STROBE-MR, S1 Checklist) (Skrivankova et al., 2021a,b).

**Figure 1:**
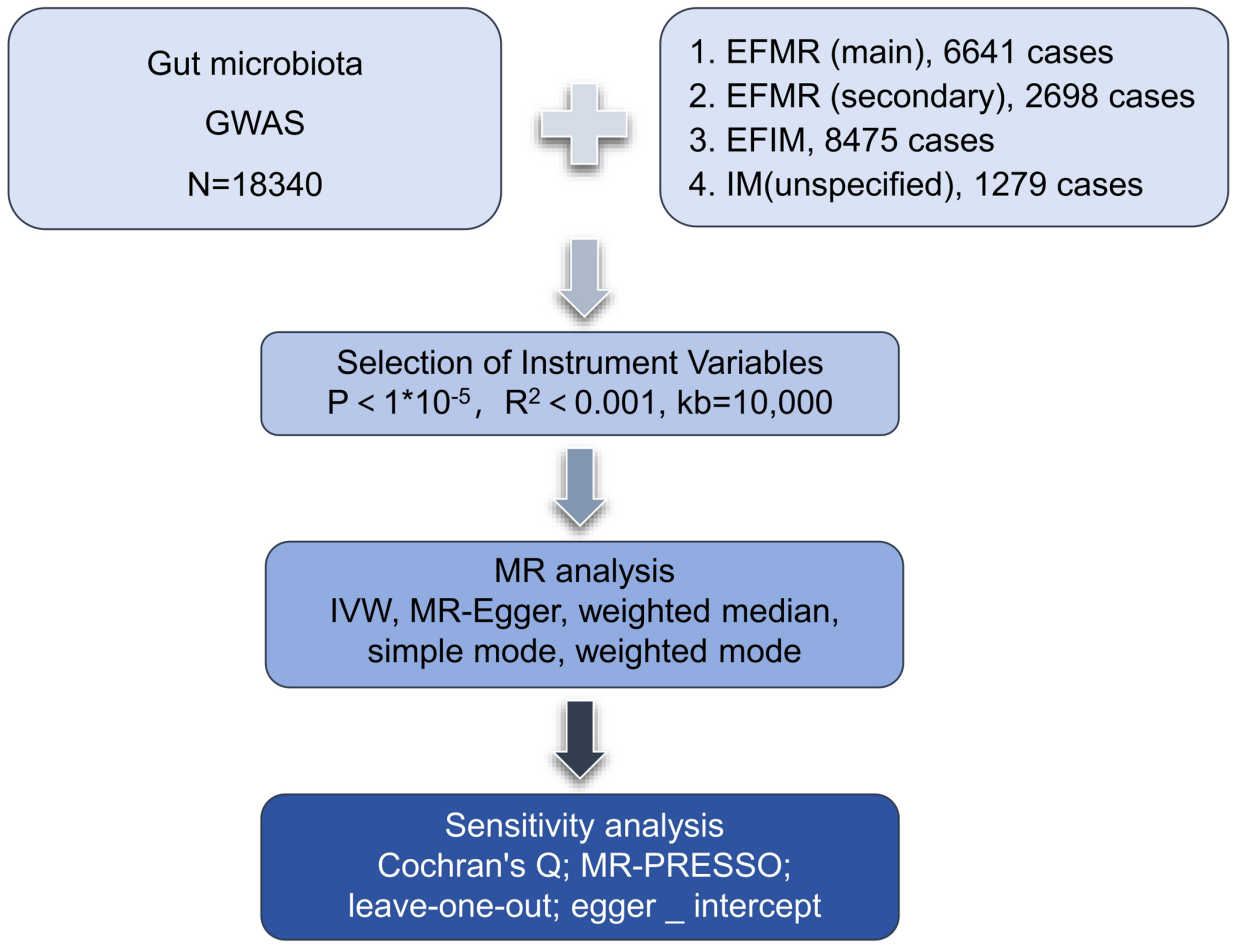
The flowchart of the study.

**Figure 2:**
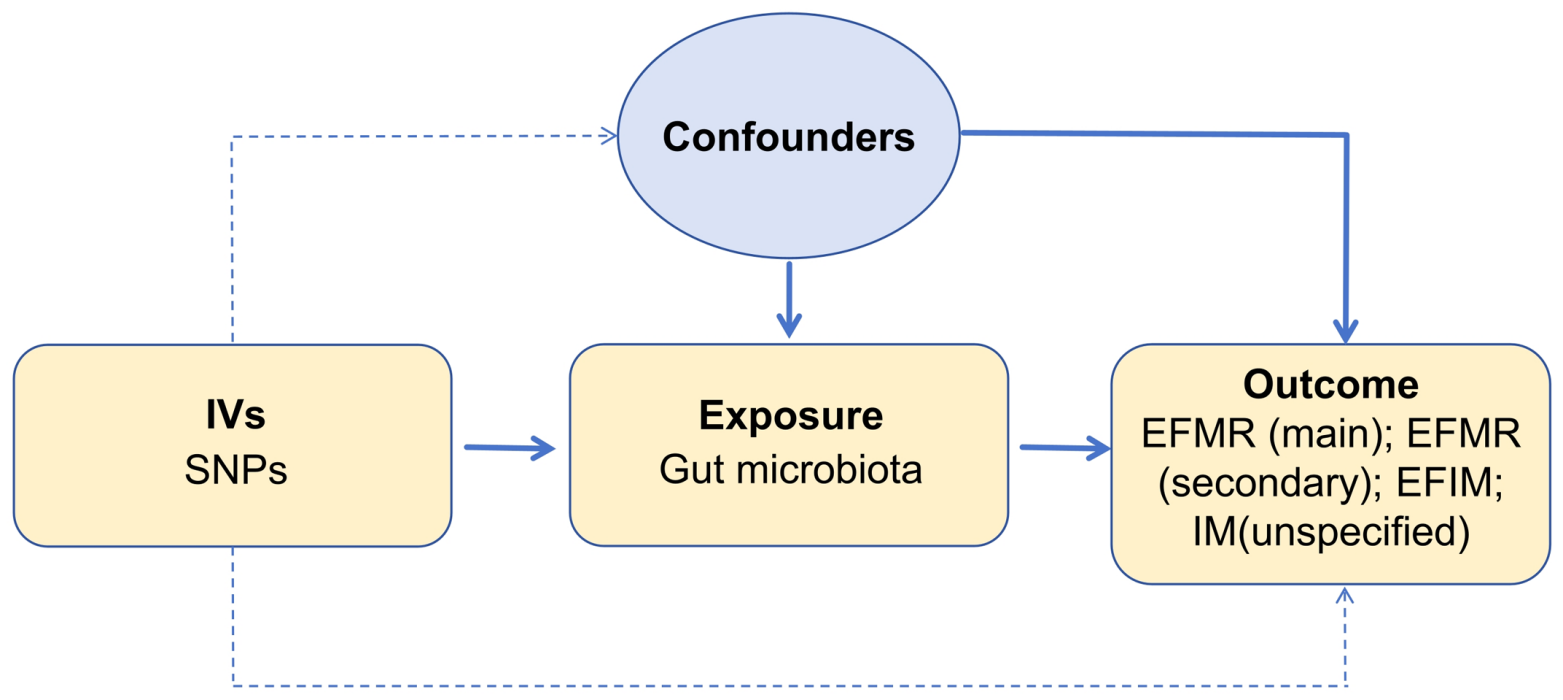
Three assumptions of MR analysis.

We assessed the causal relationship between 119 microbial communities and 4 clinical phenotypes based on a two-sample MR analysis. The research process is shown in Figure 1. MR uses genetic variation to represent risk factors, and therefore, valid instrumental variables (IVs) in causal inference must satisfy three key assumptions: (1) Exposure has a direct correlation with genetic variation; (2) There is no correlation between genetic variation and potential variables between exposure and result; (3) Other than exposure, genetic variation has no effect on outcome through other mechanisms (Bowden and Holmes, 2019) (Figure 2).

### 2.2 Exposure data

SNPs from the global consortium MiBio-Gen’s GWAS dataset that are associated with the composition of the human gut microbiome were used as instrumental variables (IVs). In order to investigate the relationship between autosomal human genetic variants and the gut microbiome, this multi-ethnic large-scale GWAS brought together genotyping and 16 s ribosomal RNA gene sequencing data from 18,340 participants. A total of 211 taxa (131 genera, 35 families, 20 orders, 16 classes, and 9 phyla) were included (Long et al., 2023). In our study, we kept only data of genus.

### 2.3 Outcome data

Summary statistics for menstruation were obtained from a GWAS meta-analysis.^1^ The genetic association data consisted: (1) The diagnostic criteria of diagnoses-main ICD10: N92.0 Excessive and frequent menstruation with regular cycle (EFMR (main)) containing 463,010 participants (*N* = 6,641 cases, 456,369 controls) with a total of 9,851,867 SNPs; (2) The diagnostic criteria of diagnoses-secondary ICD10: N92.0 Excessive and frequent menstruation with regular cycle (EFMR (secondary)) containing 463,010 participants (*N* = 2,698 cases, 460,312 controls) with a total of 9,851,867 SNPs; (3) The diagnostic criteria of Diagnoses-main ICD10: N92.6 Irregular menstruation, unspecified (IM (unspecified)) containing 463,010 participants (*N* = 1,279 cases, 461,731controls) with a total of 9,851,867 SNPs; (4) The diagnostic criteria of Diagnoses-main ICD10: N92 Excessive, frequent and irregular menstruation (EFIM) containing 361,194 participants (*N* = 8,475 cases, 352,719 controls) with a total of 12,983,417 SNPs.

All cases and controls were Europeans. The diagnostic criteria for these four packets are based on ICD-10 criteria. Ethical endorsement was not sought for the study as it only used publicly available GWAS summary statistics and did not attempt to identify individual-level data. Because this is publicly published data by GWAS, other infections and disorders/issues have been ruled out.

### 2.4 Instrumental variable selection

The gut microbiota served as the exposure, while menstruation serves as a result. First, we set up the parameters for identifying IVs with a genome-wide significance of *p* < 1 × 10^−5^ and a chained unbalanced clustering algorithm with an R2 threshold of 0.001 over a 10,000 kb area to assure independence of IV exposure (R4.3.1 software; Package: TwoSampleMR; VariantAnnotation; gwasglue). Furthermore, we regarded IVs with F-statistics >10 as powerful tools and saved them for the analyses that followed in order to prevent the bias caused by weak instruments (R4.3.1 software; Package: ieugwasr).

### 2.5 MR analysis

In order to evaluate the causal link between exposure factors and outcome, we utilized MR-Egger, weighted median, inverse variance weighted (IVW), simple mode, and weighted mode (R4.3.1 software, package: TwoSampleMR; VariantAnnotation; gwasglue). The benefit of IVW is that it makes it possible to measure the situation objectively and without experiencing horizontal pleiotropy. Therefore, the results of multiple IVs are mainly based on the IVW method, supplemented by four other methods (Bowden et al., 2015). To ensure that each IV was associated with the same effector allele, we excluded palindromes and incompatible SNPs when harmonizing exposure and outcome statistics, and excluded SNPs linked to exposures that the GWAS outcome statistics were unable to match.

Several sensitivity studies were conducted to evaluate the results’ robustness (R4.3.1 software, package: TwoSampleMR). MR-PRESSO was used to detect polybiotic effect bias and correct for polybiotic effects by rejecting outliers, and it has the ability to both detect and correct pleiotropy in MR analysis, and get a causal effect estimate (Bowden et al., 2016) and examine whether the results are driven by the directional horizontal pleiotropy (Burgess and Thompson, 2017). To ascertain whether a single SNP was responsible for the causal signal, leave-one-out analyses were carried out. The identified causal relationship can be regarded as directionally reasonable if the IVs account for more of the exposure difference than the outcome difference. We used Cochran’s Q statistics and a two-sample MR package between instruments testing for heterogeneity. Evidence of heterogeneity and invalid instruments can be found when Q exceeds the number of instruments minus one, or a significant Q statistic at a *p*-value < 0.05 can mean the presence of heterogeneity (Greco et al., 2015; Bowden et al., 2019). To exclude false positive results, we corrected the MR results with the false discovery rate (FDR).

In addition, forest plots, scatter plots, leave-one-out, and funnel plots were used to demonstrate that the data are not heterogeneous and their stability.

## 3 Results

### 3.1 SNP selection

**Figure 3:**
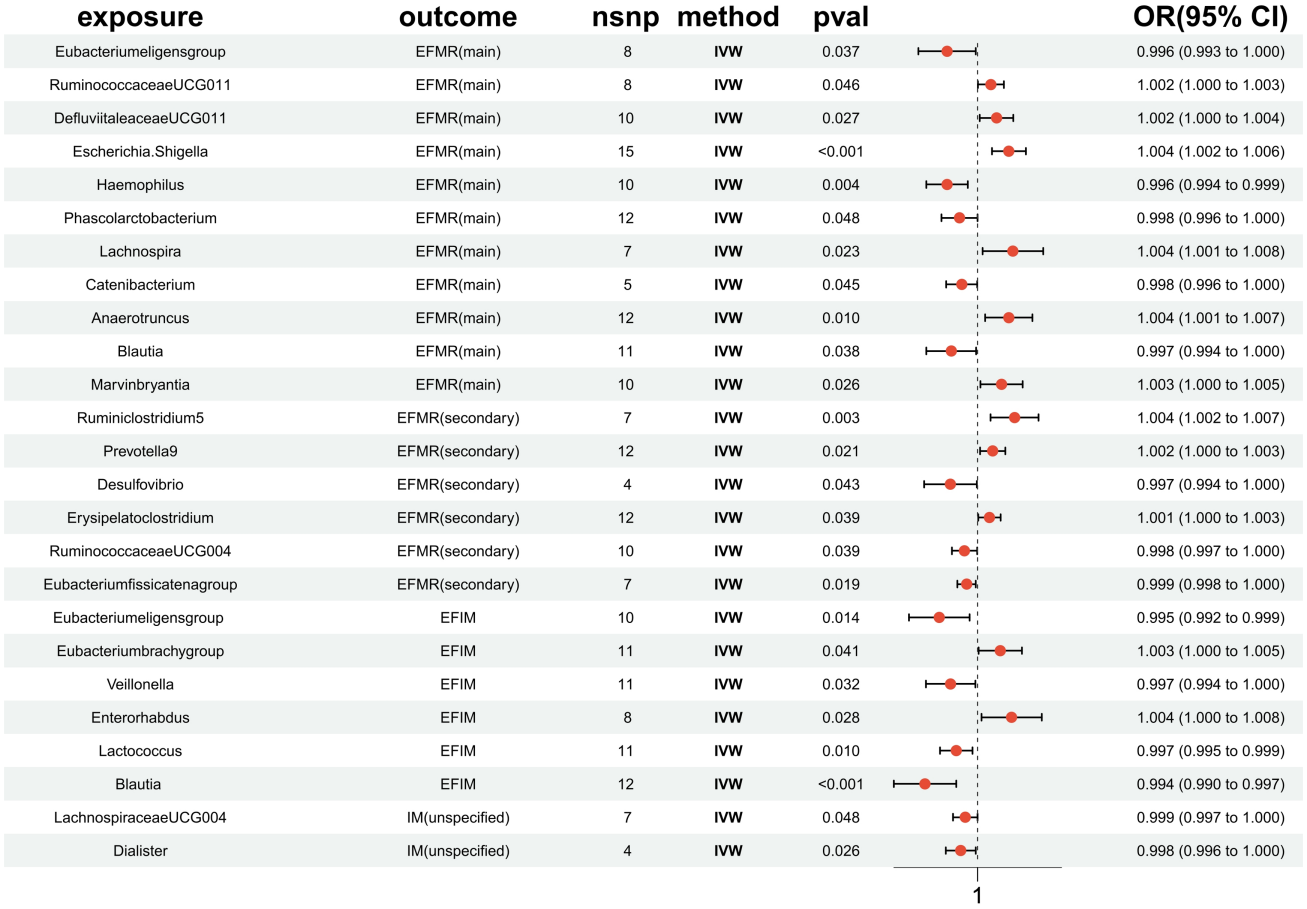
MR results of causal effects between gut microbiota and menstrual disorders. *p*-value, OR, odds ratio.

In total, 1,531 SNPs were employed for 119 different bacterial species as IVs in accordance with the selection criteria for Ivs. A total of 25 results were obtained (Figure 3). The F-statistics for the Ivs were all more than 10, respectively. Cochrane’s Q test did not reveal any evidence of heterogeneity in the sensitivity analysis (Supplementary Table S1). The MR-PRESSO global test (*p* > 0.05) also did not find evidence of a multibiotic effect (Supplementary Table S1). Ultimately, after eliminating the polytomous SNPs identified by the MR-PRESSO outlier test (*p* > 0.05) and MR-Egger regression (*p* > 0.05), there was no evidence of horizontal pleiotropy of IVs (Supplementary Table S1). The forest plots, funnel plots, leave-one-out plots, and scatter plots are shown in Supplementary Figures S1–S4.

### 3.2 EFMR (main)

This study identified 11 gut microbiota were found to be associated with EFMR (main) in IVW (Figure 3). Specifically, IVW estimate suggests that *Eubacterium eligens group* (OR = 0.996, 95%CI = 0.993–1.000, *p* = 0.037), *Haemophilus* (OR = 0.996, 95%CI = 0.994–0.999, *p* = 0.004), *Phascolarcto bacterium* (OR = 0.998, 95%CI = 0.996–1.000, *p* = 0.048), *Cateni bacterium* (OR = 0.998, 95%CI = 0.996–1.000, *p* = 0.045), and *Blautia* (OR = 0.997, 95%CI = 0.994–1.000, *p* = 0.038) had a protective effect on this type of menstrual disorders (Table 1). *RuminococcaceaeUCG011* (OR = 1.002, 95%CI = 1.000–1.003, *p* = 0.046), *DefluviitaleaceaeUCG011* (OR = 1.002, 95%CI = 1.000–1.004, *p* = 0.027), *Escherichia/Shigella* (OR = 1.004, 95%CI = 1.002–1.006, *p* = 0.0003), *Lachnospira* (OR = 1.004, 95%CI = 1.001–1.008, *p* = 0.023), *Anaerotruncus* (OR = 1.004, 95%CI = 1.001–1.007, *p* = 0.010), and *Marvinbryantia* (OR = 1.003, 95%CI = 1.000–1.005, *p* = 0.026) had a negative effect on this type of menstrual disorders (Table 1).

**Table 1:** MR results of causal effects between gut microbiota and EFMR (main).

Disease type (outcome)	Gut microbiota (exposure)	Method	nSNPs	Beta	SE	*p-*value	OR	95%CI
EFMR (main)	Eubacteriumeligens group	IVW	8	−0.004	0.002	0.037	0.996	0.993–1.000
	RuminococcaceaeUCG011	IVW	8	0.002	0.001	0.046	1.002	1.000–1.003
	DefluviitaleaceaeUCG011	IVW	10	0.002	0.001	0.027	1.002	1.000–1.004
	Escherichia/Shigella	IVW	15	0.004	0.001	0.0003	1.004	1.002–1.006
	Haemophilus	IVW	10	−0.004	0.001	0.004	0.996	0.994–0.999
	Phascolarctobacterium	IVW	12	−0.002	0.001	0.048	0.998	0.996–1.000
	Lachnospira	IVW	7	0.004	0.002	0.023	1.004	1.001–1.008
	Catenibacterium	IVW	5	−0.002	0.001	0.045	0.998	0.996–1.000
	Anaerotruncus	IVW	12	0.004	0.001	0.010	1.004	1.001–1.007
	Blautia	IVW	11	−0.003	0.002	0.038	0.997	0.994–1.000
	Marvinbryantia	IVW	10	0.003	0.001	0.026	1.003	1.000–1.005

**Figure 4:**
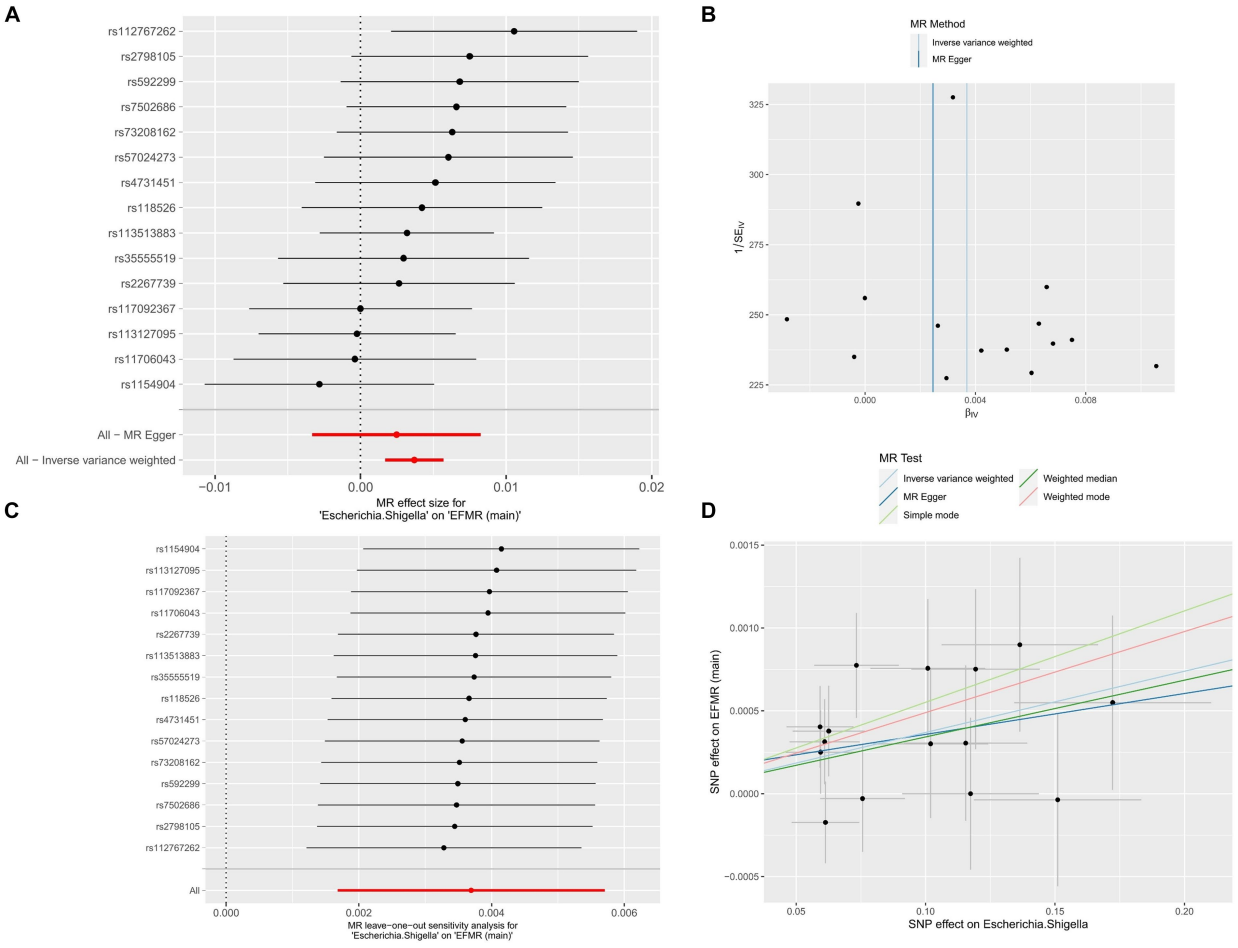
MR results of causal effects between Escherichia/Shigella and EFMR (main). **(A)** The forest plot of Escherichia/Shigella on EFMR (main). **(B)** The funnel plot of Escherichia/Shigella on EFMR (main). **(C)** The leave-one-out plot of Escherichia/Shigella on EFMR (main). **(D)** The scatter plot of Escherichia/Shigella on EFMR (main).

After FDR correction, the results showed that only Escherichia/Shigella (*p* = 0.00032, *P_FDR_* = 0.0382, OR = 1.004, 95%CI = 1.002–1.006) was related to EFMR (main). The forest plots, funnel plots, leave-one-out plots, and scatter plots of Escherichia/Shigella proved the stability of the results (Figure 4).

### 3.3 EFMR (secondary)

Our study identified six gut microbiota were found to be associated with EFMR (secondary) in IVW (Figure 3). IVW estimate suggests that *Desulfovibrio* (OR = 0.997, 95%CI = 0.994–1.000, *p* = 0.043), *RuminococcaceaeUCG004* (OR = 0.998, 95%CI = 0.997–1.000, *p* = 0.039), and *Eubacterium fissicatena group* (OR = 0.999, 95%CI = 0.998–1.000, *p* = 0.019) had a protective effect (Table 2). *Ruminiclostridium5* (OR = 1.004, 95%CI = 1.002–1.007, *p* = 0.003), *Prevotella9* (OR = 1.002, 95%CI = 1.000–1.003, *p* = 0.021), and *Erysipelatoclostridium* (OR = 1.001, 95%CI = 1.000–1.003, *p* = 0.039) had a negative effect on this type of menstrual disorders (Table 2).

**Table 2:** MR results of causal effects between gut microbiota and EFMR (secondary).

Disease type (outcome)	Gut microbiota (exposure)	Method	nSNPs	Beta	SE	*p-*value	OR	95%CI
EFMR (secondary)	Ruminiclostridium5	IVW	7	0.004	0.001	0.003	1.004	1.002–1.007
	Prevotella9	IVW	12	0.002	0.001	0.021	1.002	1.000–1.003
	Desulfovibrio	IVW	4	−0.003	0.002	0.043	0.997	0.994–1.000
	Erysipelatoclostridium	IVW	12	0.001	0.001	0.039	1.001	1.000–1.003
	RuminococcaceaeUCG004	IVW	10	−0.002	0.001	0.039	0.998	0.997–1.000
	Eubacteriumfissicatena group	IVW	7	−0.001	0.001	0.019	0.999	0.998–1.000

After FDR correction, we did not find a causal relationship between gut microbiota and EFMR (secondary).

### 3.4 EFIM

Six causal associations from gut microbiota to EFIM were identified by the IVW method (Figure 3). IVW estimate suggests that *Eubacterium eligens group* (OR = 0.995, 95%CI = 0.992–0.999, *p* = 0.014), *Veillonella* (OR = 0.997, 95%CI = 0.994–1.000, *p* = 0.032), *Lactococcus* (OR = 0.997, 95%CI = 0.995–0.999, *p* = 0.010), and *Blautia* (OR = 0.994, 95%CI = 0.990–0.997, *p* = 0.001) had a protective effect on this type of menstrual disorders (Table 3). *Eubacterium brachy group* (OR = 1.003, 95%CI = 1.000–1.005, *p* = 0.041) and *Enterorhabdus* (OR = 1.004, 95%CI = 1.000–1.008, *p* = 0.028) had a negative effect on this type of menstrual disorders (Table 3).

**Table 3:** MR results of causal effects between gut microbiota and EFIM.

Disease type (outcome)	Gut microbiota (exposure)	Method	nSNPs	Beta	SE	*p-*value	OR	95%CI
EFIM	Eubacteriumeligens group	IVW	10	−0.005	0.002	0.014	0.995	0.992–0.999
	Eubacteriumbrachy group	IVW	11	0.003	0.001	0.041	1.003	1.000–1.005
	Veillonella	IVW	11	−0.003	0.002	0.032	0.997	0.994–1.000
	Enterorhabdus	IVW	8	0.004	0.002	0.028	1.004	1.000–1.008
	Lactococcus	IVW	11	−0.003	0.001	0.010	0.997	0.995–0.999
	Blautia	IVW	12	−0.006	0.002	0.001	0.994	0.990–0.997

After FDR correction, we did not find a causal relationship between gut microbiota and EFIM.

### 3.5 IM (unspecified)

Two causal associations from gut microbiota to IM (unspecified) were identified (Figure 3). IVW estimate suggests that *LachnospiraceaeUCG004* (OR = 0.999, 95%CI = 0.997–1.000, *p* = 0.048) and *Dialister* (OR = 0.998, 95%CI = 0.996–1.000, *p* = 0.026) had a protective effect on this type of menstrual disorders (Table 4).

**Table 4:** MR results of causal effects between gut microbiota and IM.

Disease type (outcome)	Gut microbiota (exposure)	Method	nSNPs	Beta	SE	*p-*value	OR	95%CI
IM (unspecified)	LachnospiraceaeUCG004	IVW	7	−0.001	0.001	0.048	0.999	0.997–1.000
	Dialister	IVW	4	−0.002	0.001	0.026	0.998	0.996–1.000

After FDR correction, we did not find a causal relationship between gut microbiota and IM (unspecified).

## 4 Discussion

In order to determine the causal link between gut microbiota and menstrual disorders, we conducted a two-sample MR analysis in this study using gut microbiota summary statistics from the largest GWAS meta-analysis carried out by the MiBioGen consortium and “Menstrual disorders” summary statistics from the MRC-IEU, NA release data. As shown in our results, a total of 23 different kinds of gut microbiota have an effect on menstrual disorders [*Eubacteriumeligens group*, *Blautia* is repeated in EFMR (main) and EFIM], of which 12 intestinal flora are protective factors for menorrhagia, while the other 11 are risk factors for menorrhagia. While 23 groups of bacteria had a tentative association, only *Escherichia/Shigella* had a significant robust effect. After FDR correction, only *Escherichia/Shigella* was causally associated with menstrual disorders, as it increases, so does the risk of disease.

*Escherichia/Shigella* is an *Enterobacteriaceae* bacterium that has been shown to cause intestinal inflammation and increased intestinal permeability when overexpressed, making it a recognized pathogenic bacterium (Mukherjee et al., 2023). Its secreted lipopolysaccharide induces acute intestinal injury, increases blood–brain barrier permeability, and activates neuroinflammation (Dong et al., 2021). Some scholars have found that the proportion of *Escherichia/Shigella* and the number of *Streptococcus* in intestinal growth, whereas the number of *Akkermansia* and *Ruminococcaceae* decreases in patients with Polycystic ovary syndrome (Liu et al., 2022). An increase in the number of *Escherichia coli* and *Shigella* in the gut microbiome of patients with stage IIIIV endometriosis was found (Ata et al., 2019; Cao et al., 2024). *Escherichia coli* can also cause chronic endometritis (Cao et al., 2024). To our knowledge, this is the first time *Escherichia/Shigella* has been found to negatively affect menstruation. This could provide new ideas for future research and treatment of menstrual disorders.

Ravel and Brotman proposed the term “gut-vagina axis” in 2016 (Ravel and Brotman, 2016). In a previous study, 68% of the 63 bacterial species that were detected in the vagina or rectum had the same genotype, with 44% of the species present in both organs (El Aila et al., 2009), and species-level Spearman correlation coefficient analysis was used to identify individual BV-associated bacteria in the rectum and vagina of pregnant women (Fudaba et al., 2021). Oral probiotic strains were also found in the vagina (Reid et al., 2001; Morelli et al., 2004; Strus et al., 2012; Russo et al., 2018). These findings imply that vaginal germs may be able to be preserved in the rectum.

On the other hand, metabolites generated by the microbiota, such as short-chain fatty acids (SCFAs), may be viewed as collateral players in the gut-vaginal axis. There is no doubt that SCFAs have distinct functions in the gut and vagina (Amabebe and Anumba, 2020). While SCFAs in the vagina are linked to dysfunctional and pro-inflammatory states, SCFAs in the stomach serve advantageous purposes such maintaining barrier function (Aldunate et al., 2015). Due to the systemic circulation can carry SCFAs produced by gut bacteria to other organs (Dalile et al., 2019), it is possible that SCFAs have a role in the vaginal-gut axis. It is believed that vaginal bacteria’s production of short-chain fatty acids adds to the dysbiotic environment (Aldunate et al., 2015). Excess short-chain fatty acids may be a possible cause of cervicovaginal inflammation, according to an *in vitro* investigation (Delgado-Diaz et al., 2020). Therefore, vaginal microecological dysregulation brought on by the flow of short-chain fatty acids from the gut to the vagina may cause menstruation abnormalities (Takada et al., 2023).

The estrobolome has the ability to deconjugate hepatically conjugated estrogens in the gastrointestinal system in addition to metabolites. Deconjugated estrogen is then reabsorbed to the systemic circulation. When circulating estrogen reaches the distal epithelium of the vagina, it modifies the physiological traits of the cells that line the vagina, including the generation of mucus and glycogen. Since glycogen can be a vital source of energy for *Lactobacilli*, increased glycogen promotes *Lactobacillus* dominance in the vagina (Witkin, 2018; Linhares et al., 2019; Takada et al., 2023). Therefore, the number of *Lactobacillus* in the vaginal microbiota can be influenced by the number of bacteria in the gut microbiota that metabolize estrogen.

Evolutionary studies suggest that genes encoding the enzymes that catalyze the metabolism of dopamine, norepinephrine, and serotonin may have been passed from bacteria to host cells during the course of evolution (Iyer et al., 2004). Hormone receptors have even been detected on some microorganisms (Lyte, 1993; Nabeh, 2023). *Escherichia coli* growth depends on the transfer of iron from transferrin to bacteria, which is facilitated by catecholamines (Lyte et al., 2003). This suggests that microorganisms also interact with neurohormones, and we therefore speculate that *Escherichia/Shigella* may cause premenstrual dysphoric disorders by affecting neurohormone secretion, leading to menstrual disorders.

Fungus is also an integral part of the human body. A major member of the human fungal community has been identified as *Candida albicans* (Bradford and Ravel, 2017). *Candida albicans* can change from a commensal to a pathogenic condition when the human immune system is compromised, the intrinsic microbiota is dysregulated, or the mucosal gut barrier is compromised (Vergidis et al., 2016; Pappas et al., 2018). It has been proposed that, to differing degrees, at different phases of biofilm formation, Candida and *Escherichia coli* mutually govern the growth of biofilms (Bandara et al., 2009). *Escherichia coli* lipopolysaccharides directly regulate the production of *in vitro* biofilms, Candida species in particular, stimulating the growth of Pseudohyphae tropicalis and Nearly Naked Yeast, and inhibiting the growth of Pseudohyphae Duchenne. This stimulatory/inhibitory effect may be brought about by modifications in the number of cells inside the formed biofilm, modifications in cellular activity, or modifications in both (Bandara et al., 2009).

Cytoplasmic estrogen receptors found in certain species of *Candida* enable direct transcriptional responses to host hormones (Skowronski and Feldman, 1989). It has been demonstrated that estrogens interfere with neutrophil chemotaxis to the vaginal epithelium (Lasarte et al., 2016), and inhibit Th17 cell differentiation (Chen et al., 2015), resulting in increased host susceptibility to pathogens such as *Candida*. A hyperestrogenic state and raised vaginal pH are hallmarks of the premenstrual luteal phase, which is frequently linked to symptomatic *Candida* vaginitis (Galask, 1988). Researchers found that estrogen signaling increases *Candida albicans*’ adherence to vaginal epithelial cells (Tarry et al., 2005). Therefore, *Escherichia coli* may have an effect on estrogen through *Candida albicans*, which needs to be verified by experimental and clinical trials.

In conclusion, we conducted a thorough analysis of the connections between various menstruation disorders and the gut flora. Our findings indicated that EFMR (main) had five positive and six negative causal directions; EFMR (secondary) had three positive and three negative causal directions; EFIM had four positive and two negative causal directions; and IR had two positive causal directions. After FDR correction, the results showed that only *Escherichia/Shigella* was related to menstrual disorders. The methods by which the gut microbiota mediates the development of menstruation problems may become clearer with the help of this study. Future scholars could target *Escherichia/Shigella* for research on menstrual disorders.

## 5 Weak point

The research has certain shortcomings. First, there were no data available at the species level and only a small number of instrumental variables included in the GWAS gut flora statistics. Secondly, we were not able to ascertain if the exposures and outcomes included in this study had overlapping participants in the GWAS data. Third, just the genus level of analysis was done on bacterial taxa. Fourth, a number of gut microbiota were not included in the IV selection phase due to our criterion, which may have caused some results to be overlooked.

## Data availability statement

The datasets presented in this study can be found in online repositories. The names of the repository/repositories and accession number(s) can be found in the article/Supplementary material.

## Ethics statement

This research has been conducted using published studies and consortia providing publicly available summary statistics. All original studies have been approved by the corresponding ethical review board, and the participants have provided informed consent. In addition, no individual-level data was used in this study. Therefore, no new ethical review board approval was required. Written informed consent to participate in this study was not required from the participants or the participants’ legal guardians/next of kin in accordance with the national legislation and the institutional requirements.

## Author contributions

YY: Conceptualization, Data curation, Formal analysis, Investigation, Methodology, Software, Supervision, Validation, Visualization, Writing – original draft, Writing – review & editing. HH: Conceptualization, Data curation, Methodology, Software, Supervision, Writing – review & editing. LC: Data curation, Investigation, Methodology, Supervision, Writing – review & editing. HZ: Conceptualization, Data curation, Formal analysis, Funding acquisition, Investigation, Methodology, Project administration, Resources, Software, Supervision, Validation, Visualization, Writing – review & editing.

## Footnotes

^1^https://gwas.mrcieu.ac.uk

## Supplementary material

Supplementary Figure S1 Forest plots of EFMR (main), EFMR (secondary), EFIM, IM(unspecified).

Supplementary Figure S2 Funnel plots of EFMR (main), EFMR (secondary), EFIM, IM(unspecified)

Supplementary Figure S3 Leave-one-out plots of EFMR (main), EFMR (secondary), EFIM, IM(unspecified).

Supplementary Figure S4 Scatter plots of EFMR (main), EFMR (secondary), EFIM, IM(unspecified).

Supplementary Table S1 Results of Heterogeneity, Pleiotropy, MR-PRESSO.

Supplementary Table S2 MR results of EFMR (main), EFMR (secondary), EFIM, IM(unspecified).
