# Supplementary material for: Association between gut microbiota and menstrual disorders: a two-sample Mendelian randomization study
Source: Front Microbiol. 2024 Mar 7;15:1321268. doi: 10.3389/fmicb.2024.1321268 (PMC10954809; doi:10.3389/fmicb.2024.1321268)

MR effect size for Eubacterium eligens group on EFMR(main)

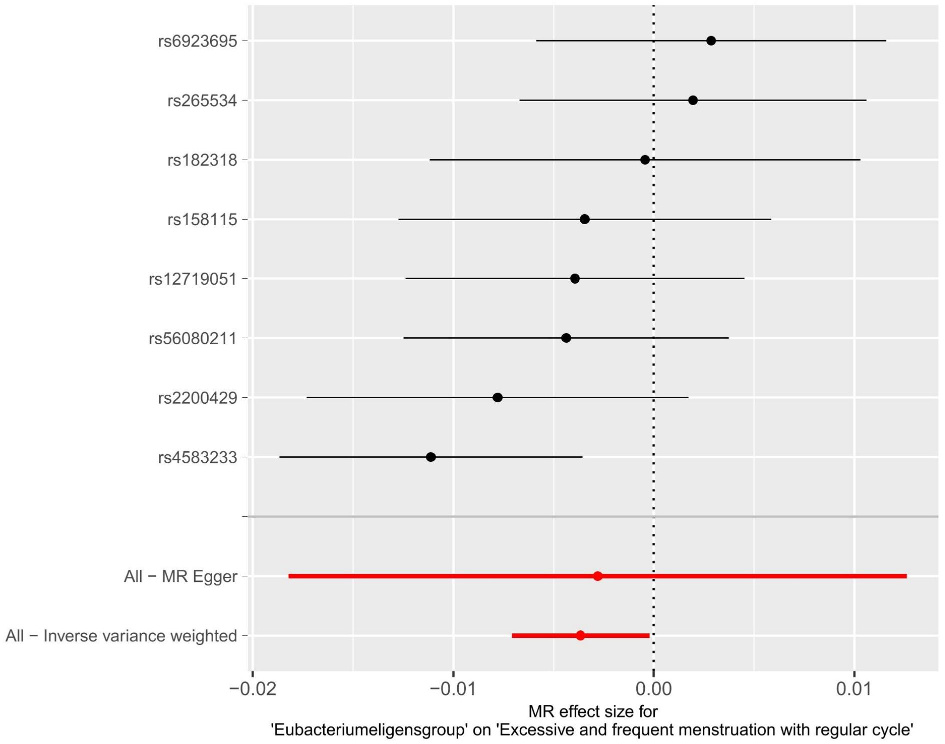

MR effect size for RuminococcaceaeUCG011 on EFMR(main)

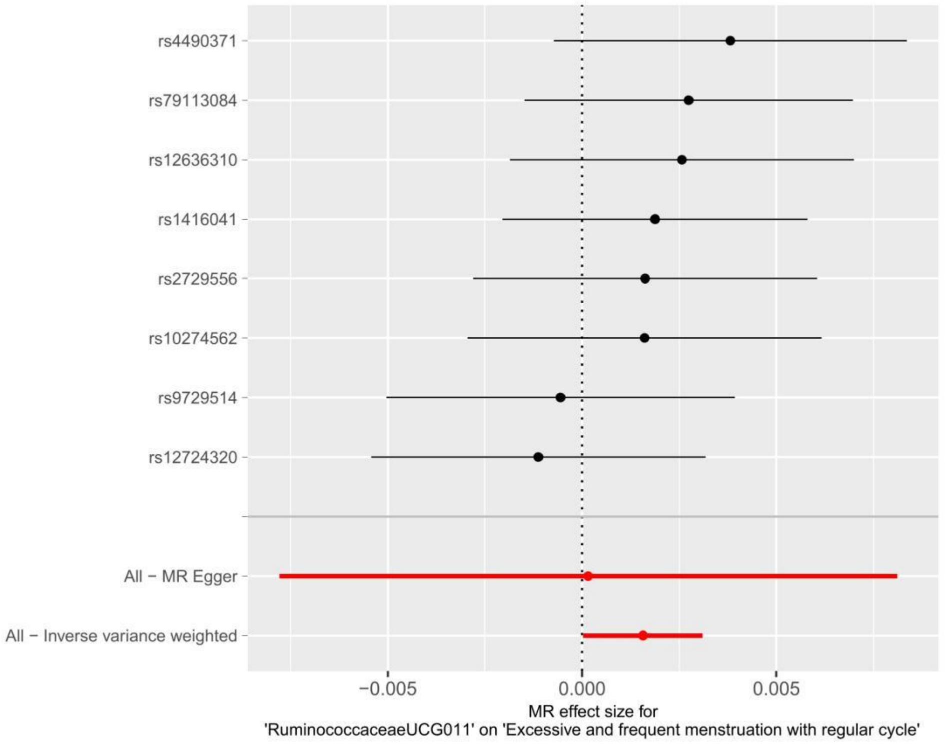

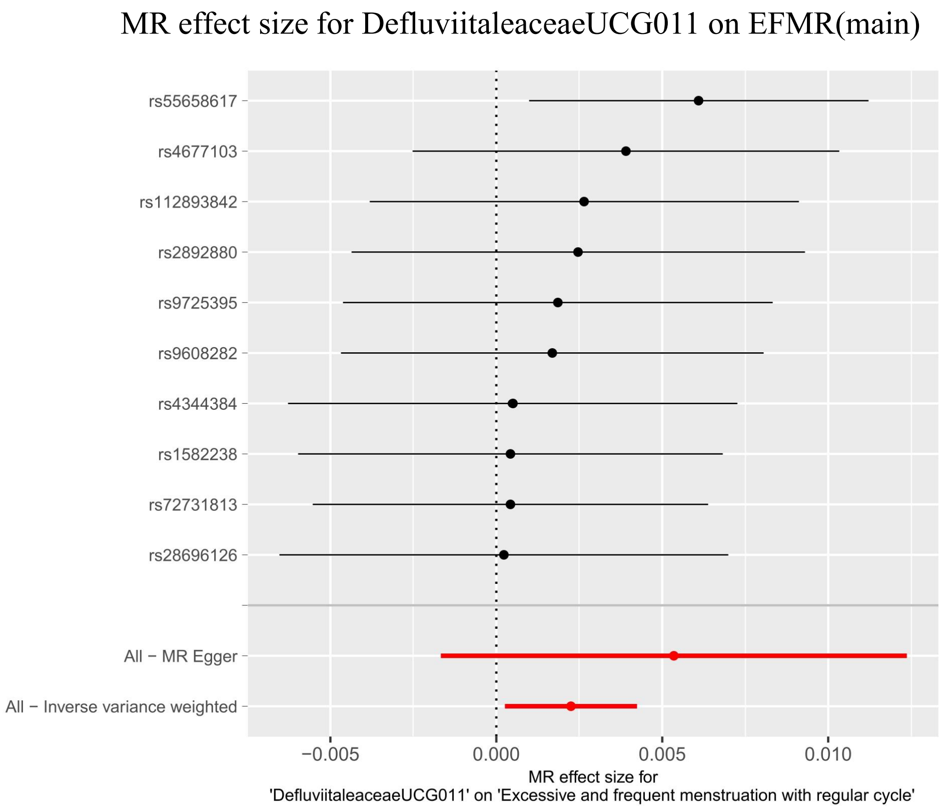
MR effect size for DefluviitaleaceaeUCG011 on EFMR(main)
rs55658617
rs4677103
rs112893842
rs2892880
rs9725395
rs9608282
rs4344384
rs1582238
rs72731813
rs28696126
All − MR Egger
All − Inverse variance weighted
−0.005
0.000
0.005
0.010
MR effect size for
'DefluviitaleaceaeUCG011' on 'Excessive and frequent menstruation with regular cycle'

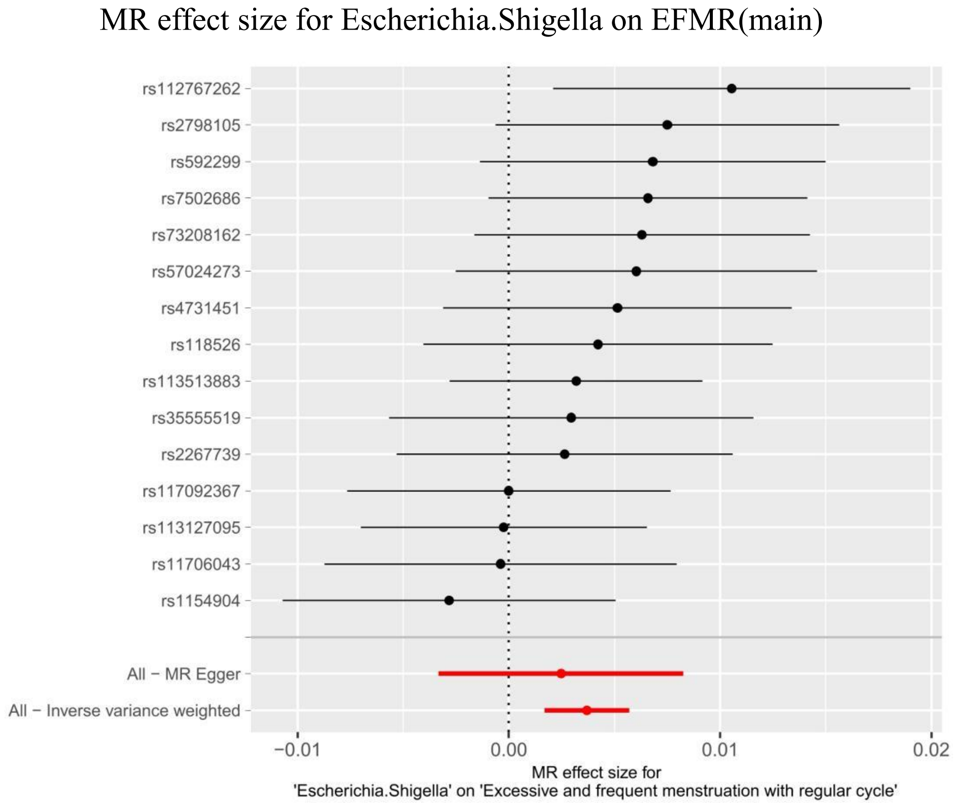
MR effect size for Escherichia.Shigella on EFMR(main)
rs112767262
rs2798105
rs592299
rs7502686
rs73208162
rs57024273
rs4731451
rs118526
rs113513883
rs35555519
rs2267739
rs117092367
rs113127095
rs11706043
rs1154904
All − MR Egger
All − Inverse variance weighted
−0.01
0.00
0.01
0.02
MR effect size for
'Escherichia.Shigella' on 'Excessive and frequent menstruation with regular cycle'

MR effect size for Haemophilus on EFMR(main)

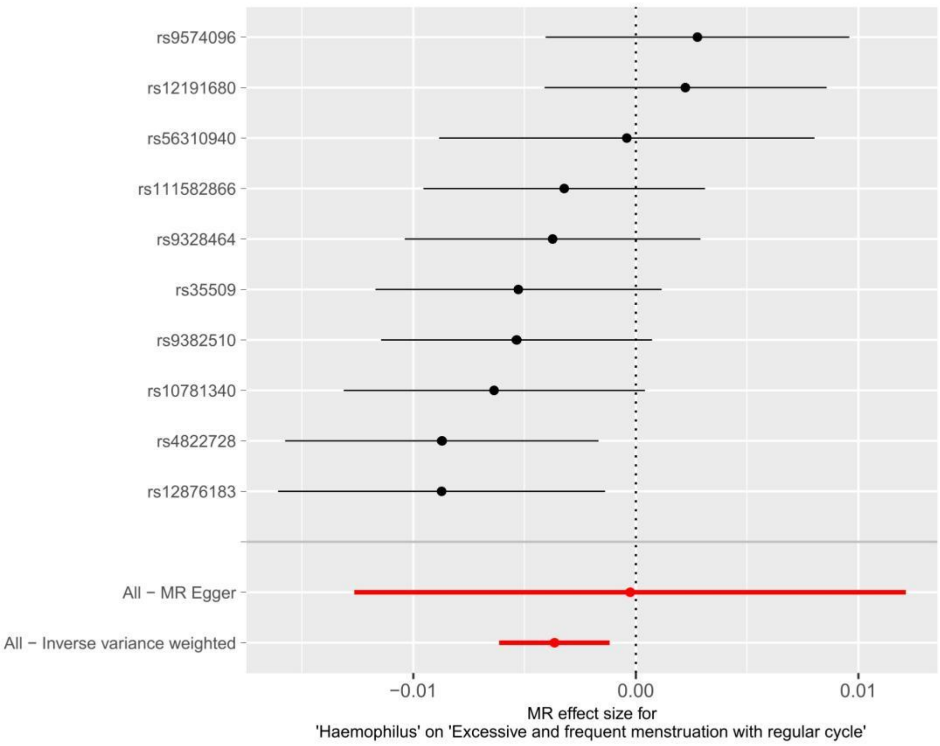

MR effect size for Phascolarcto bacterium on EFMR(main)

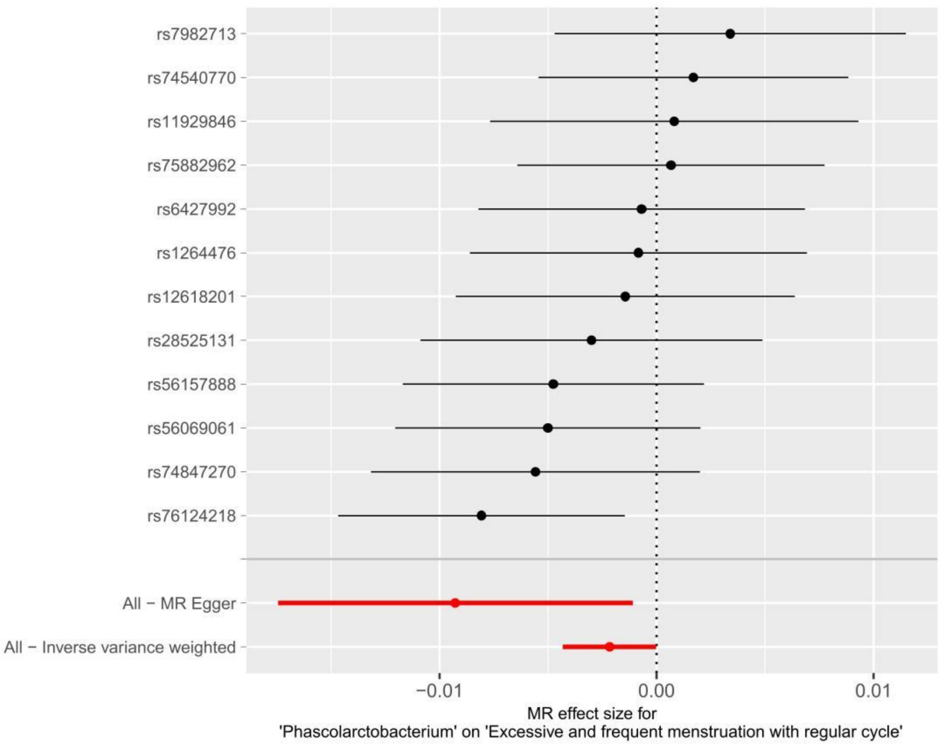

MR effect size for Lachnospira on EFMR(main)

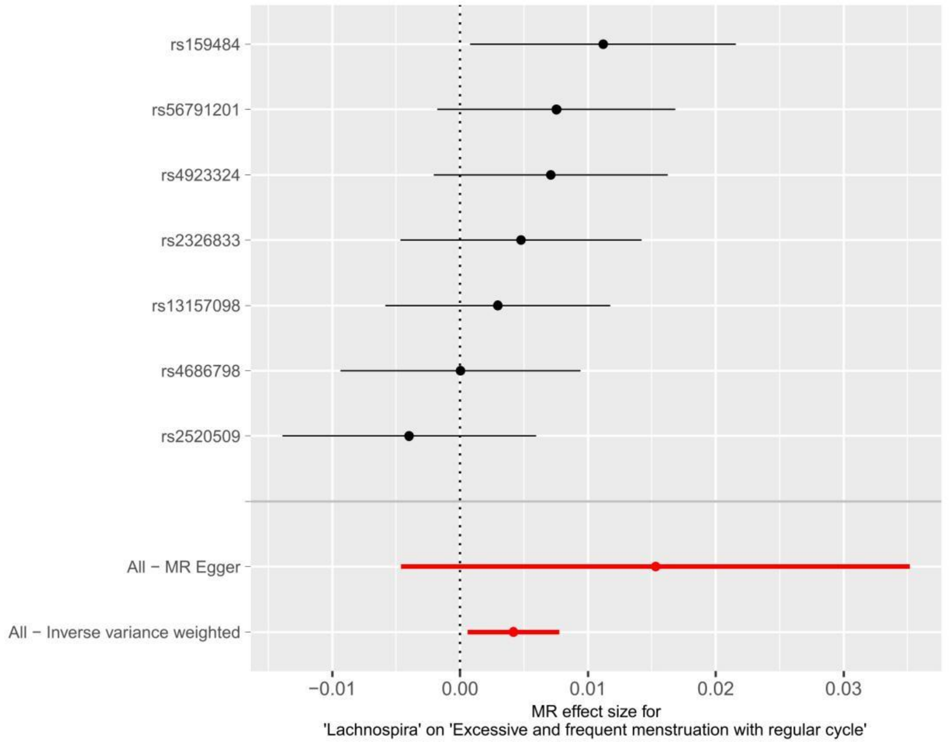

MR effect size for Cateni bacterium on EFMR(main)

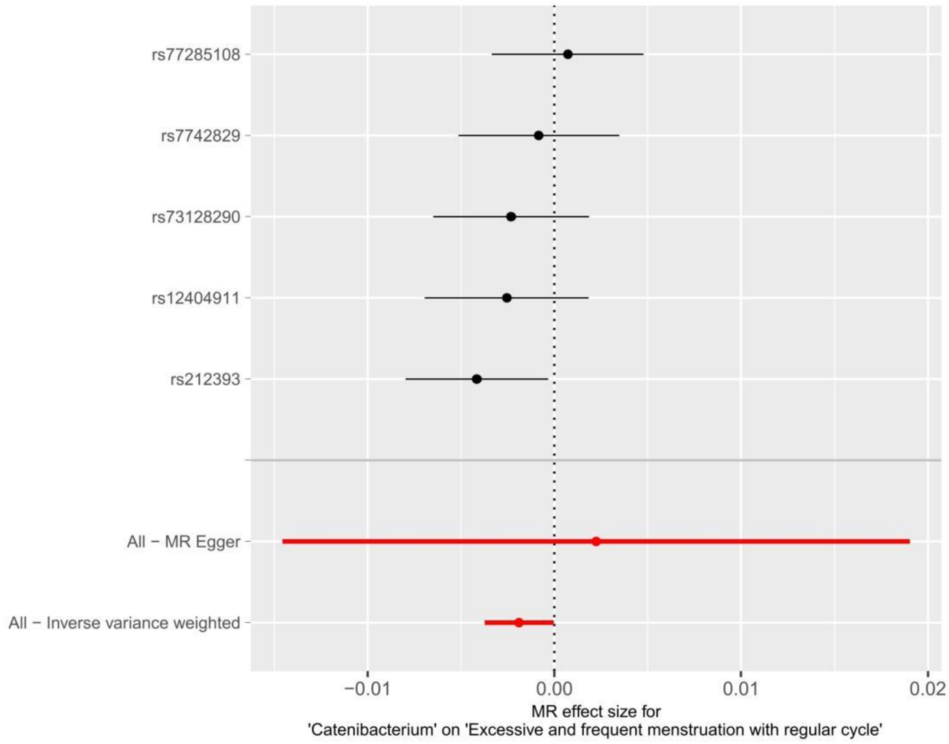

MR effect size for Anaerotruncus on EFMR(main)

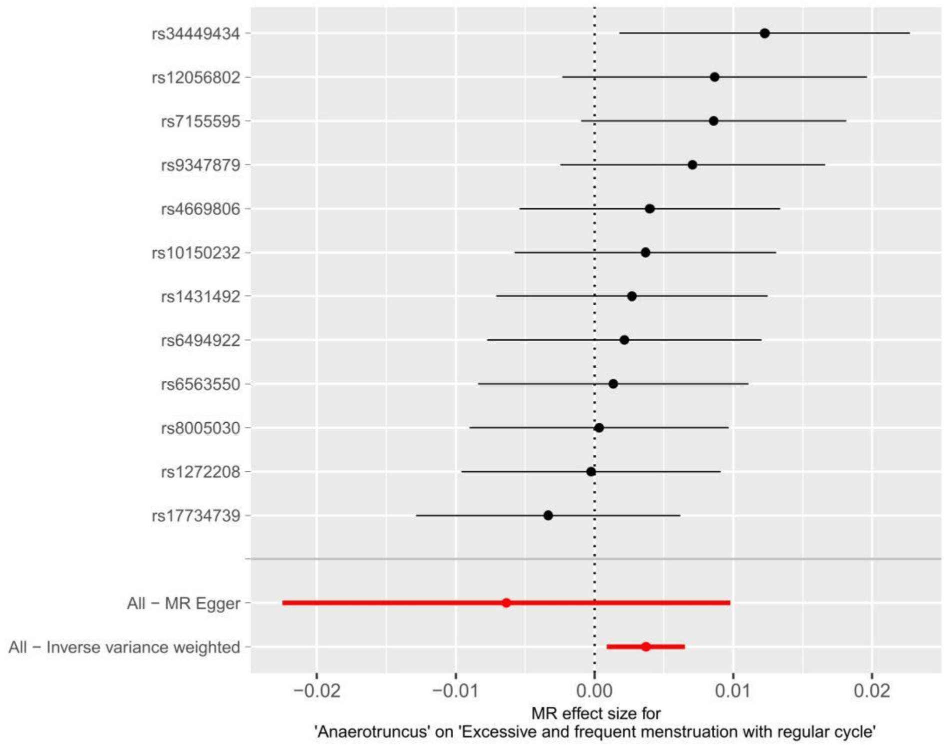

MR effect size for Blautia on EFMR(main)

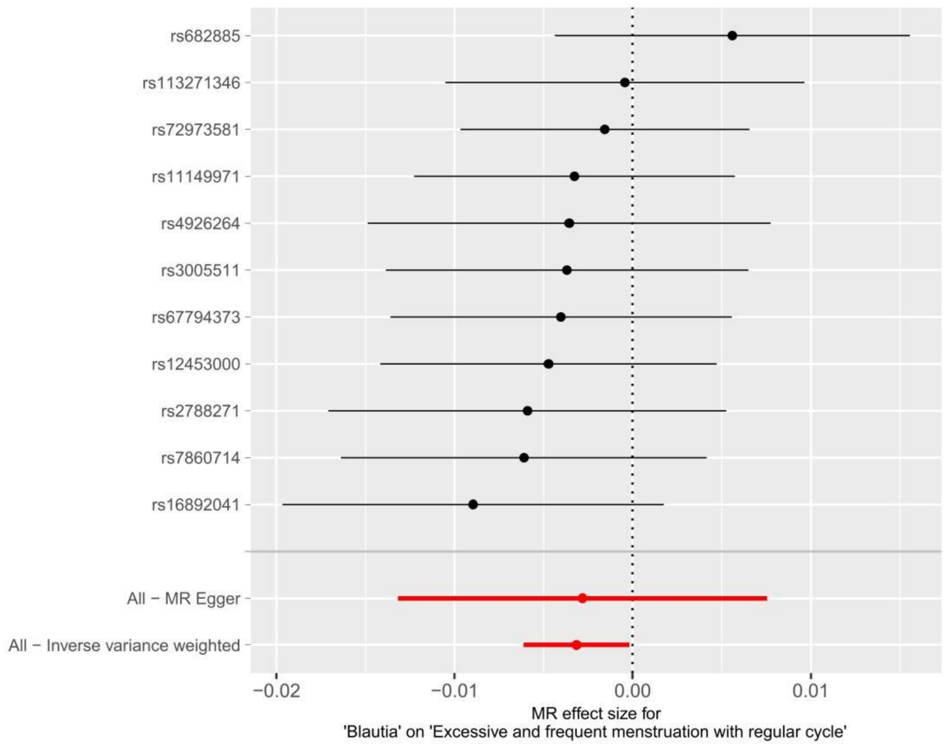

MR effect size for Marvinbryantia on EFMR(main)

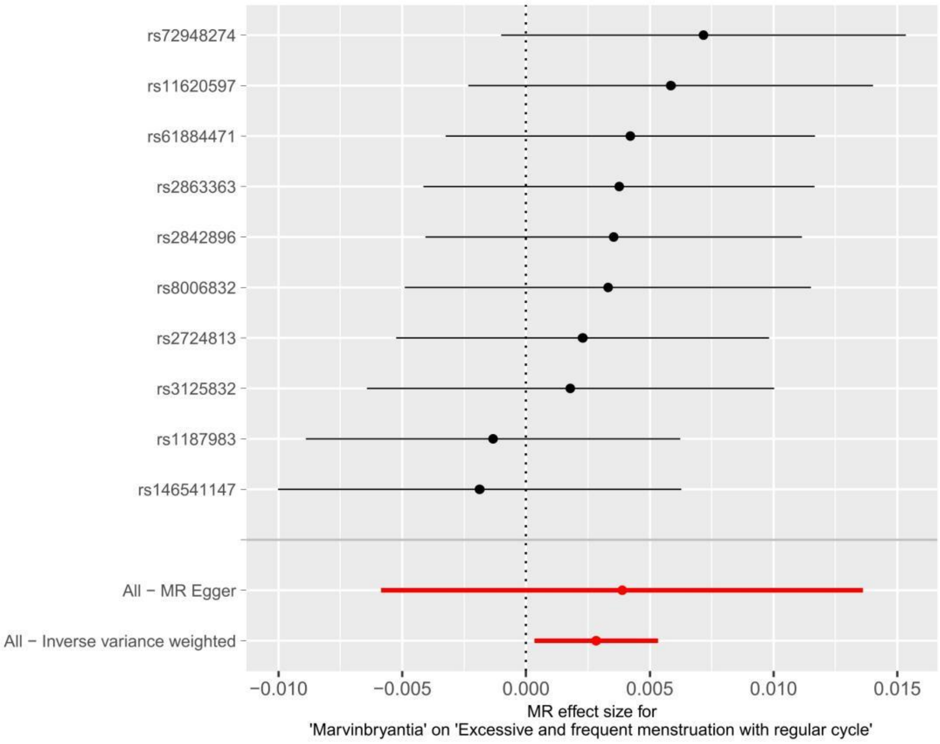

MR effect size for Ruminiclostridium5 on EFMR(secondary)

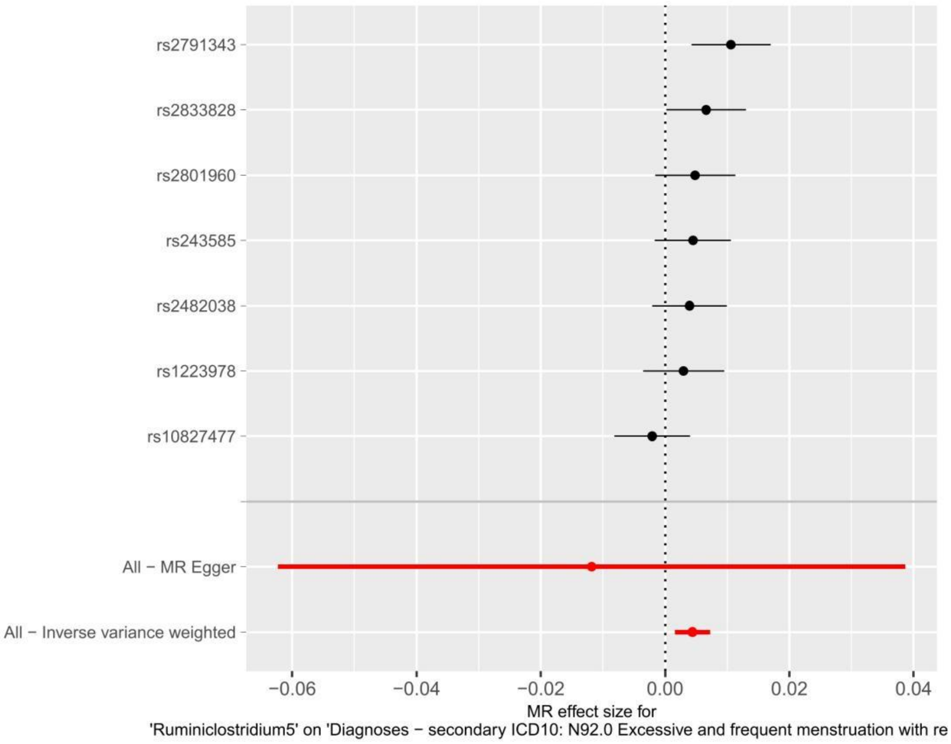

MR effect size for Prevotella9 on EFMR(secondary)

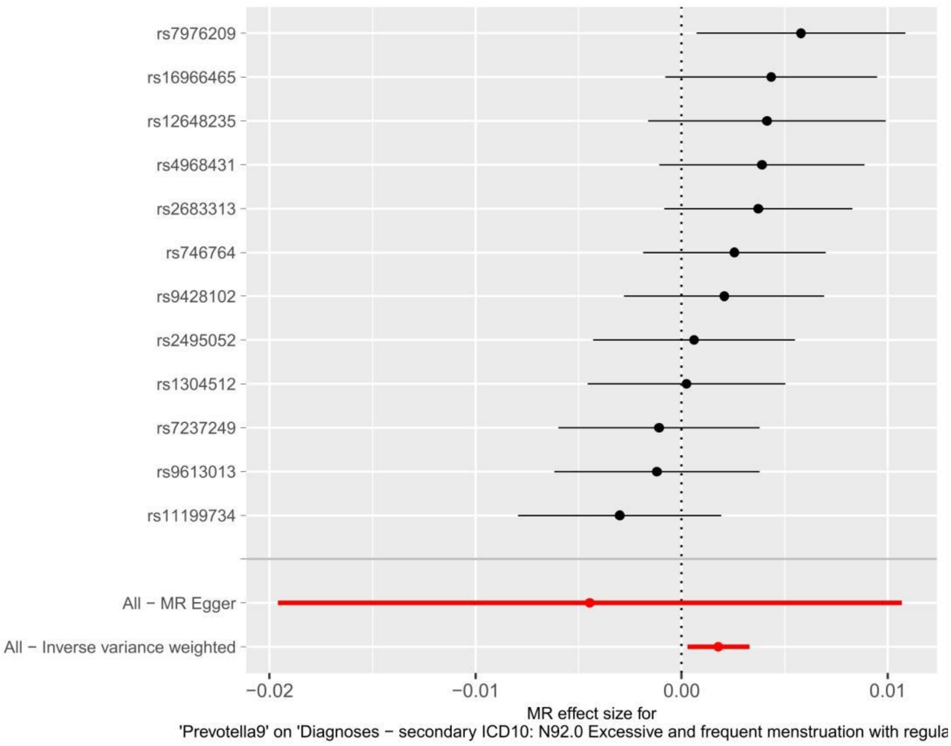

MR effect size for Desulfovibrio on EFMR(secondary)

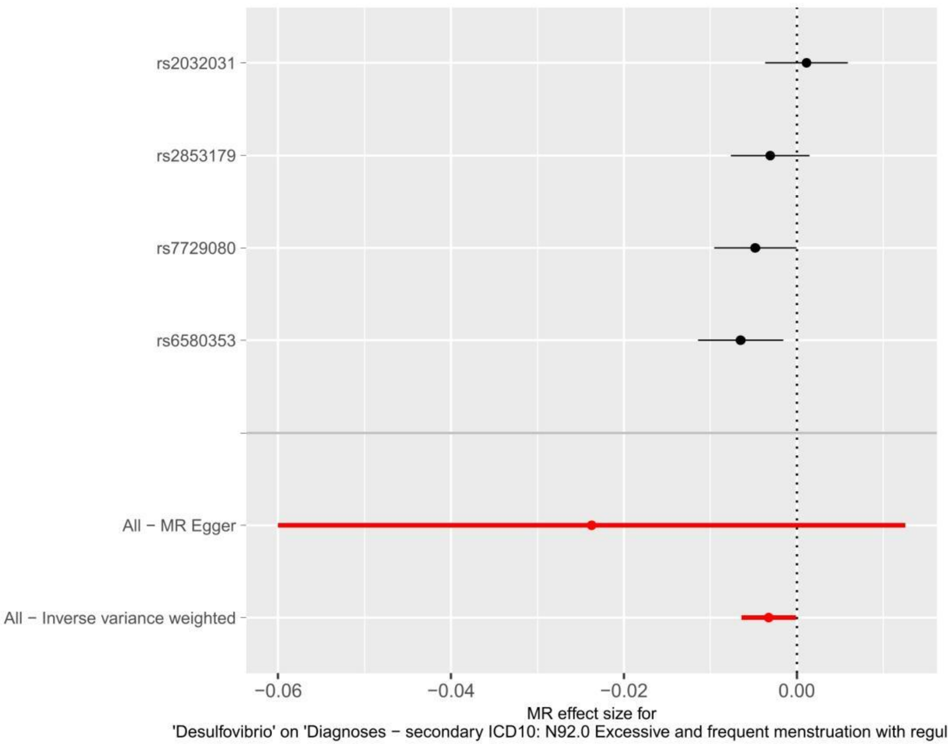

MR effect size for Erysipelatoclostridium on EFMR(secondary)

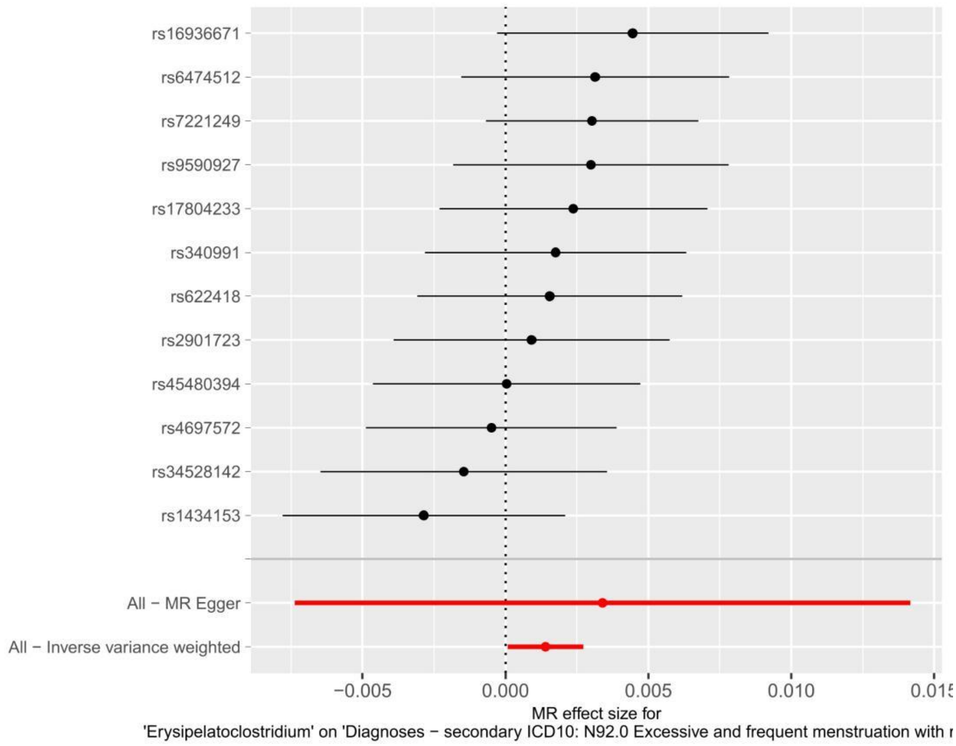

MR effect size for RuminococcaceaeUCG004 on EFMR(secondary)

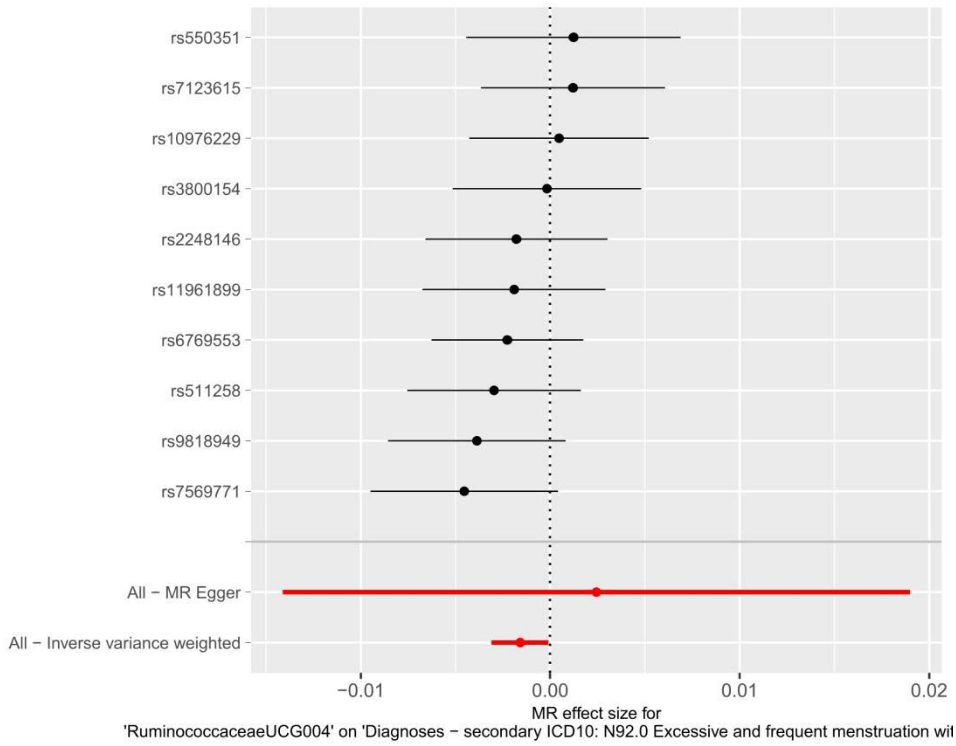

MR effect size for Eubacterium fissicatena group on EFMR(secondary)

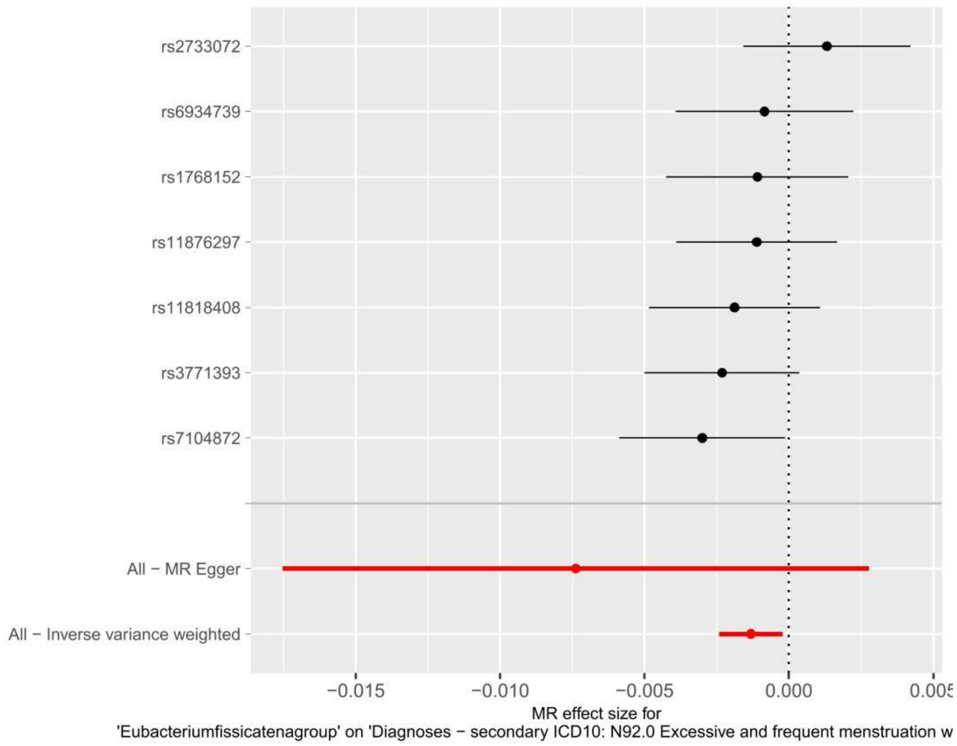

MR effect size for Eubacterium eligens group on EFIM

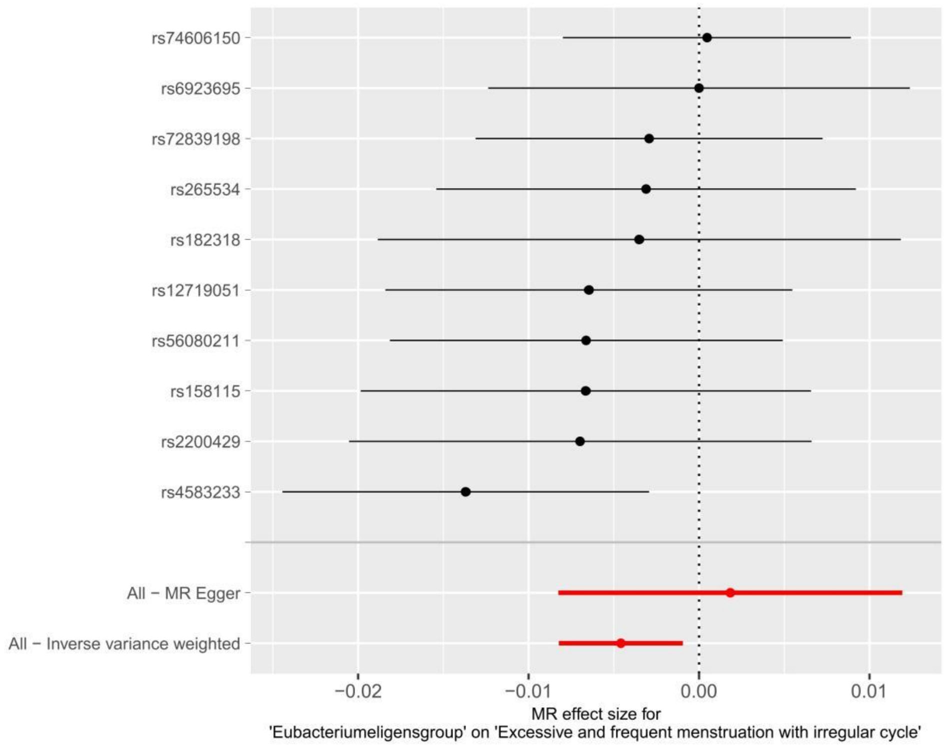

MR effect size for Eubacterium brachy group on EFIM

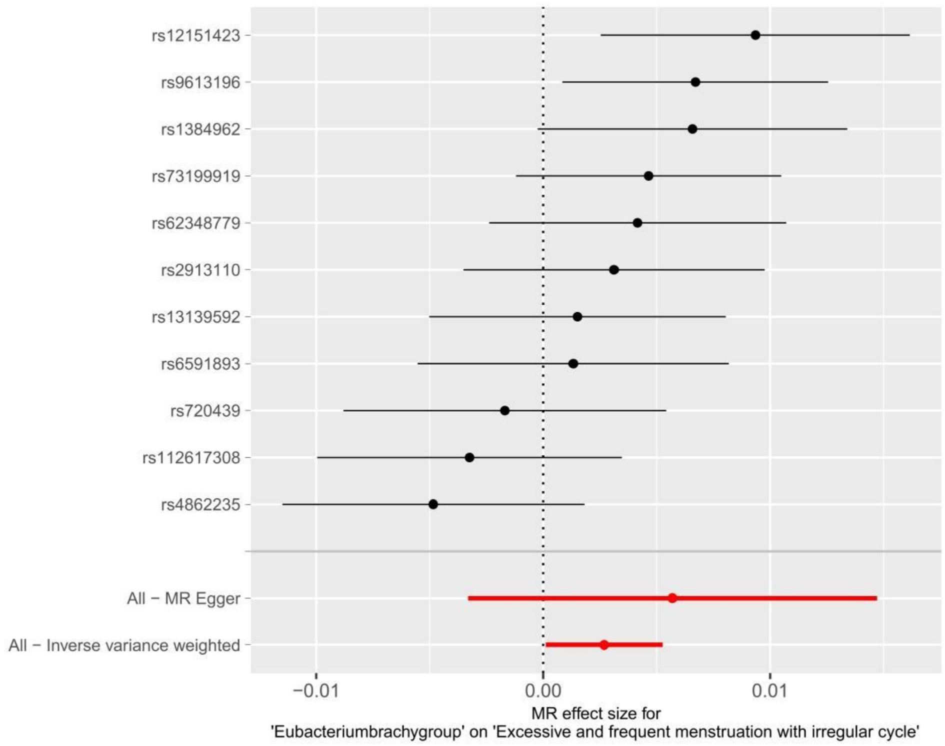

MR effect size for Veillonella on EFIM

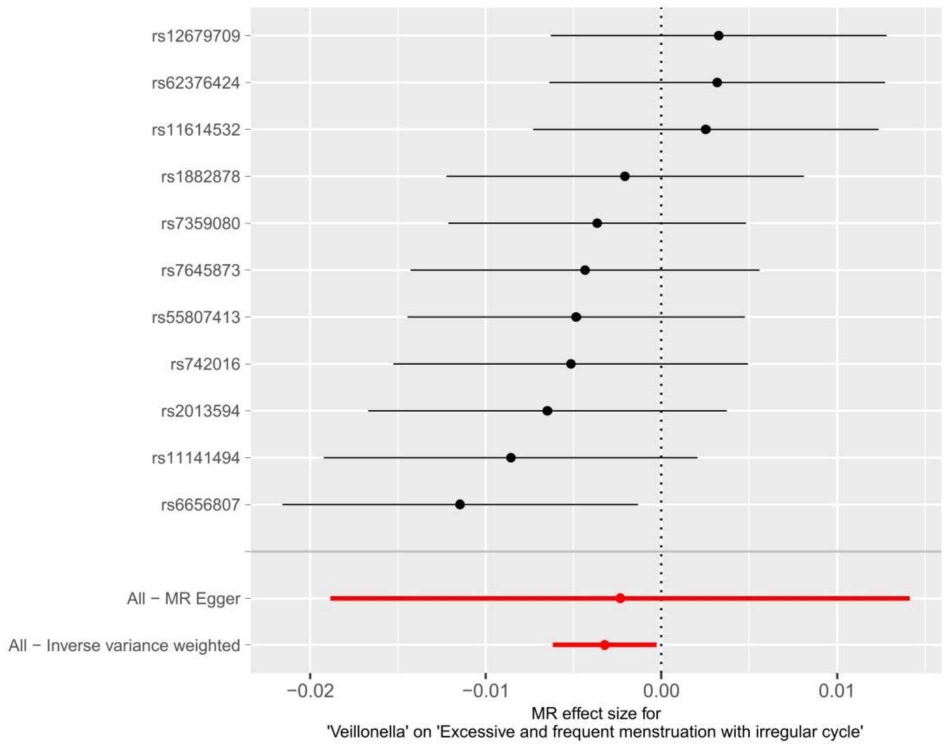

MR effect size for Enterorhabdus on EFIM

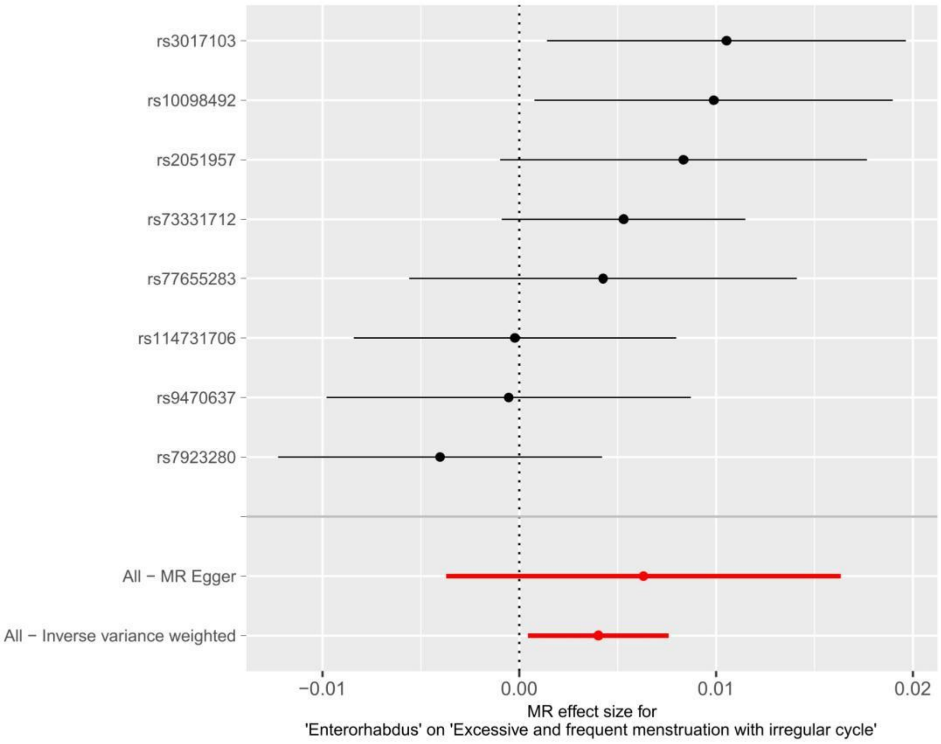

MR effect size for Lactococcus on EFIM

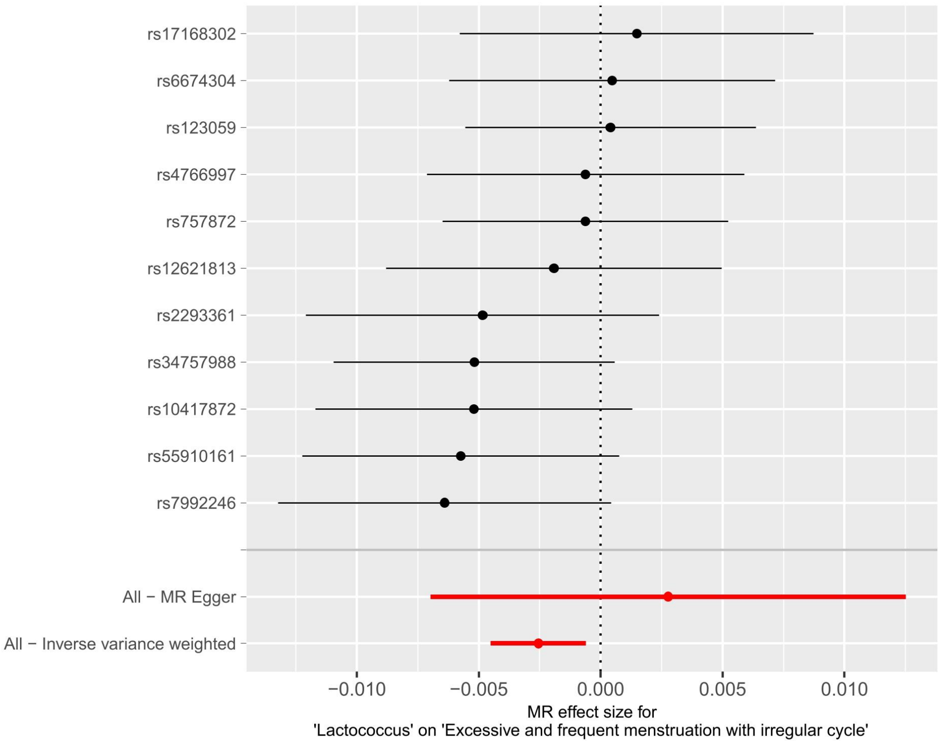

MR effect size for Blautia on EFIM

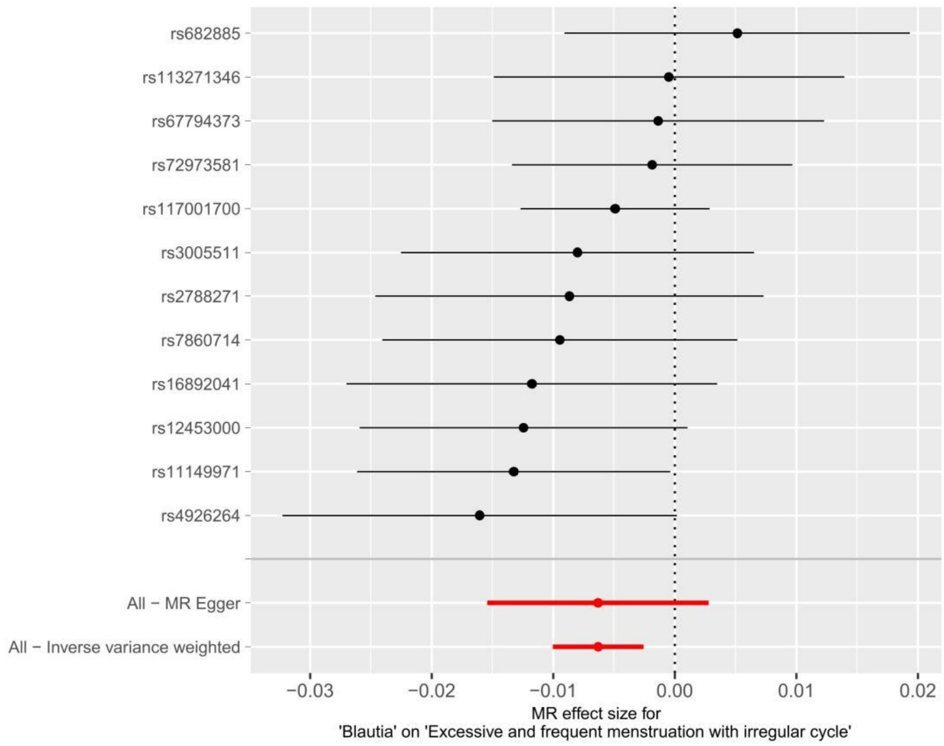

MR effect size for LachnospiraceaeUCG004 on IM(unspecified)

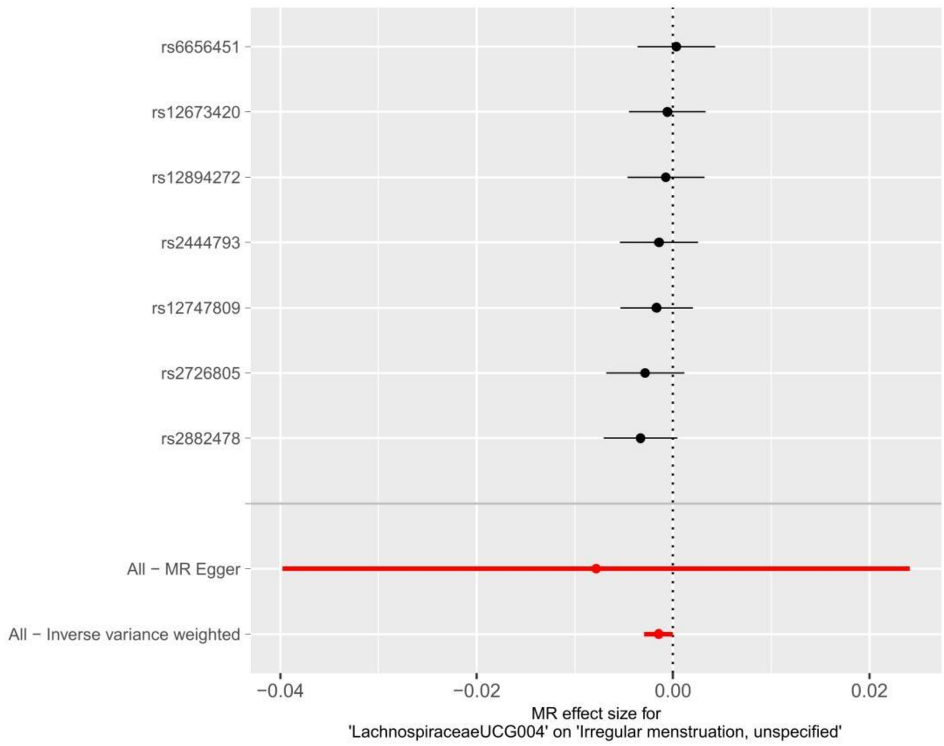

MR effect size for Dialister on IM(unspecified)

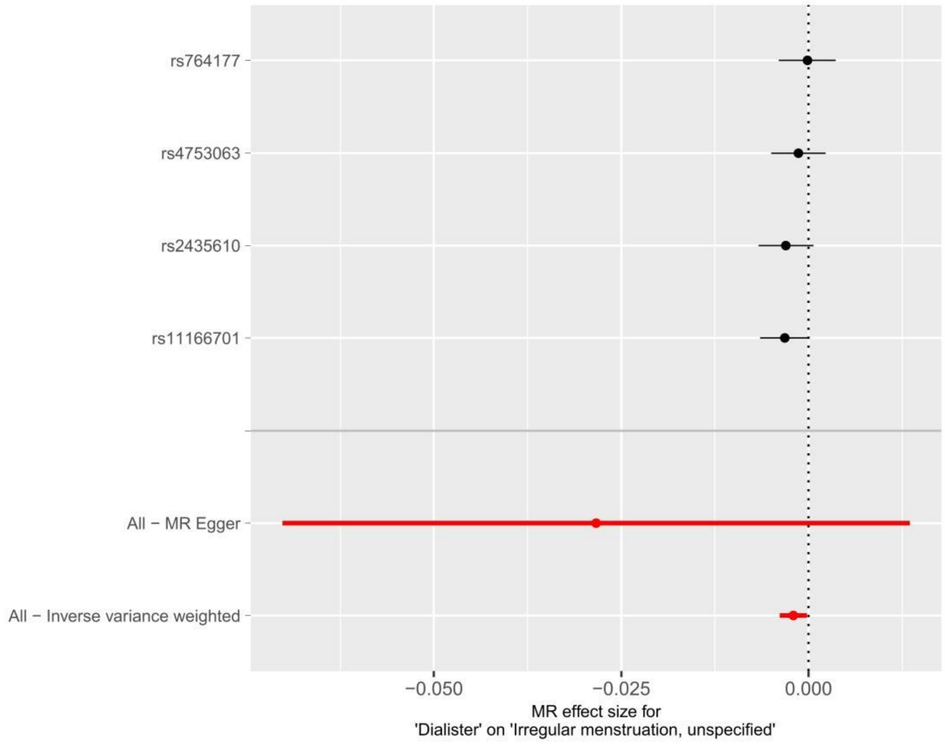

Supplement: Supplementary Figure S1 — Forest plots of EFMR (main), EFMR (secondary), EFIM, IM(unspecified). [file Data_Sheet_1.PDF]
