# Supplementary material for: Association between gut microbiota and menstrual disorders: a two-sample Mendelian randomization study
Source: Front Microbiol. 2024 Mar 7;15:1321268. doi: 10.3389/fmicb.2024.1321268 (PMC10954809; doi:10.3389/fmicb.2024.1321268)

Eubacterium eligens group. funnel_plot of EFMR(main)

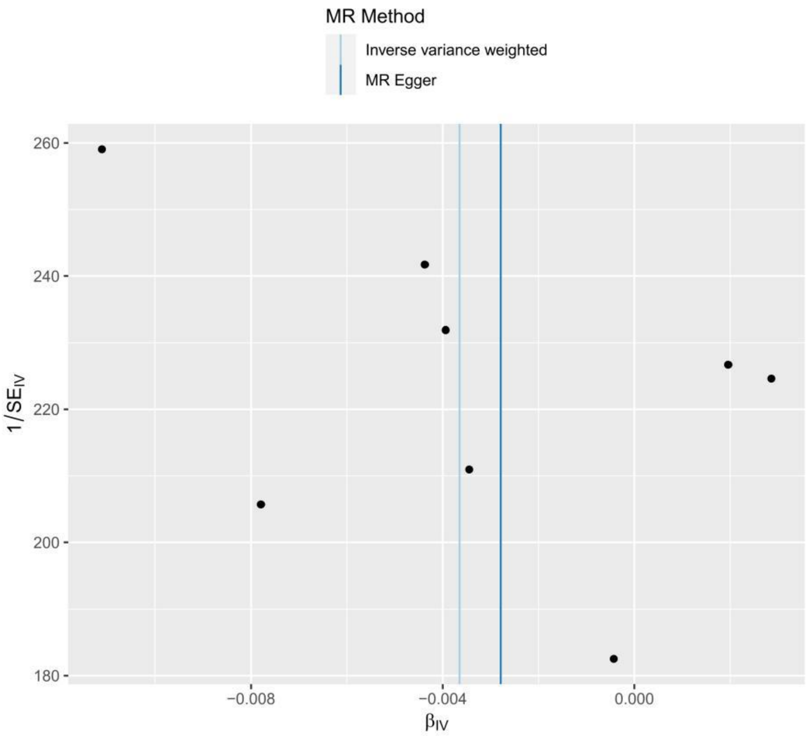

RuminococcaceaeUCG011. funnel_plot of EFMR(main)

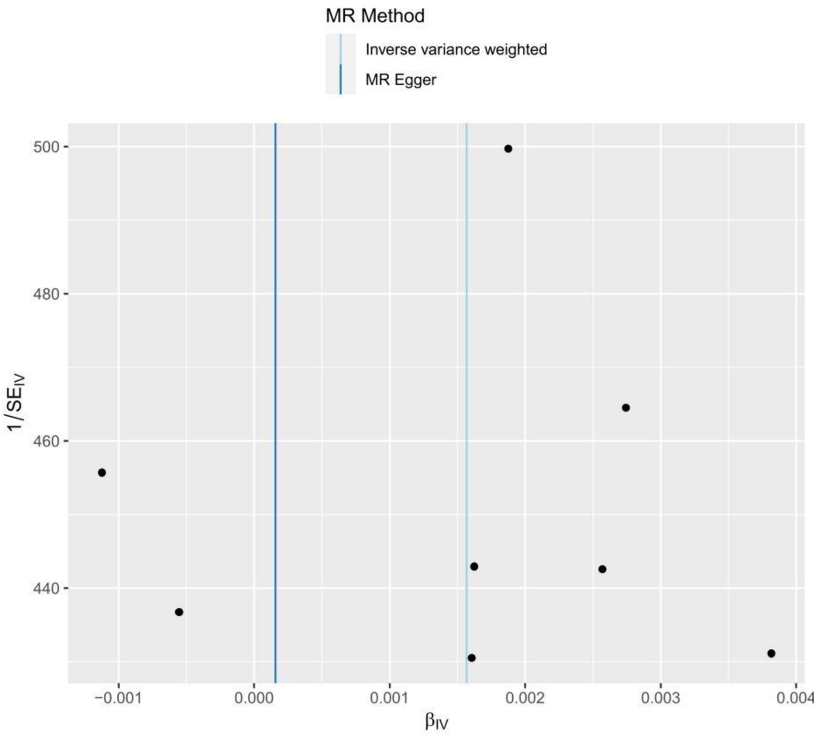

DefluviitaleaceaeUCG011. funnel_plot of EFMR(main)

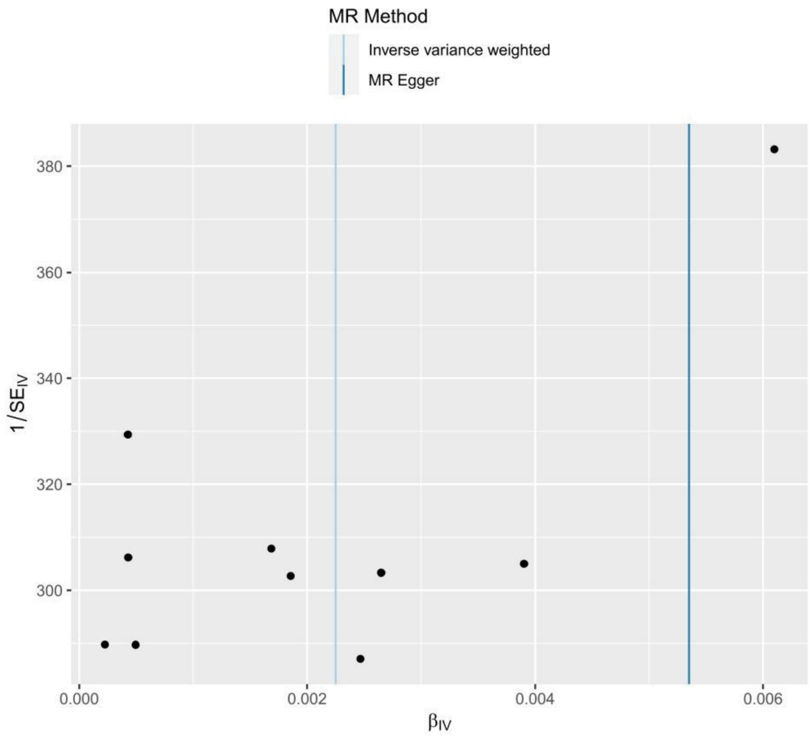

Escherichia.Shigella. funnel_plot of EFMR(main)

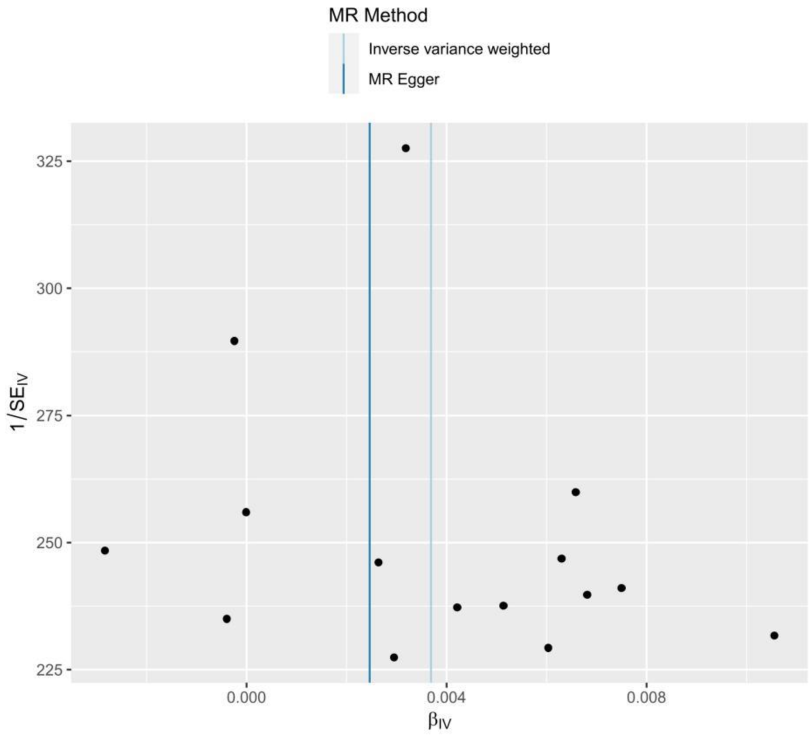

Haemophilus. funnel_plot of EFMR(main)

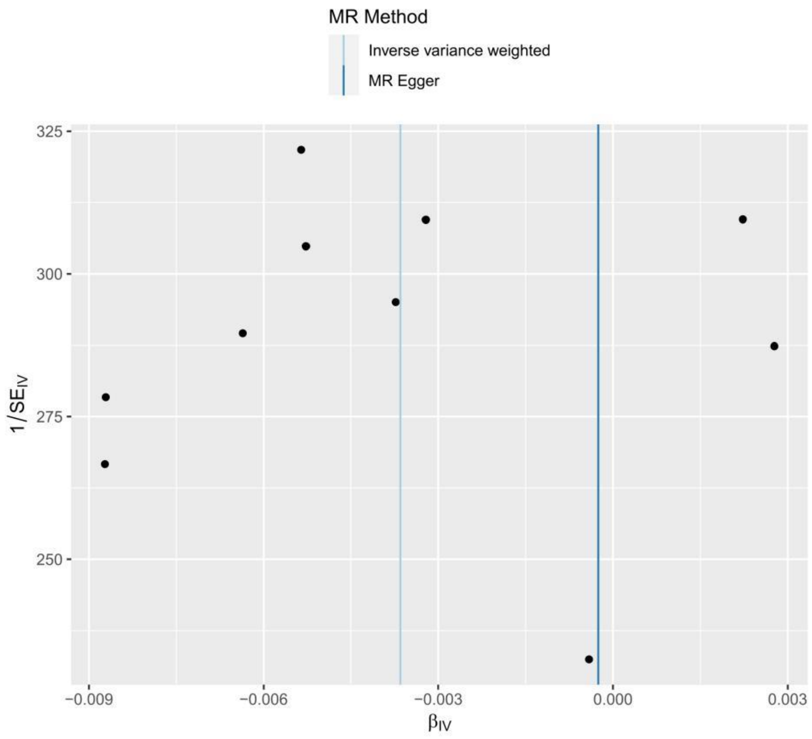

Phascolarctobacterium. funnel_plot of EFMR(main)

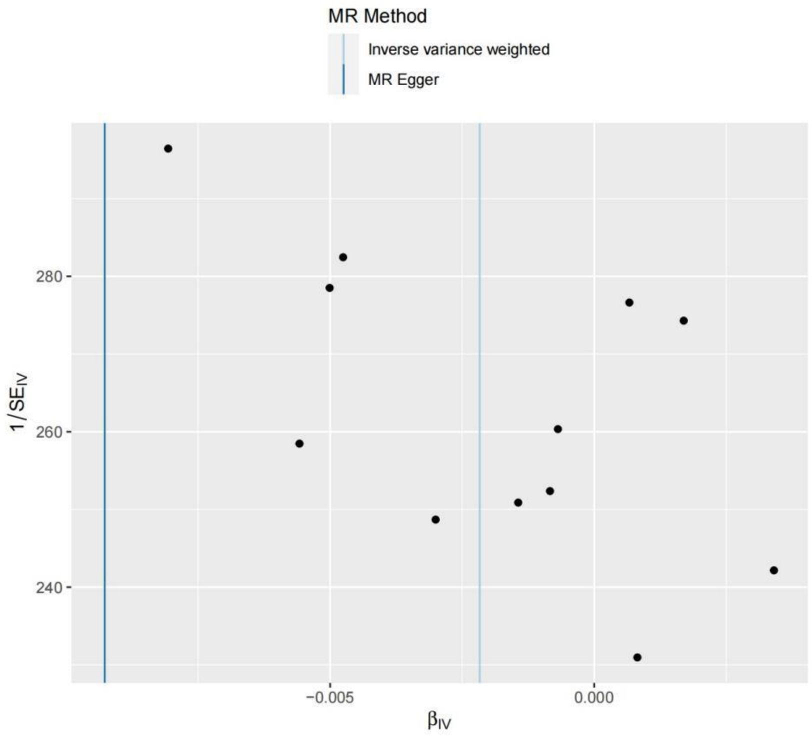

Lachnospira. funnel_plot of EFMR(main)

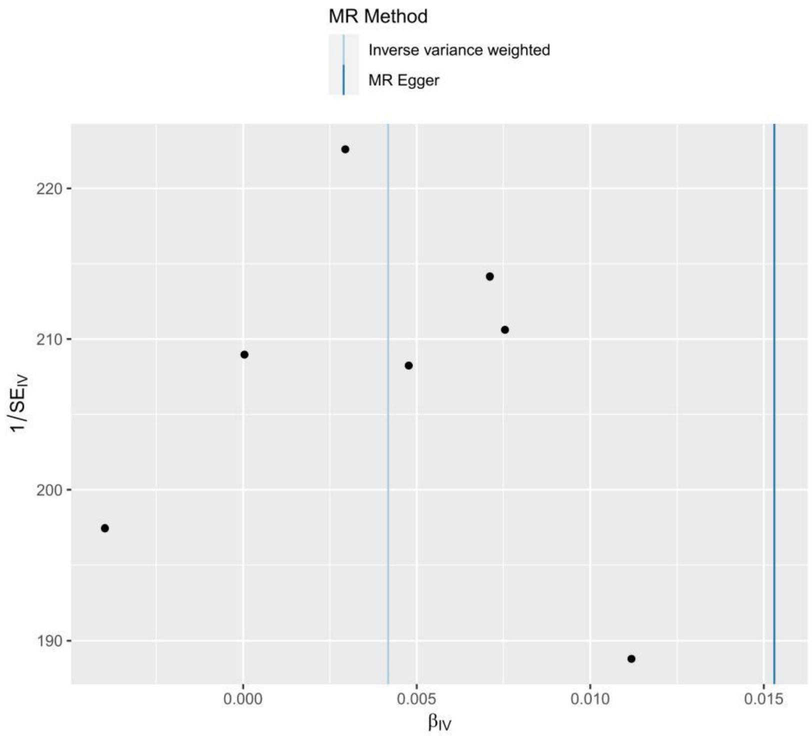

Cateni bacterium. funnel_plot of EFMR(main)

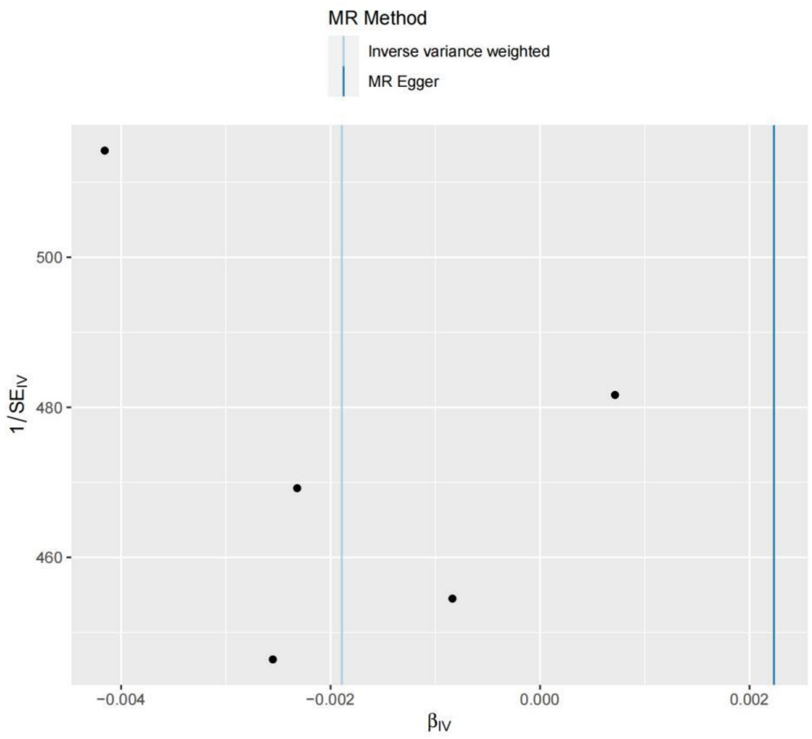

Anaerotruncus. funnel_plot of EFMR(main)

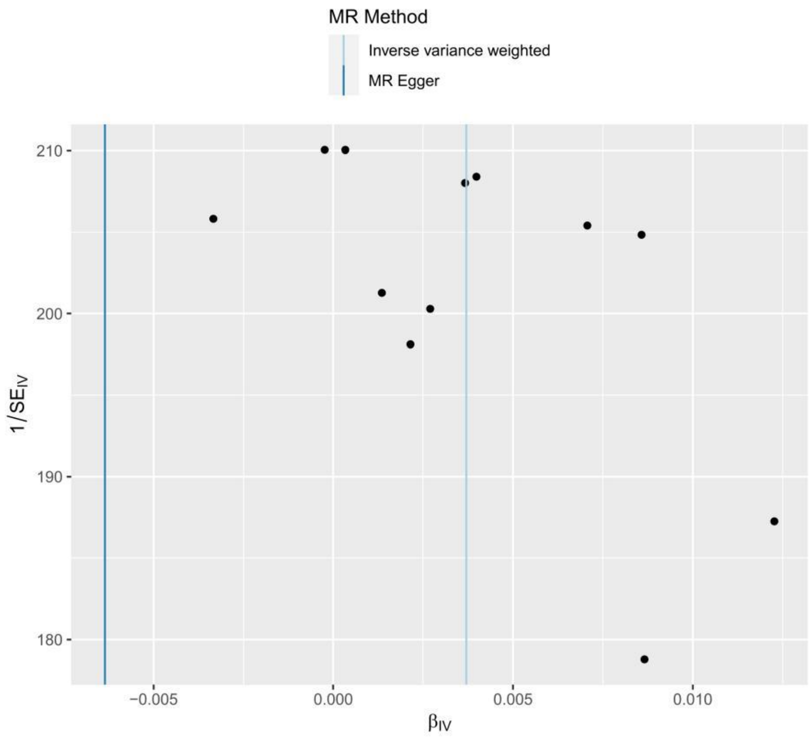

Blautia. funnel_plot of EFMR(main)

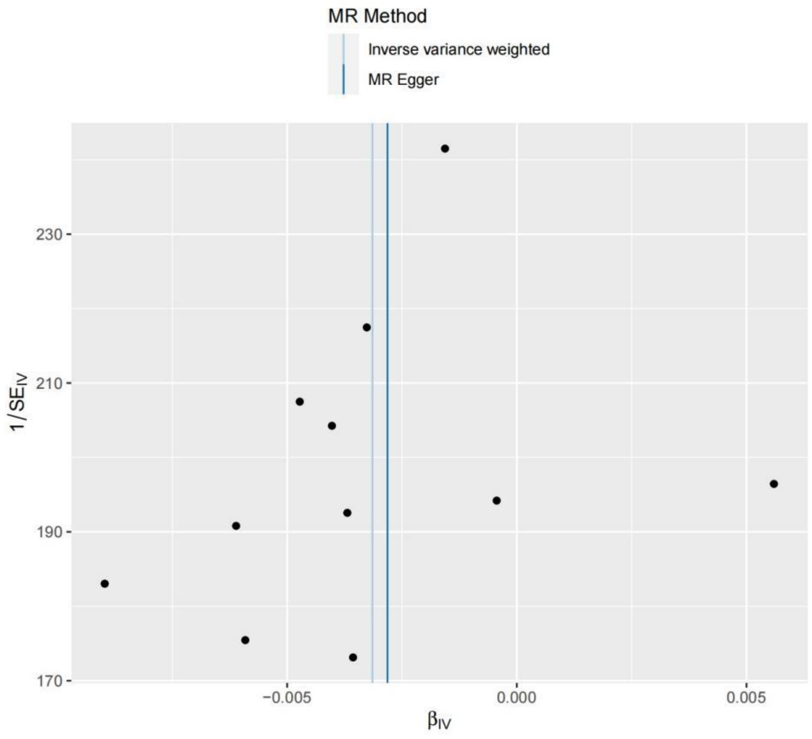

Marvinbryantia. funnel_plot of EFMR(main)

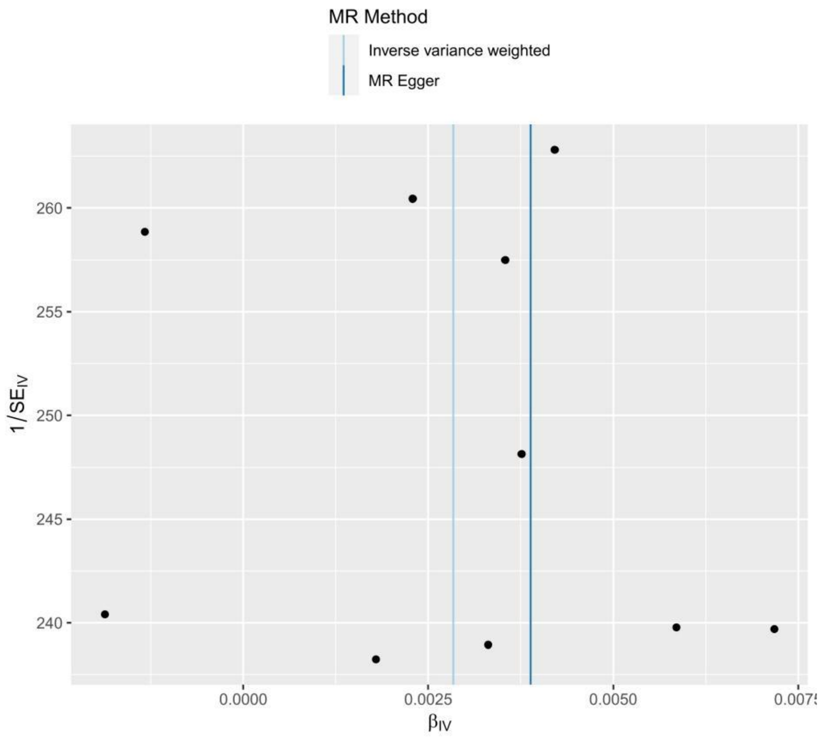

Ruminiclostridium5. funnel_plot of EFMR(secondary)

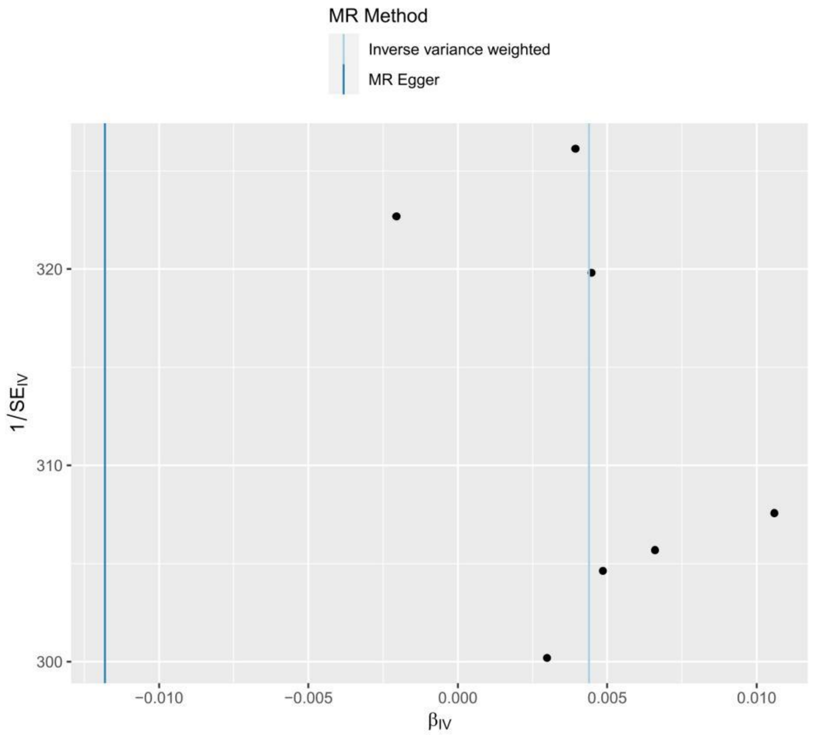

Prevotella9. funnel_plot of EFMR(secondary)

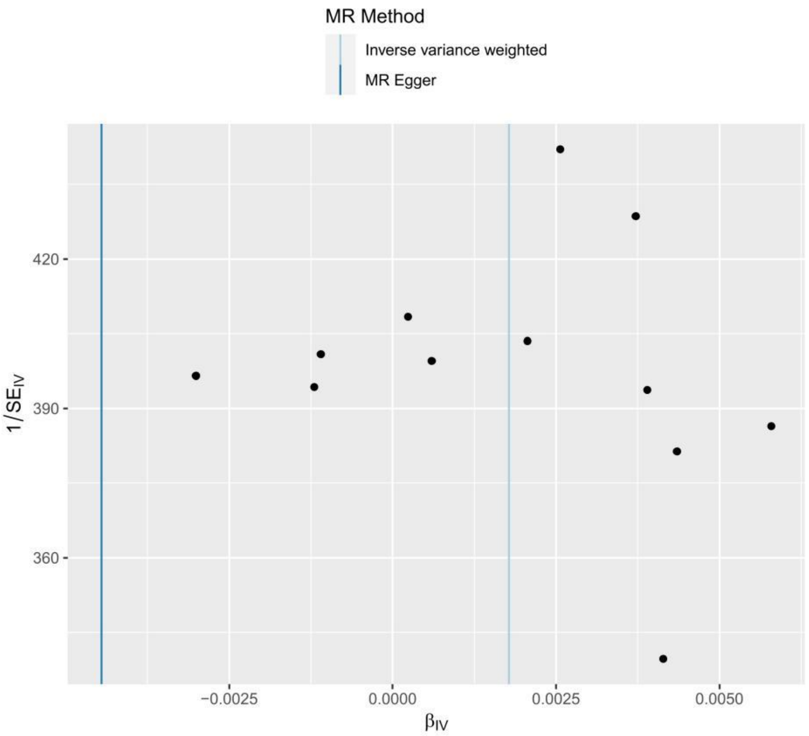

Desulfovibrio. funnel_plot of EFMR(secondary)

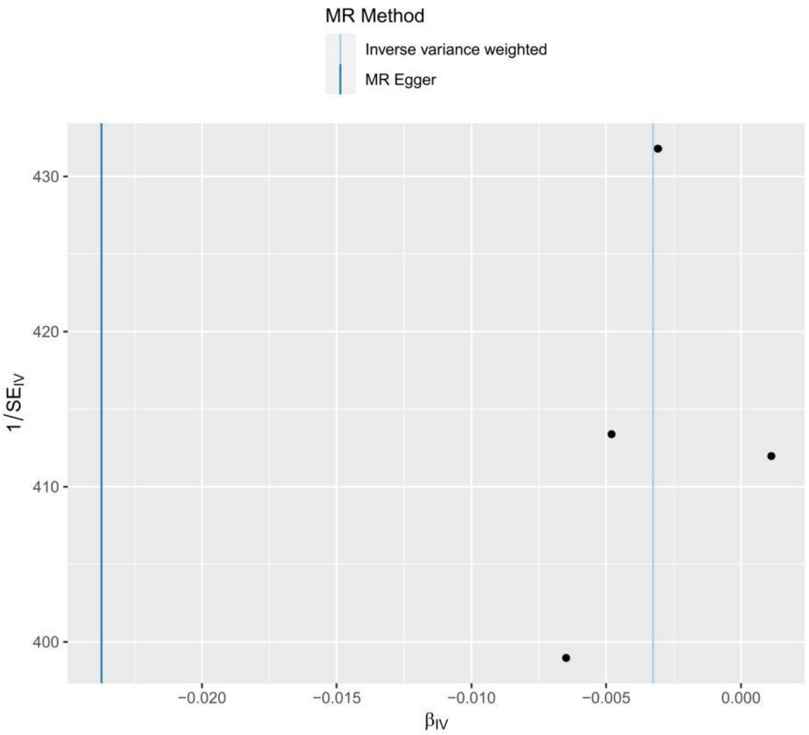

Erysipelatoclostridium. funnel_plot of EFMR(secondary)

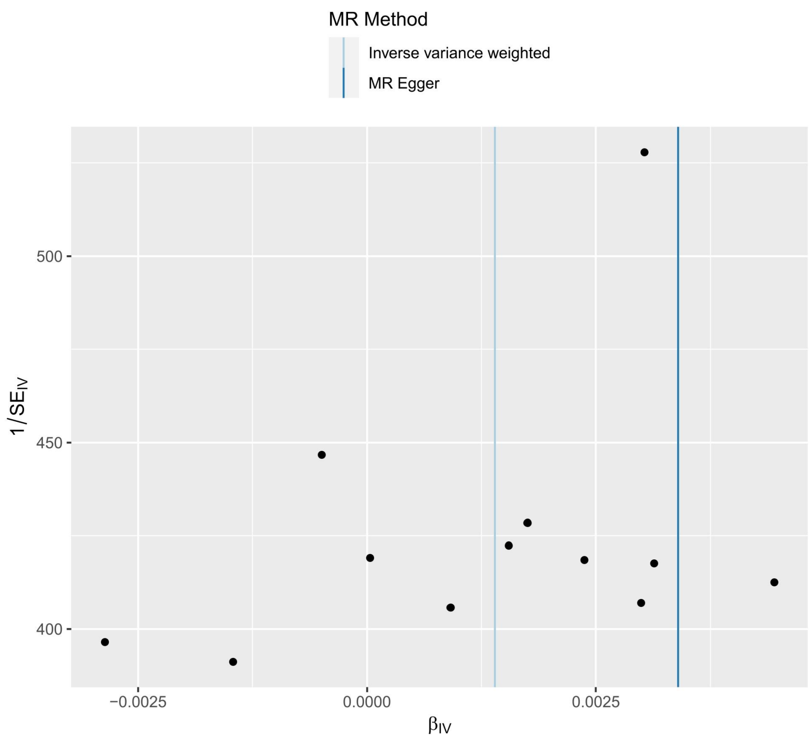

RuminococcaceaeUCG004. funnel_plot of EFMR(secondary)

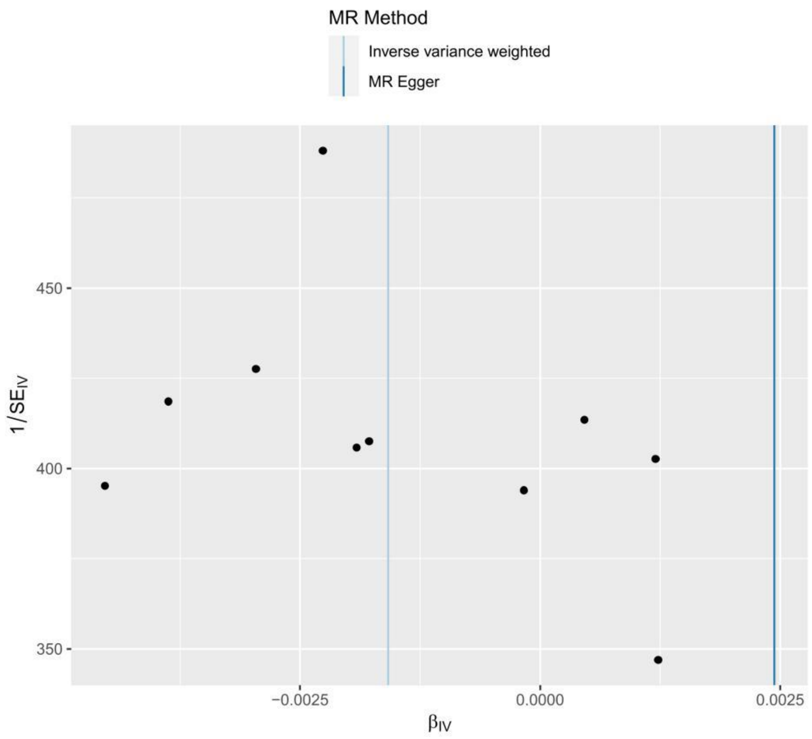

Eubacterium fissicatena group. funnel_plot of EFMR(secondary)

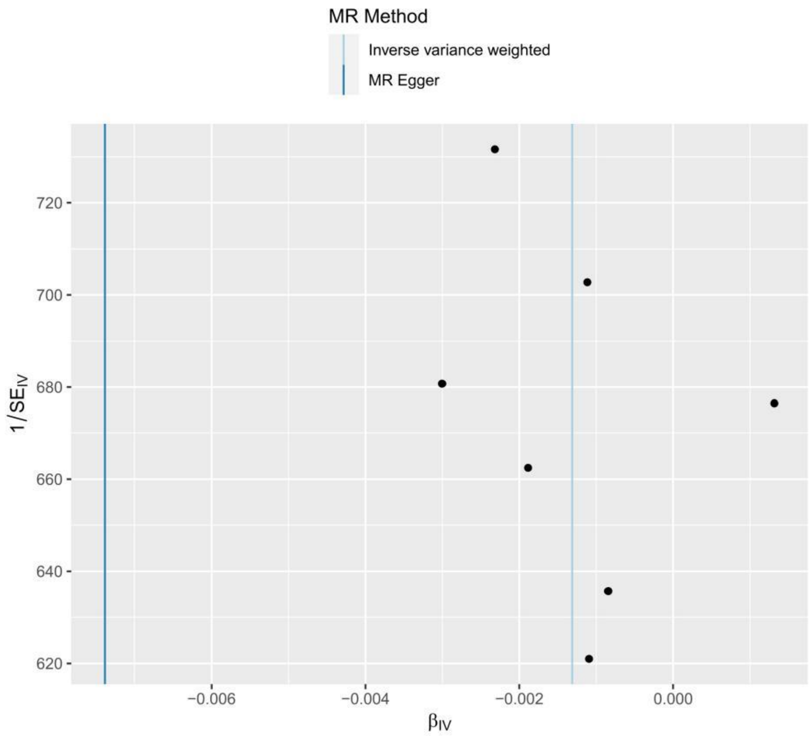

Eubacterium eligens group. funnel_plot of EFIM

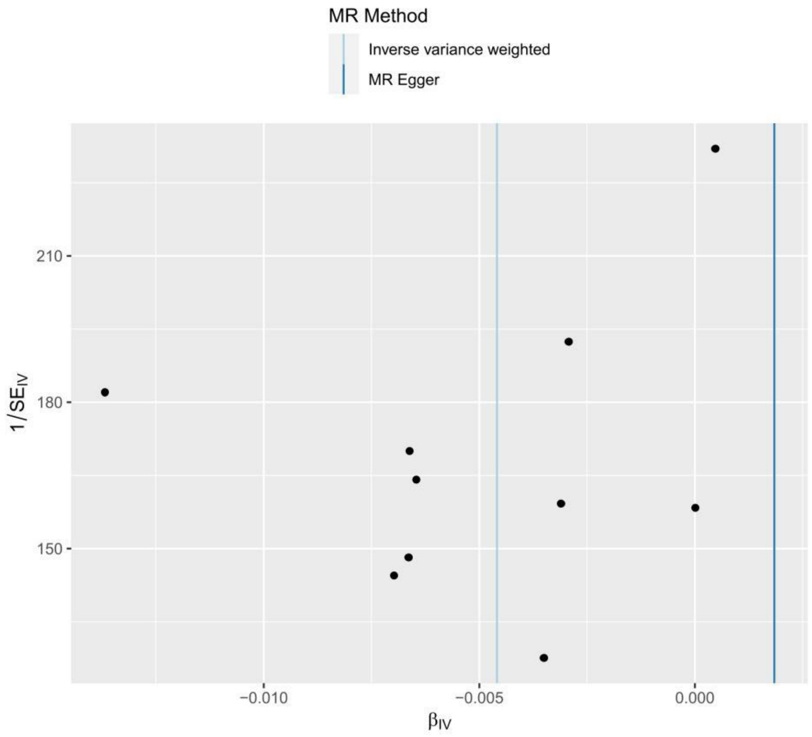

Eubacterium brachy group. funnel_plot of EFIM

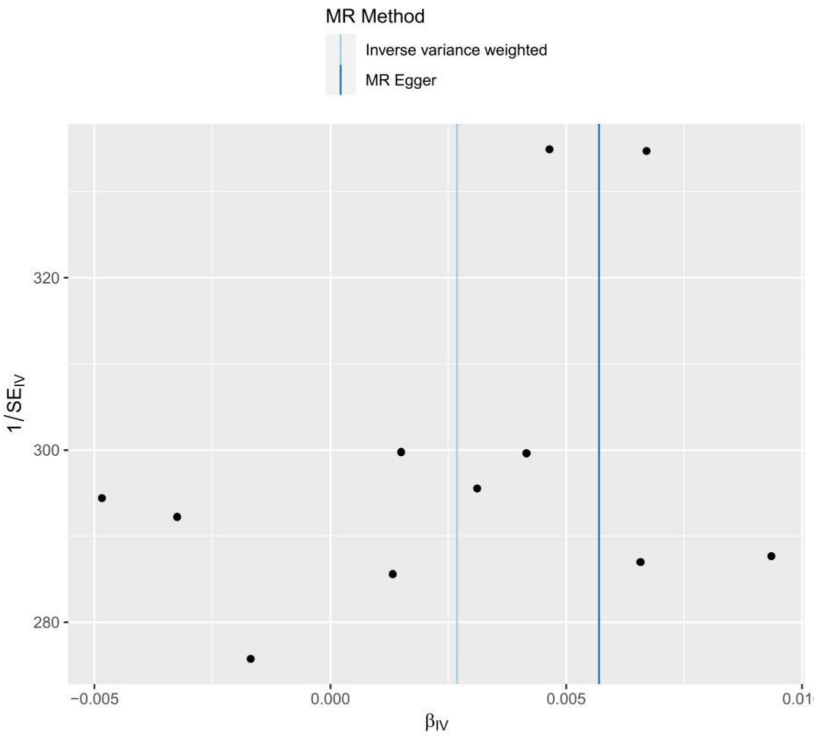

Veillonella. funnel_plot of EFIM

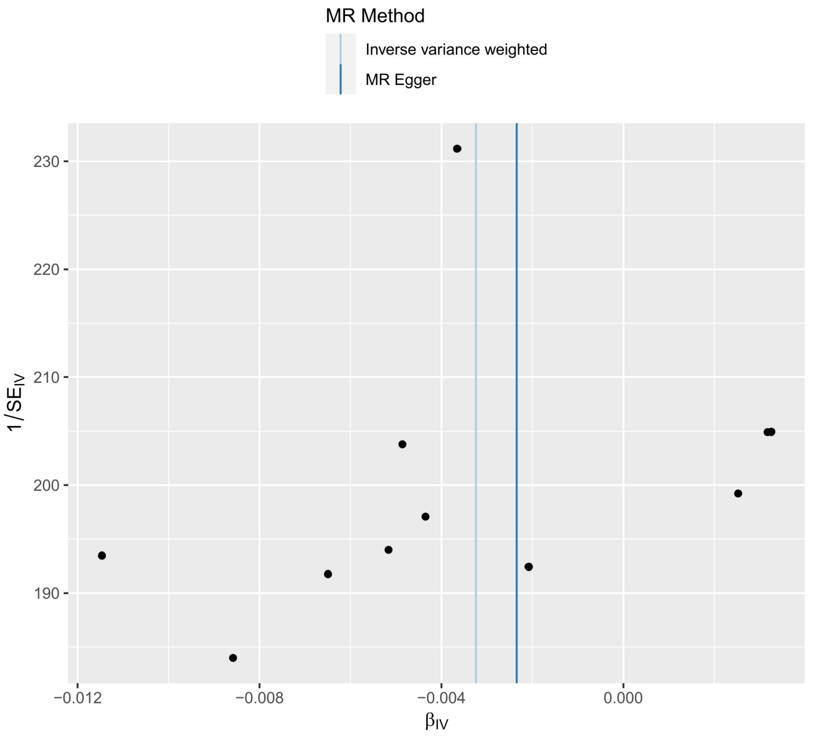

Enterorhabdus. funnel_plot of EFIM

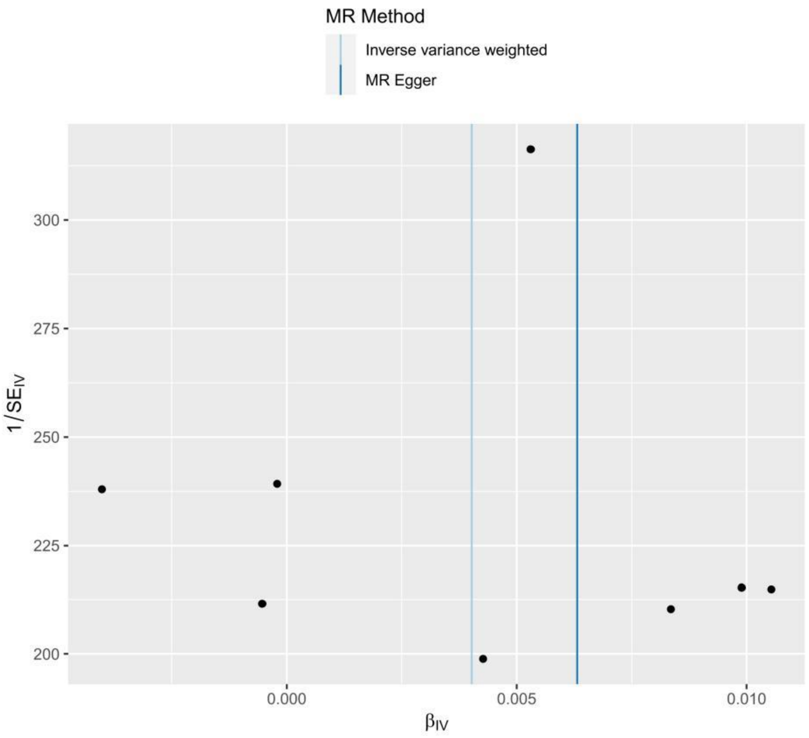

Lactococcus. funnel_plot of EFIM

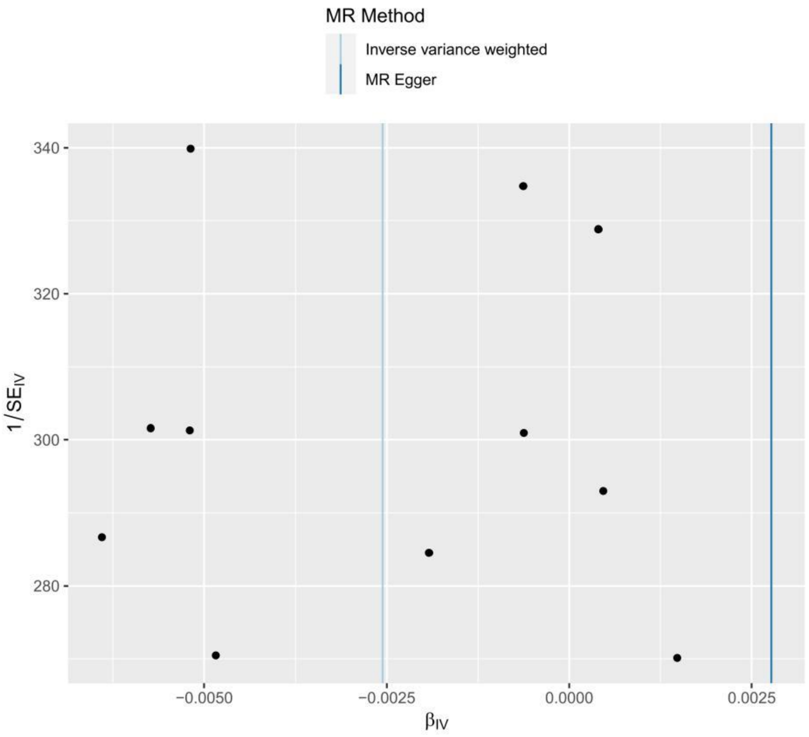

Blautia. funnel_plot of EFIM

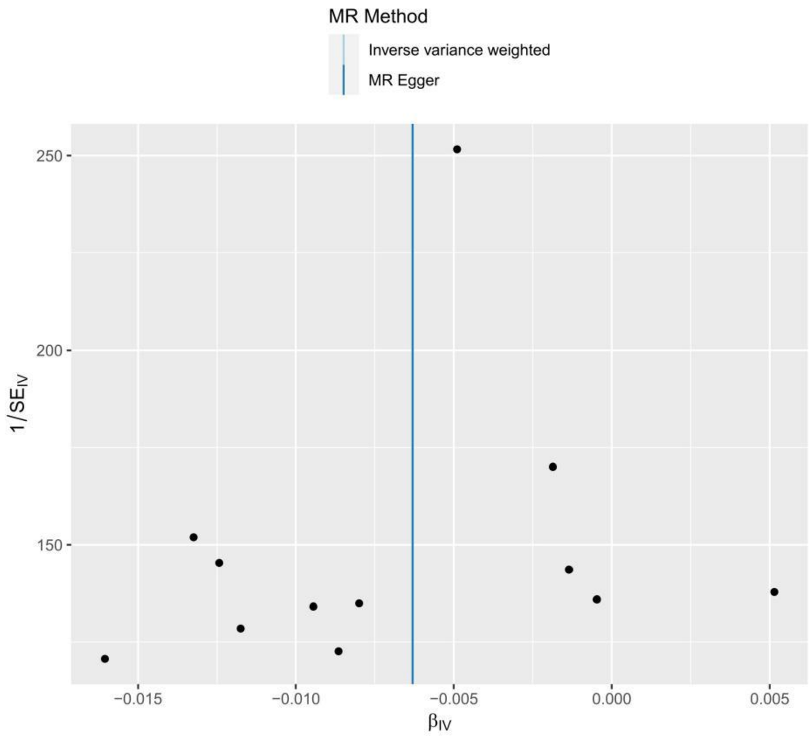

LachnospiraceaeUCG004. funnel_plot of IM(unspecified)

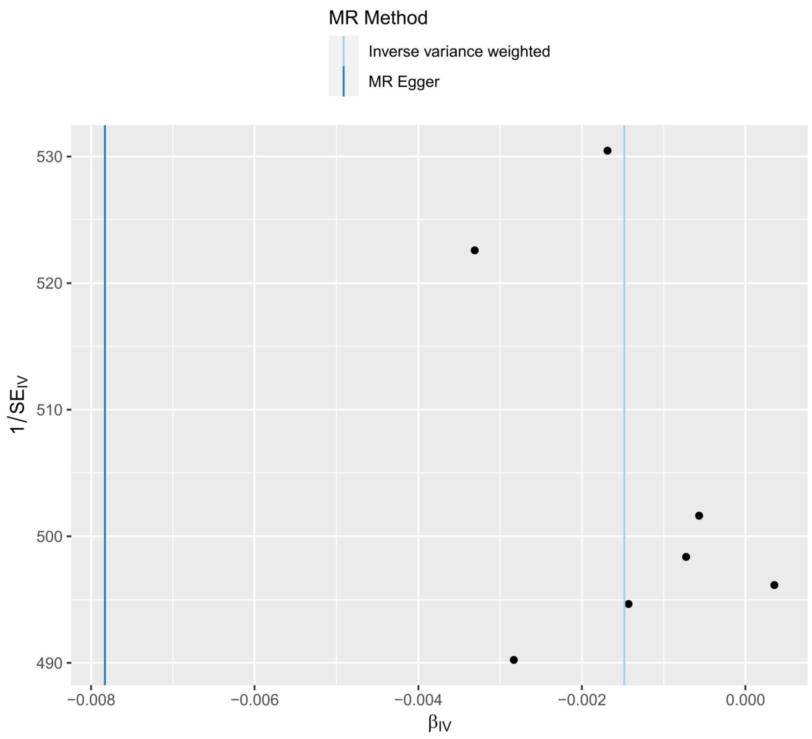

Dialister. funnel_plot of IM(unspecified)

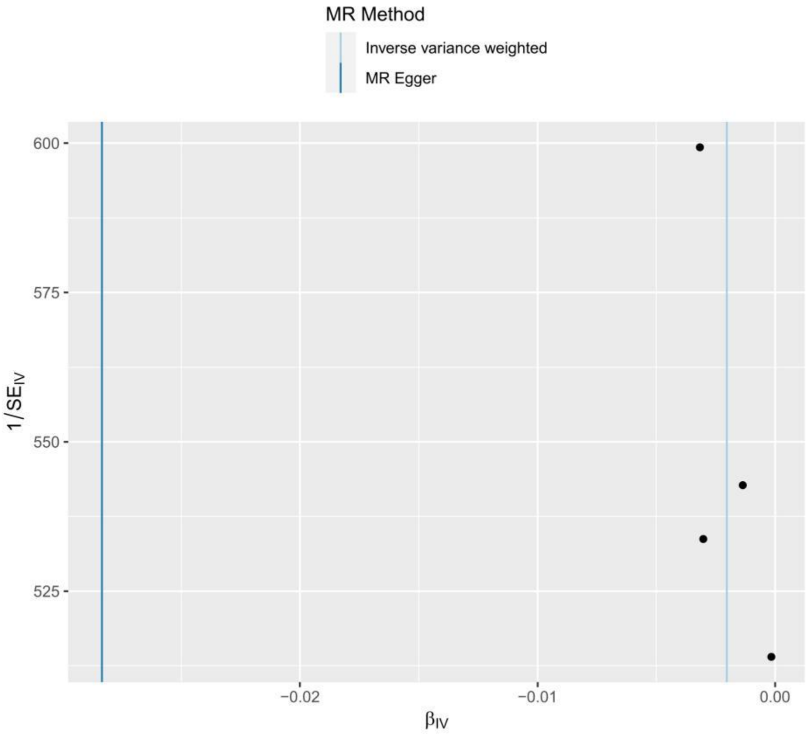

Supplement: Supplementary Figure S2 — Funnel plots of EFMR (main), EFMR (secondary), EFIM, IM(unspecified) [file Data_Sheet_2.PDF]
