# Supplementary material for: Association between gut microbiota and menstrual disorders: a two-sample Mendelian randomization study
Source: Front Microbiol. 2024 Mar 7;15:1321268. doi: 10.3389/fmicb.2024.1321268 (PMC10954809; doi:10.3389/fmicb.2024.1321268)

Eubacterium eligens group.leave-one-out of EFMR(main)

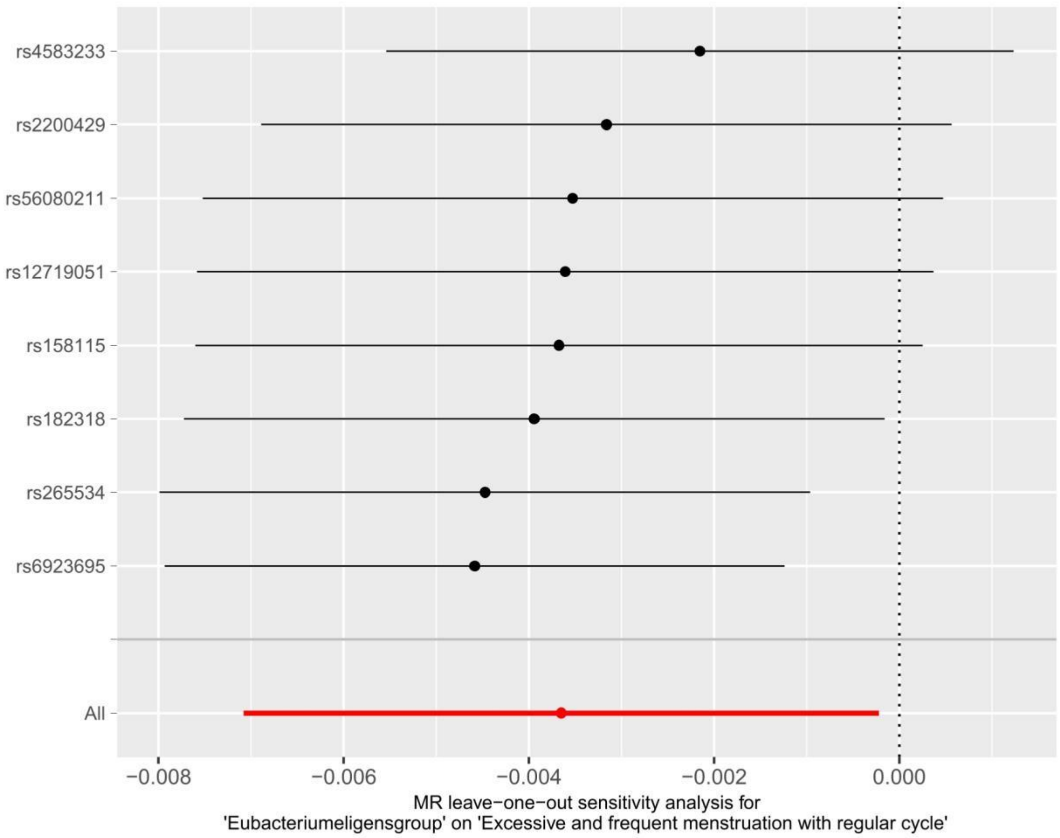

RuminococcaceaeUCG011.leave-one-out of EFMR(main)

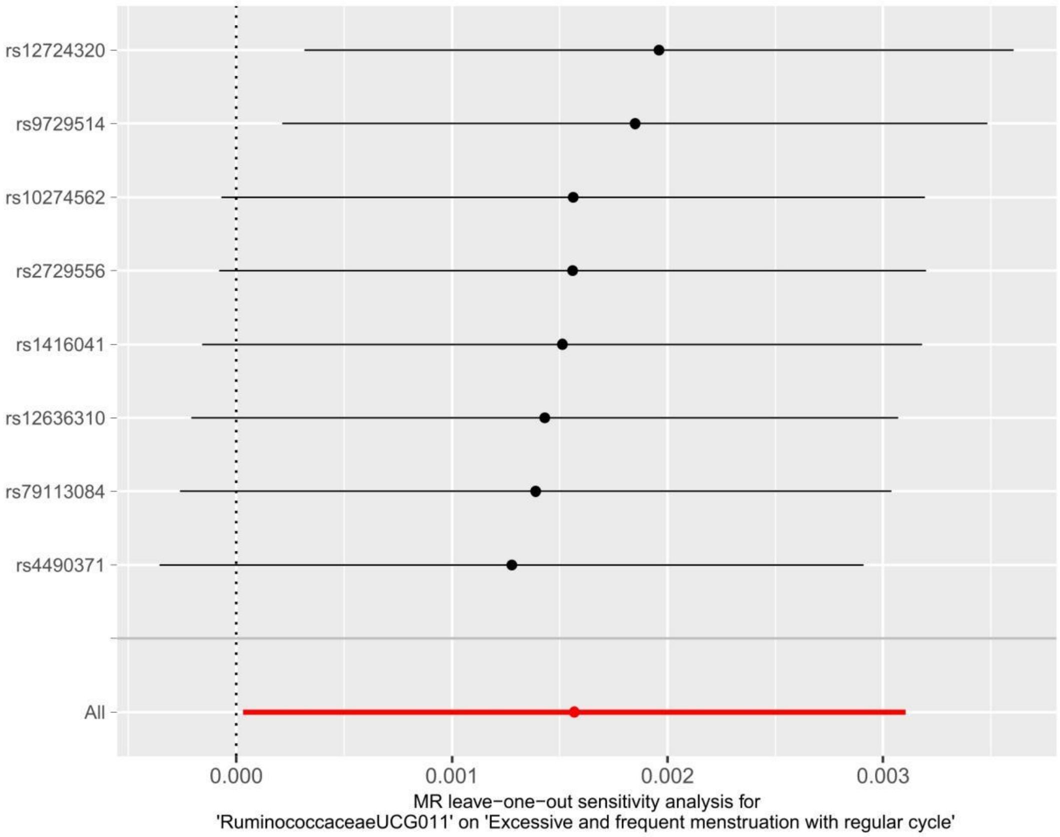

DefluviitaleaceaeUCG011.leave-one-out of EFMR(main)

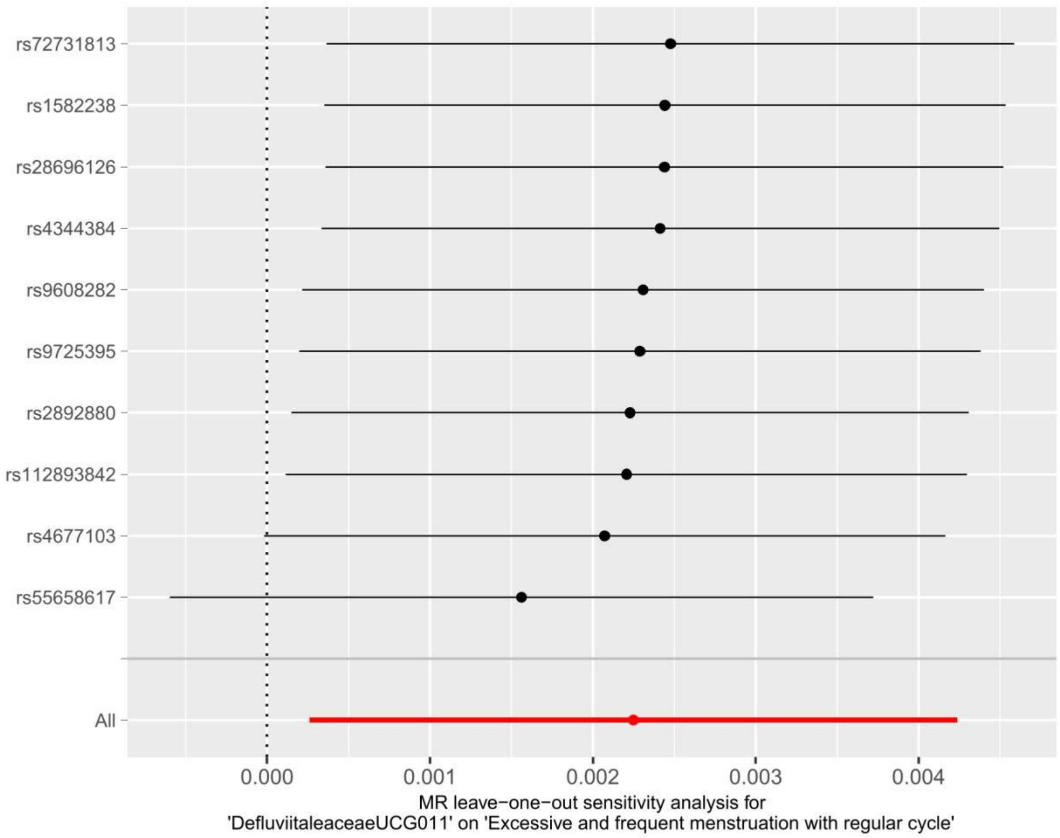

Escherichia.Shigella.leave-one-out of EFMR(main)

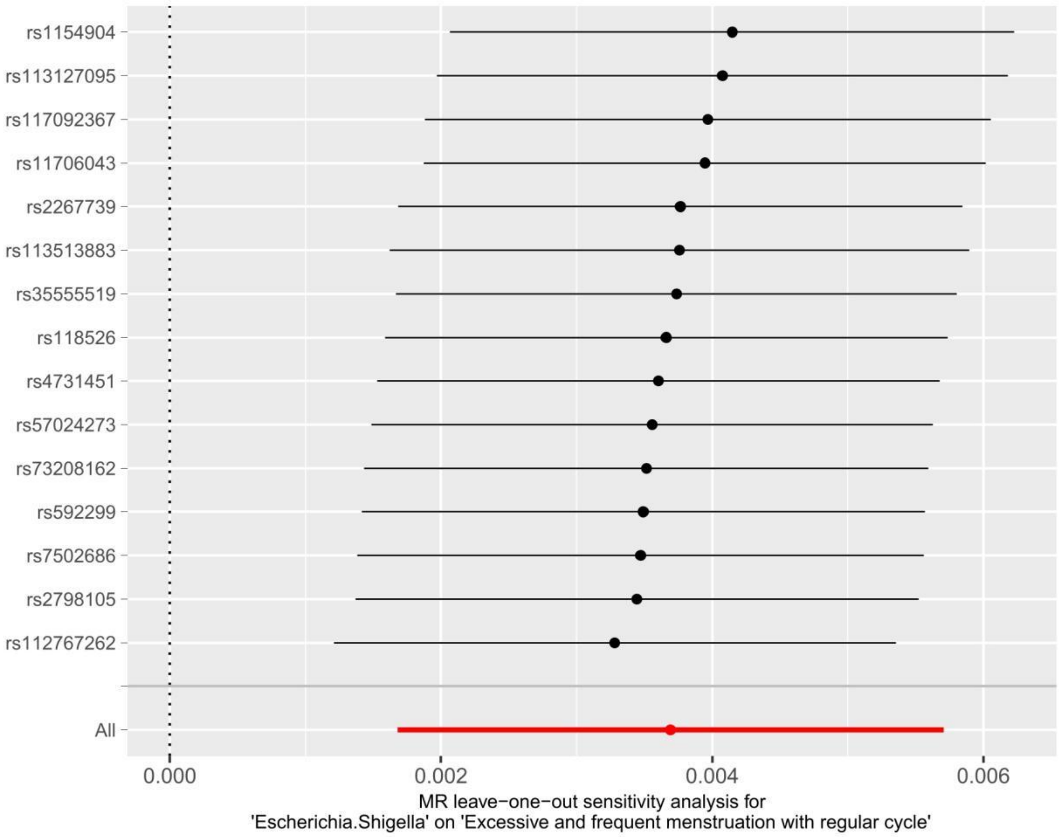

Haemophilus.leave-one-out of EFMR(main)

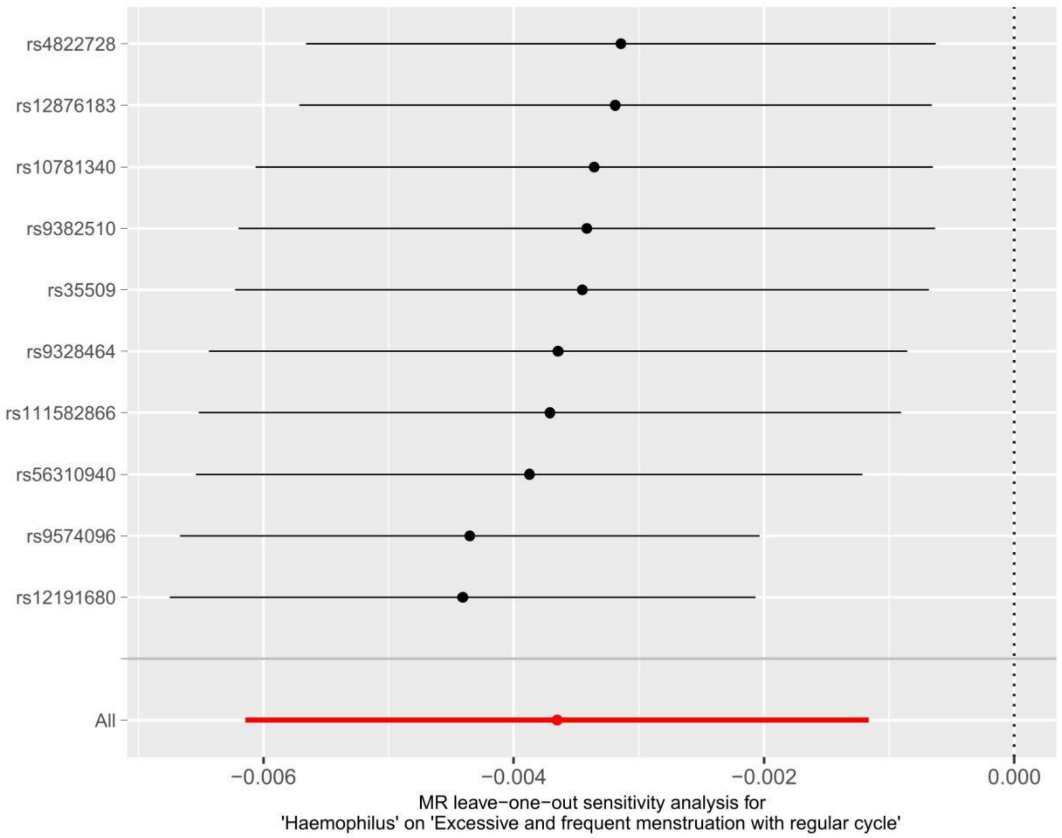

Phascolarctobacterium.leave-one-out of EFMR(main)

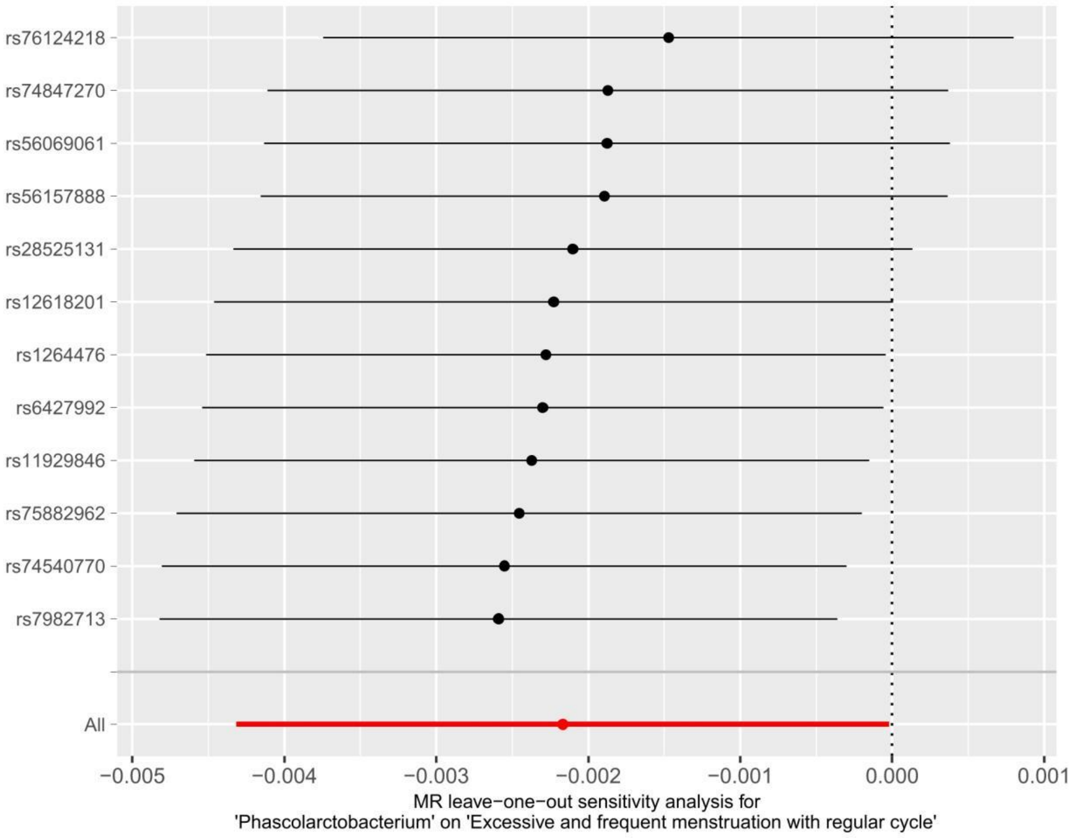

Lachnospira.leave-one-out of EFMR(main)

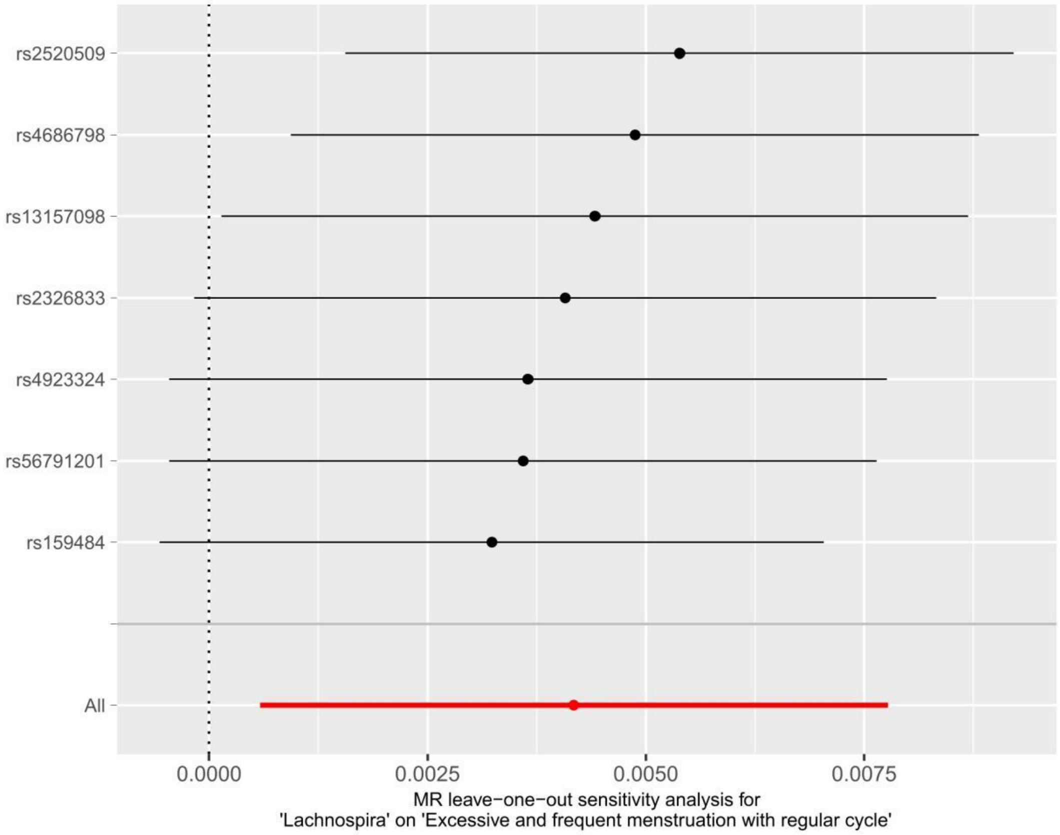

Catenibacterium.leave-one-out of EFMR(main)

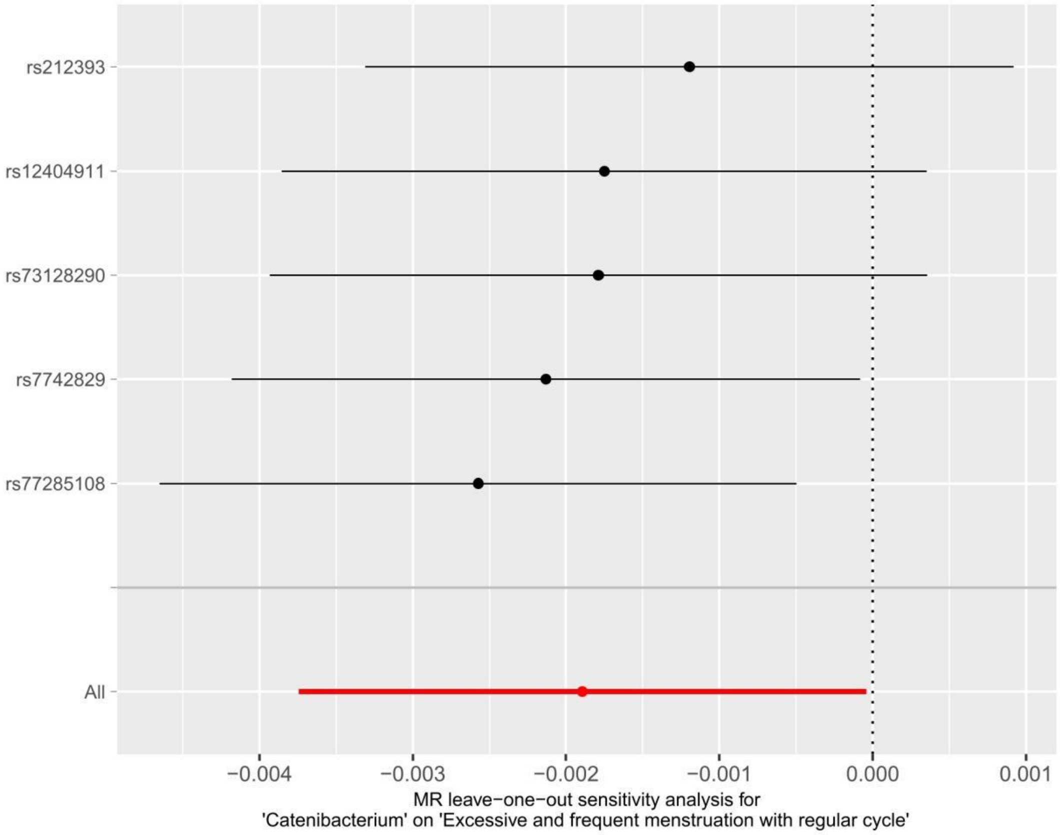

Anaerotruncus.leave-one-out of EFMR(main)

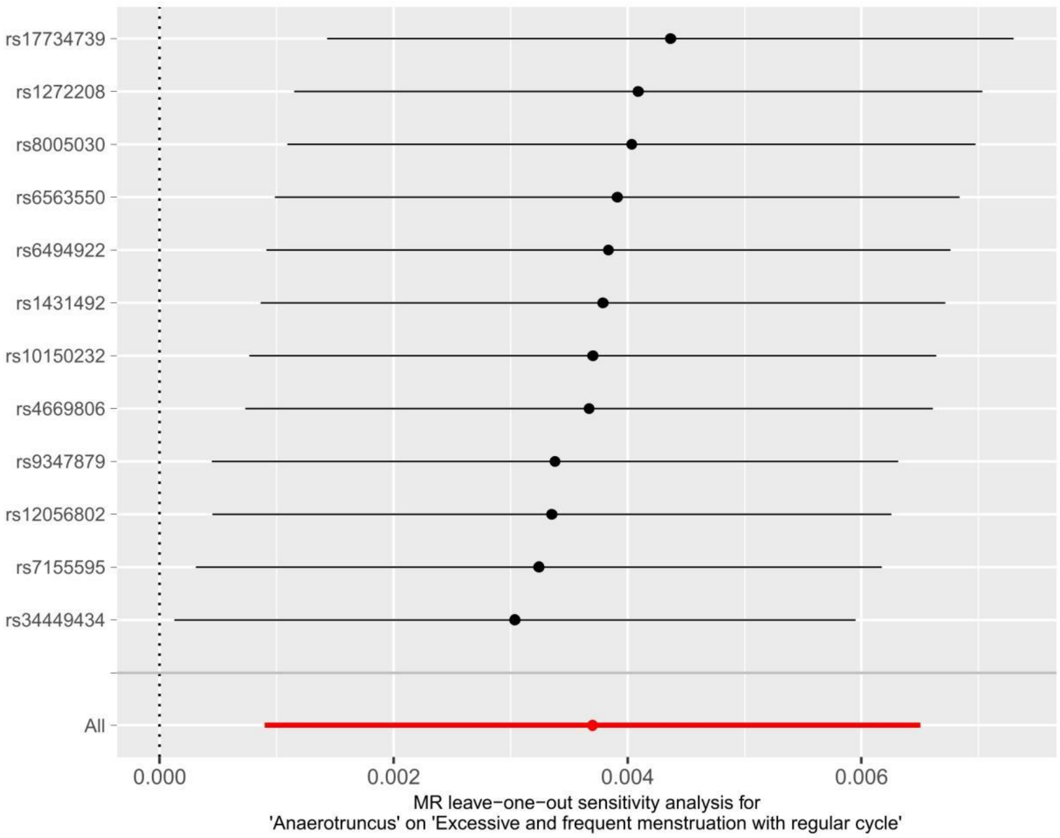

Blautia.leave-one-out of EFMR(main)

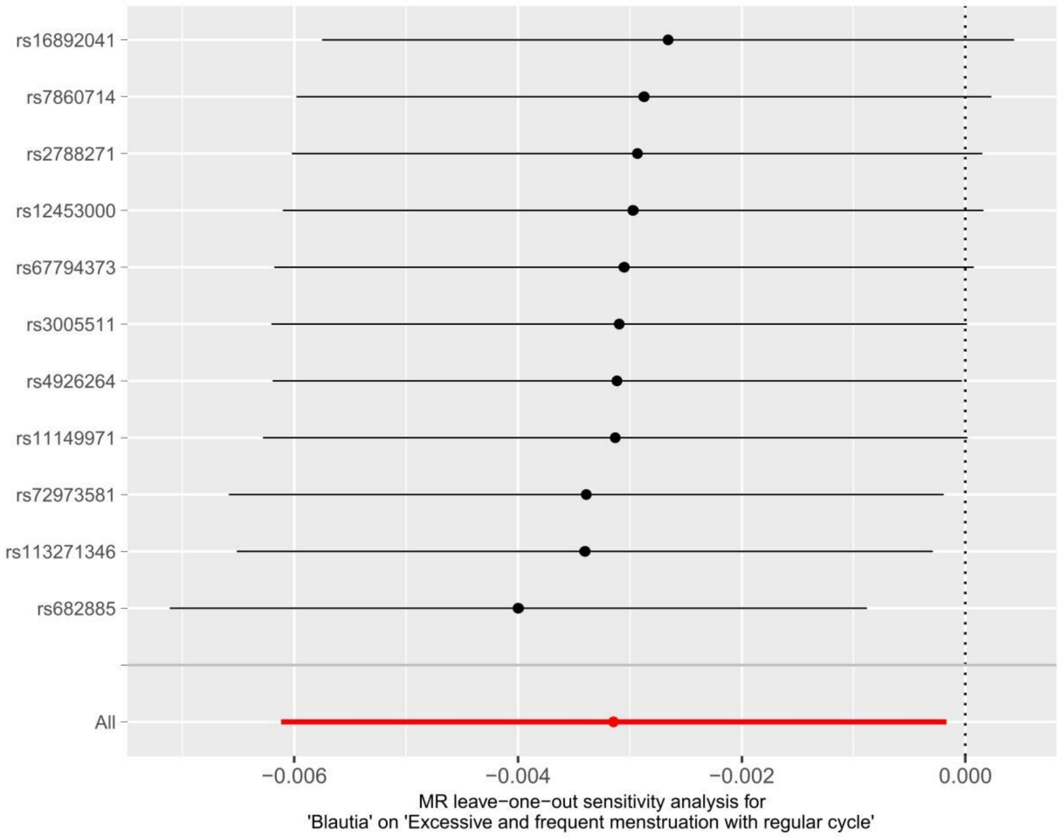

Marvinbryantia.leave-one-out of EFMR(main)

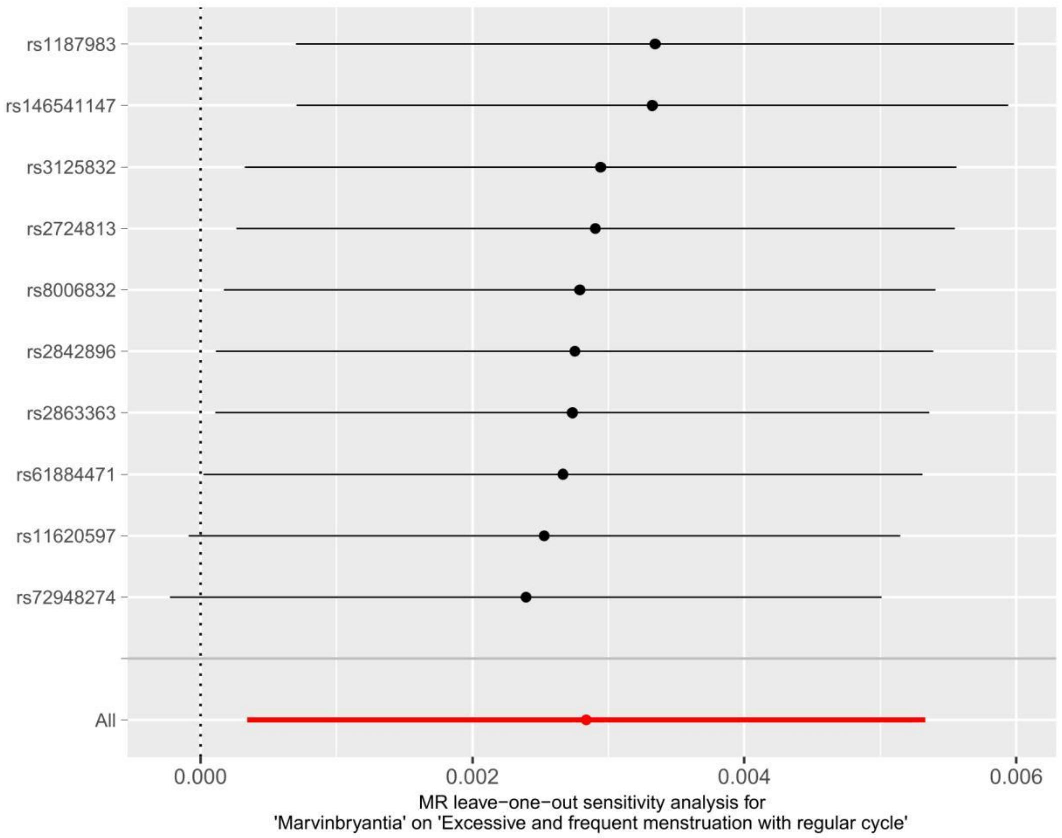

Ruminiclostridium5.leave-one-out of EFMR(secondary)

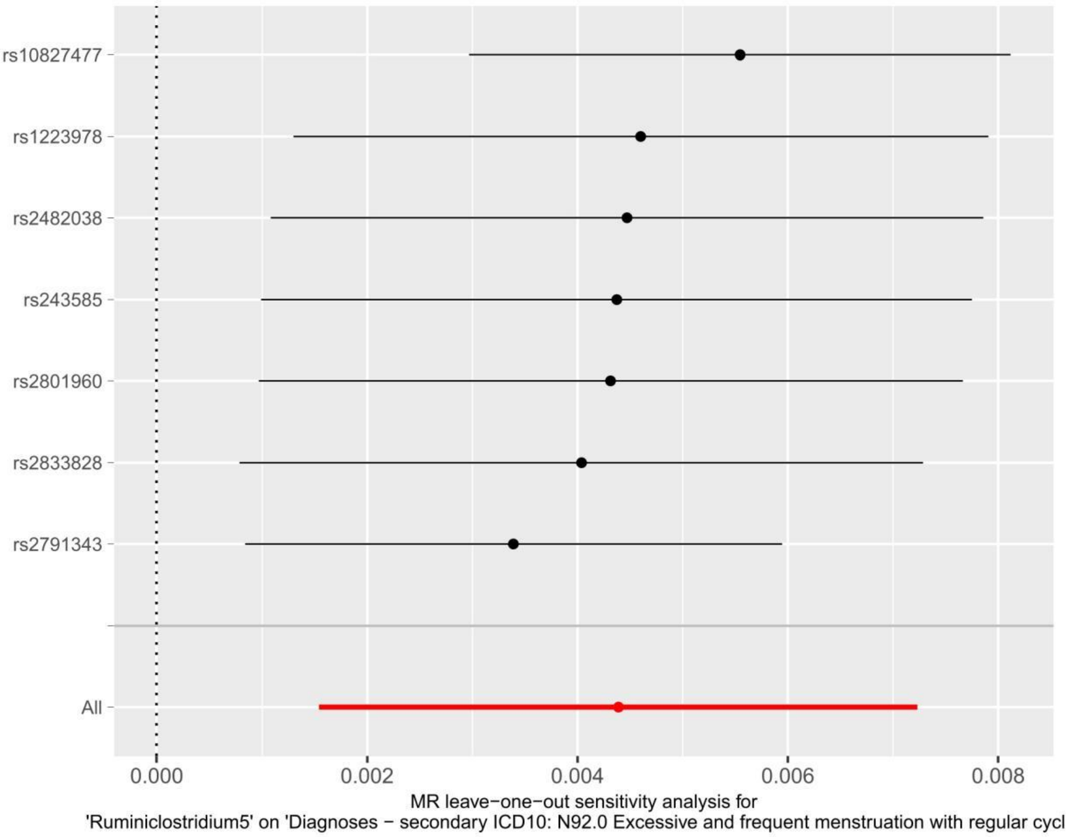

Prevotella9.leave-one-out of EFMR(secondary)

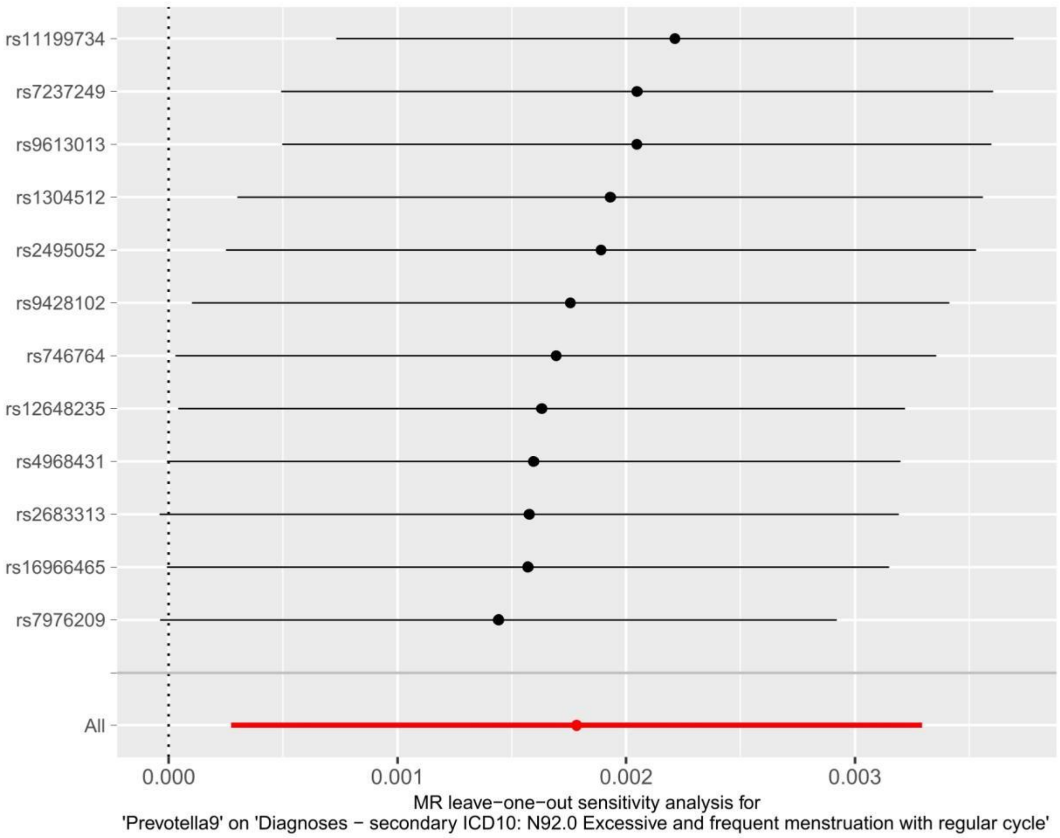

Desulfovibrio.leave-one-out of EFMR(secondary)

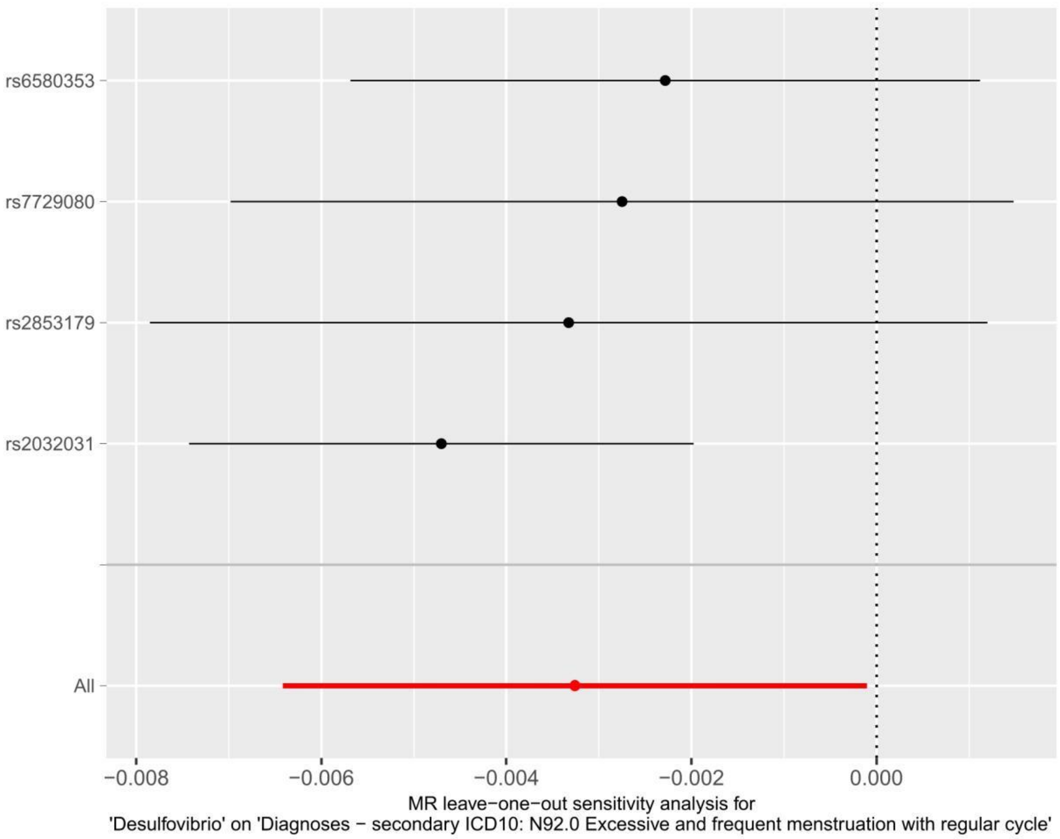

Erysipelatoclostridium.leave-one-out of EFMR(secondary)

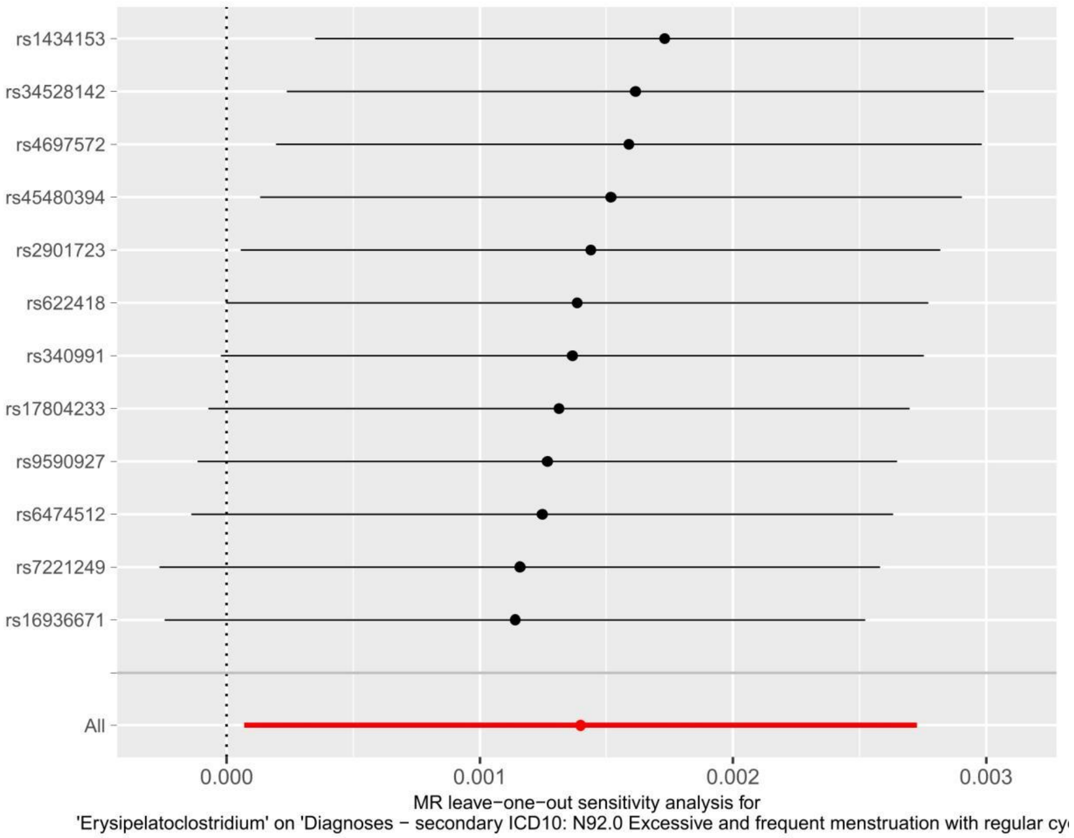

RuminococcaceaeUCG004.leave-one-out of EFMR(secondary)

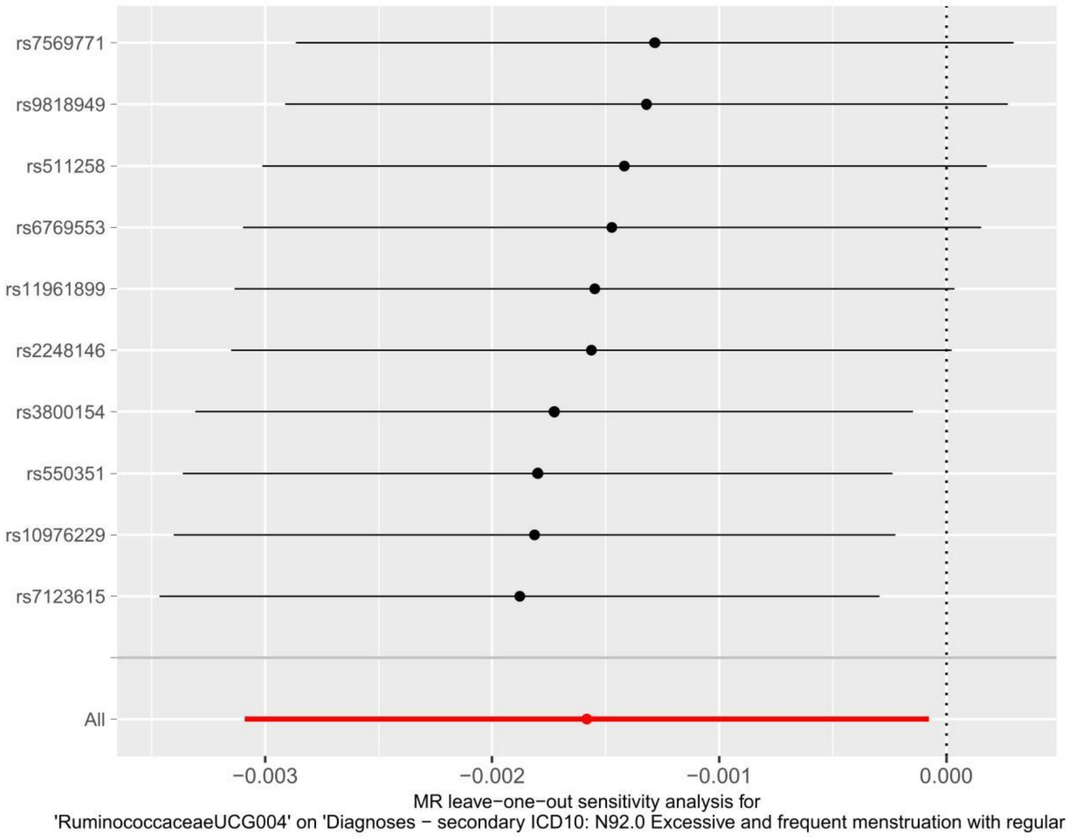

Eubacterium fissicatena group.leave-one-out of EFMR(secondary)

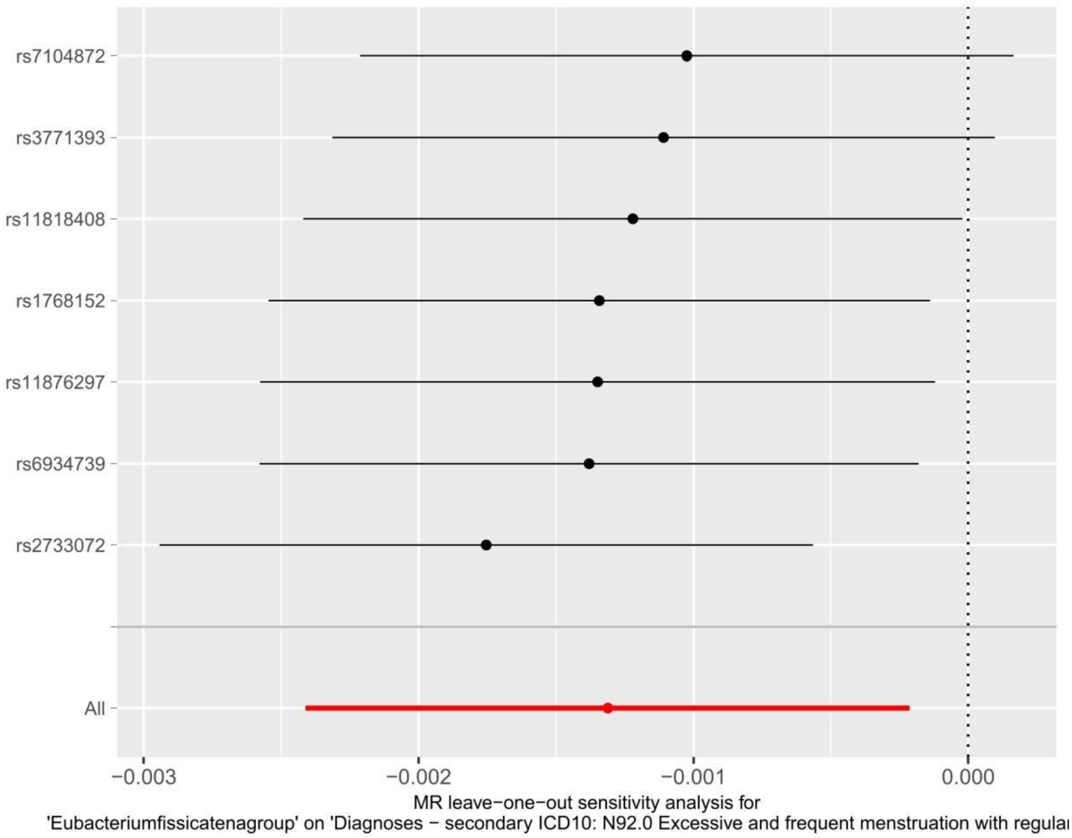

Eubacterium eligens group.leave-one-out of EFIM

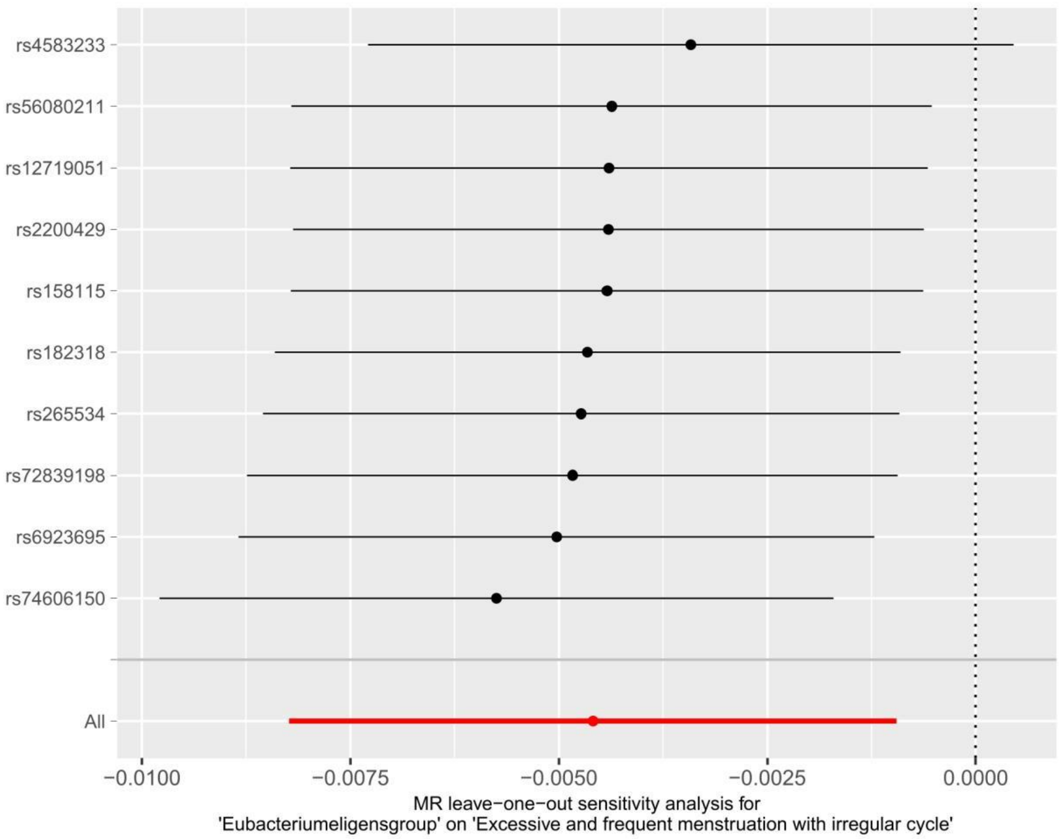

Eubacterium brachy group.leave-one-out of EFIM

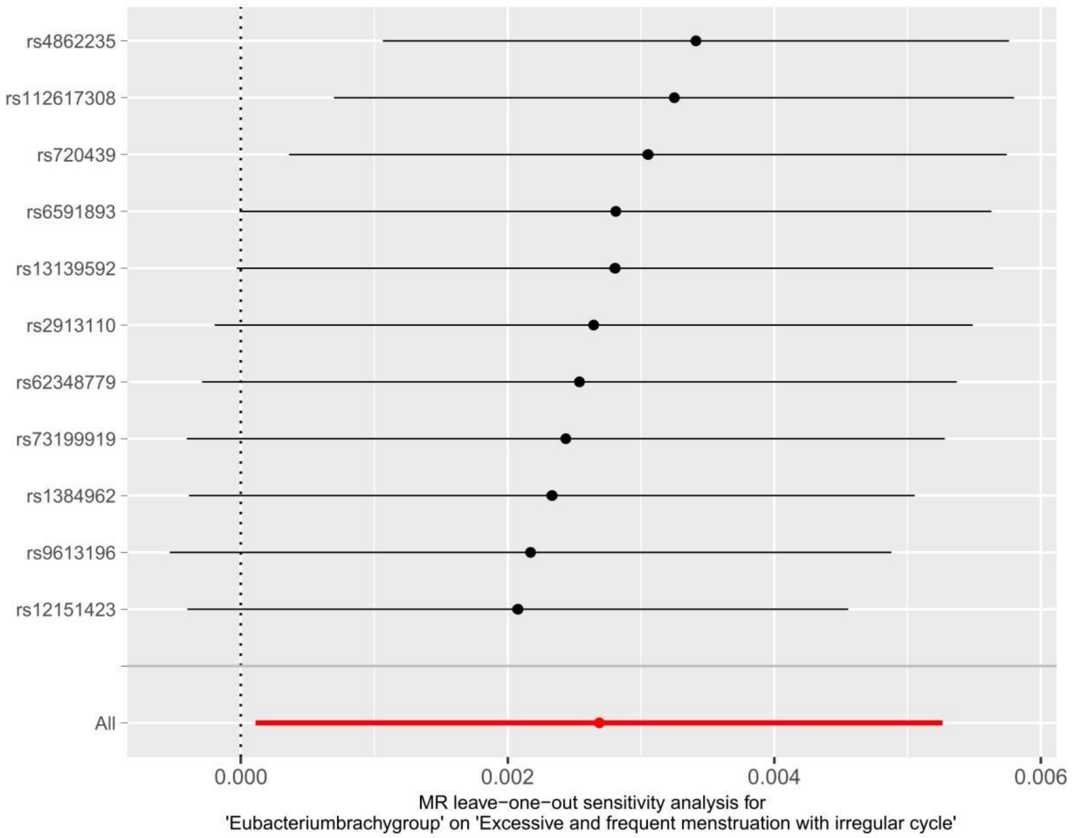

Veillonella.leave-one-out of EFIM

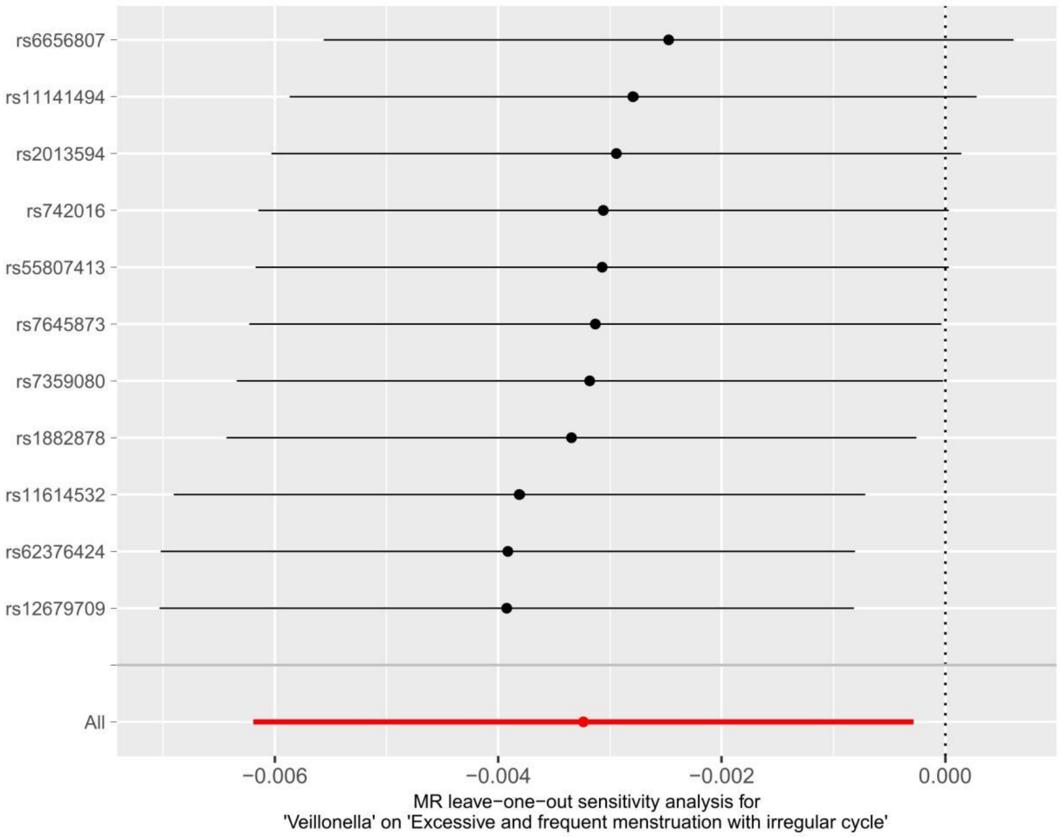

Enterorhabdus.leave-one-out of EFIM

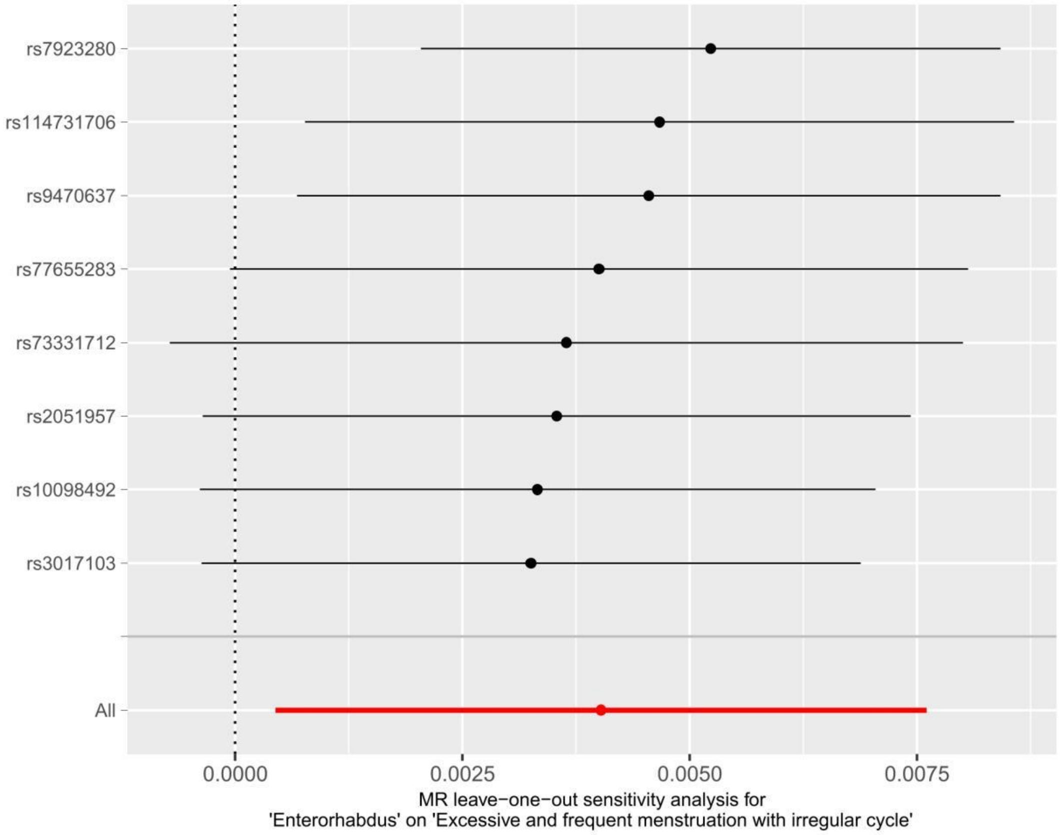

Lactococcus.leave-one-out of EFIM

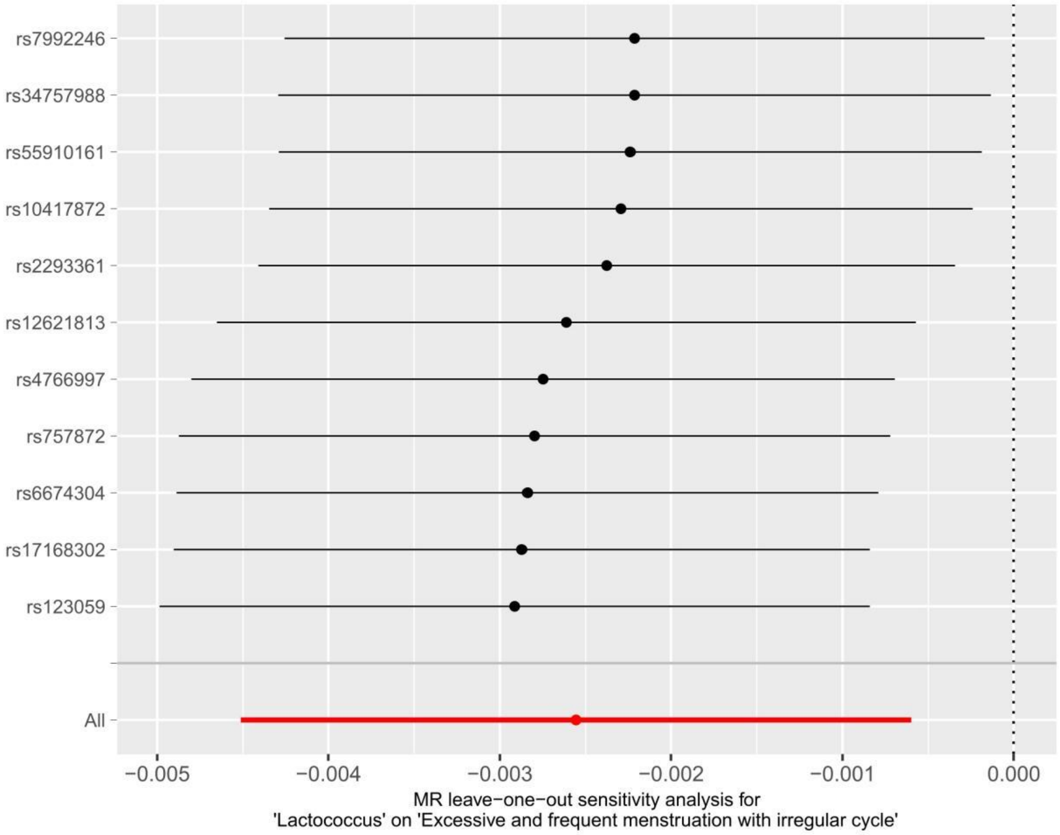

Blautia.leave-one-out of EFIM

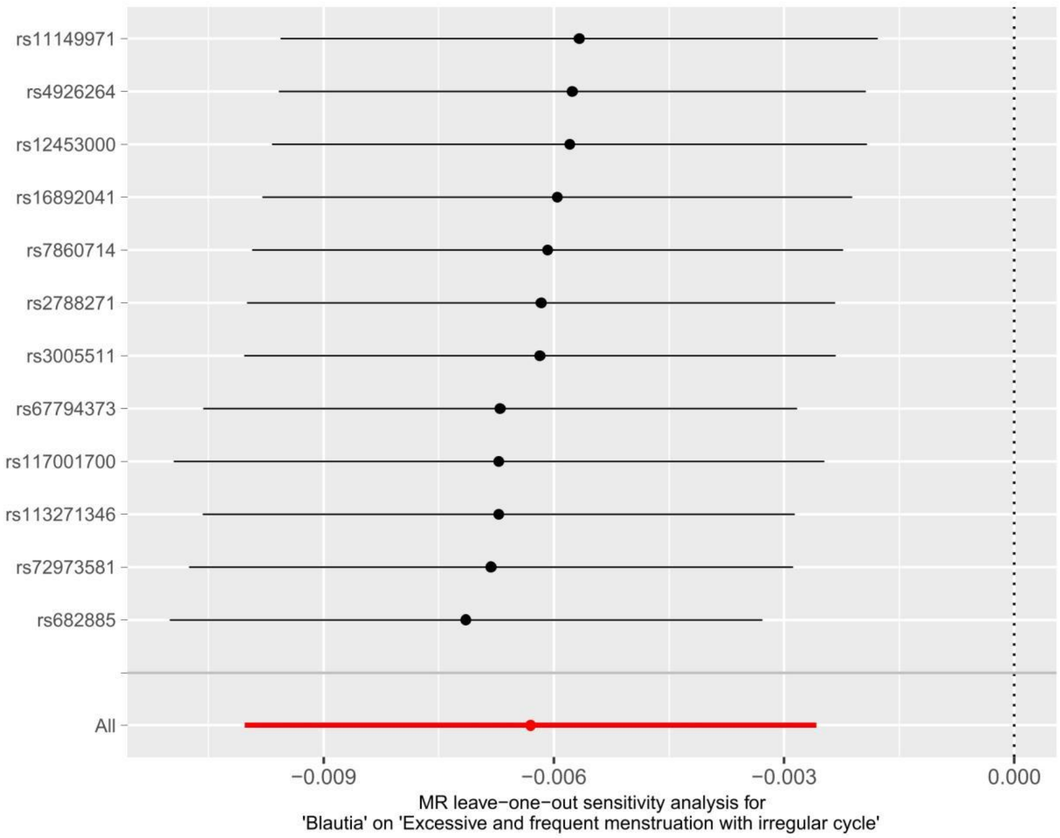

LachnospiraceaeUCG004.leave-one-out of IM

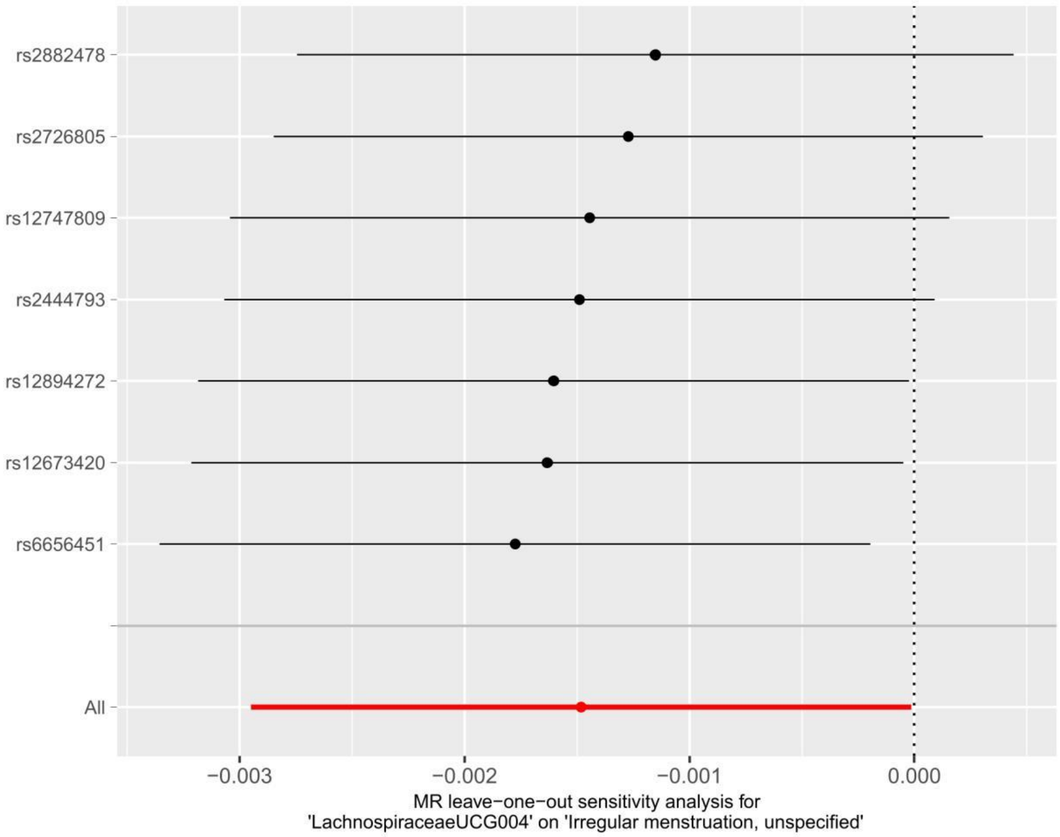

Dialister.leave-one-out of IM

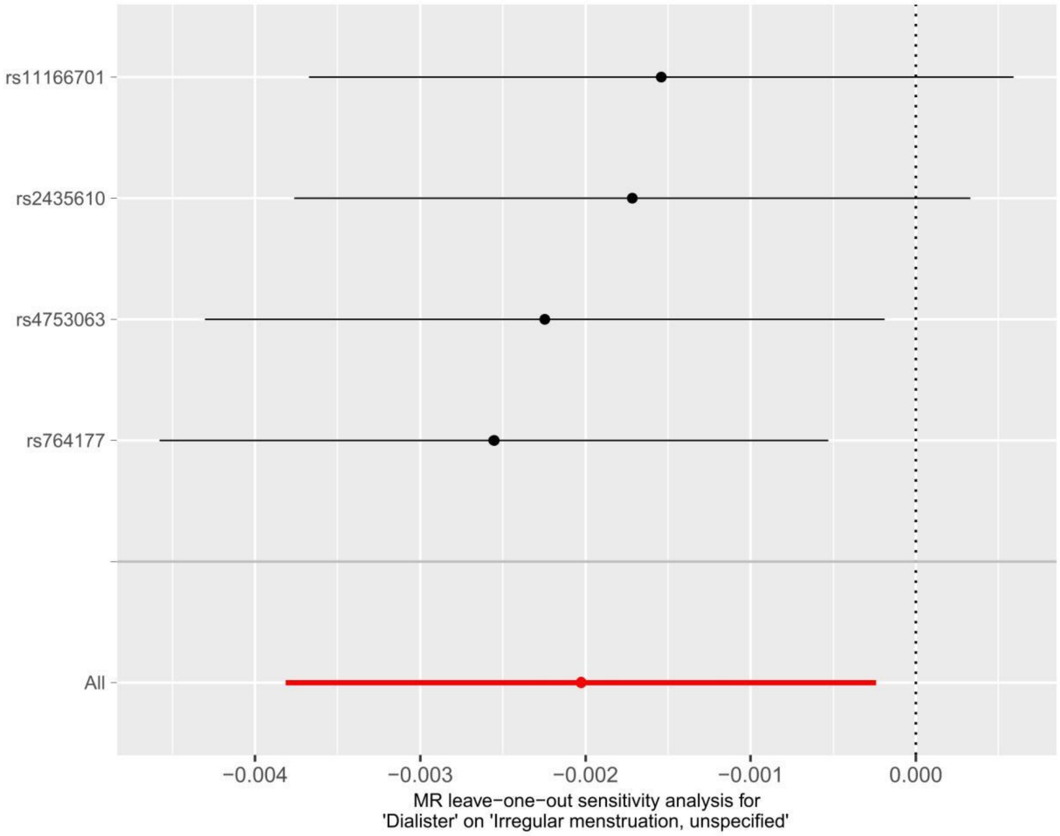

Supplement: Supplementary Figure S3 — Leave-one-out plots of EFMR (main), EFMR (secondary), EFIM, IM(unspecified). [file Data_Sheet_3.PDF]
