# Supplementary material for: Association between gut microbiota and menstrual disorders: a two-sample Mendelian randomization study
Source: Front Microbiol. 2024 Mar 7;15:1321268. doi: 10.3389/fmicb.2024.1321268 (PMC10954809; doi:10.3389/fmicb.2024.1321268)

Eubacterium eligens group.scatter_plot of EFMR(main)

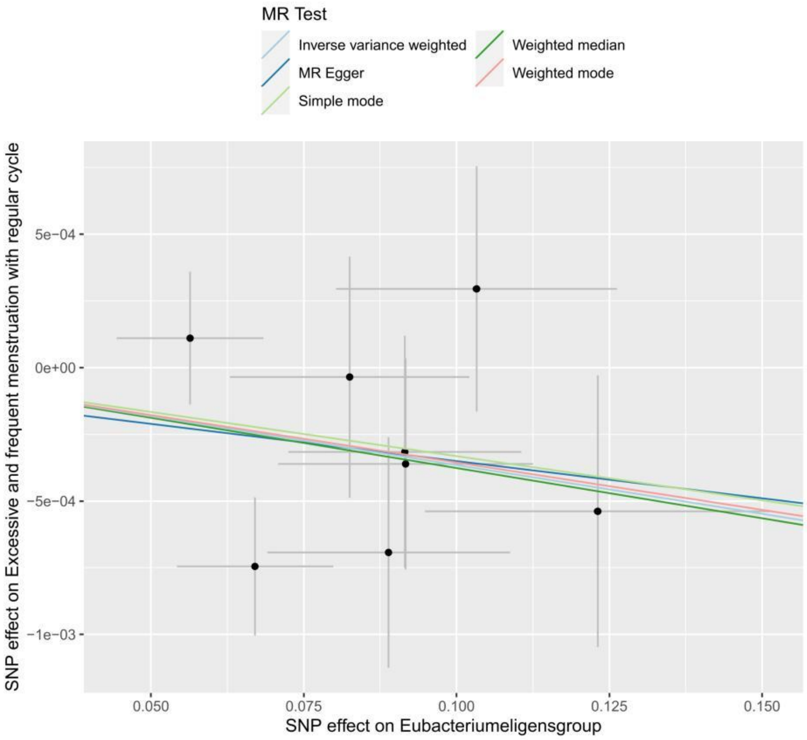

RuminococcaceaeUCG011.scatter_plot of EFMR(main)

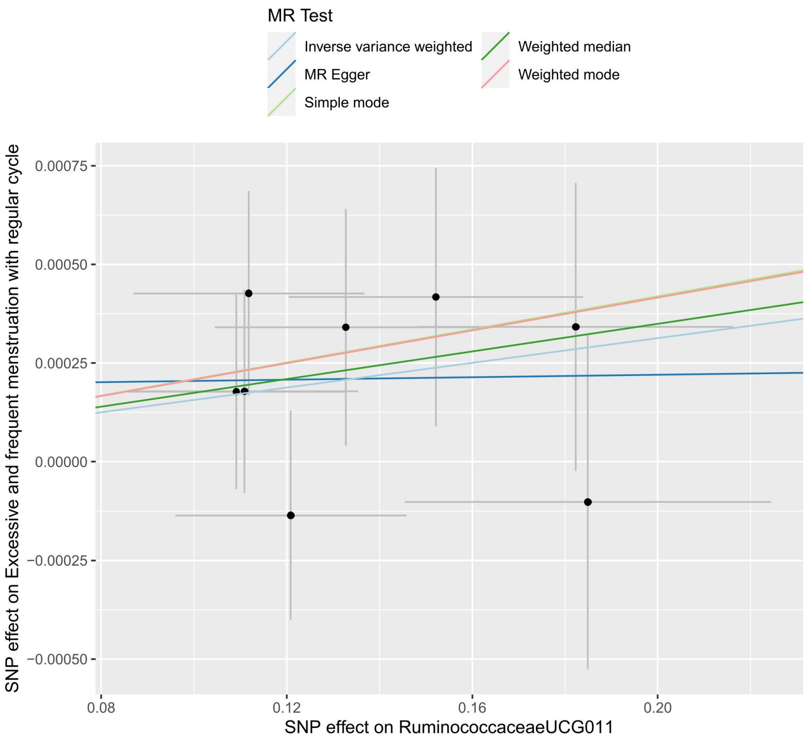

DefluviitaleaceaeUCG011.scatter_plot of EFMR(main)

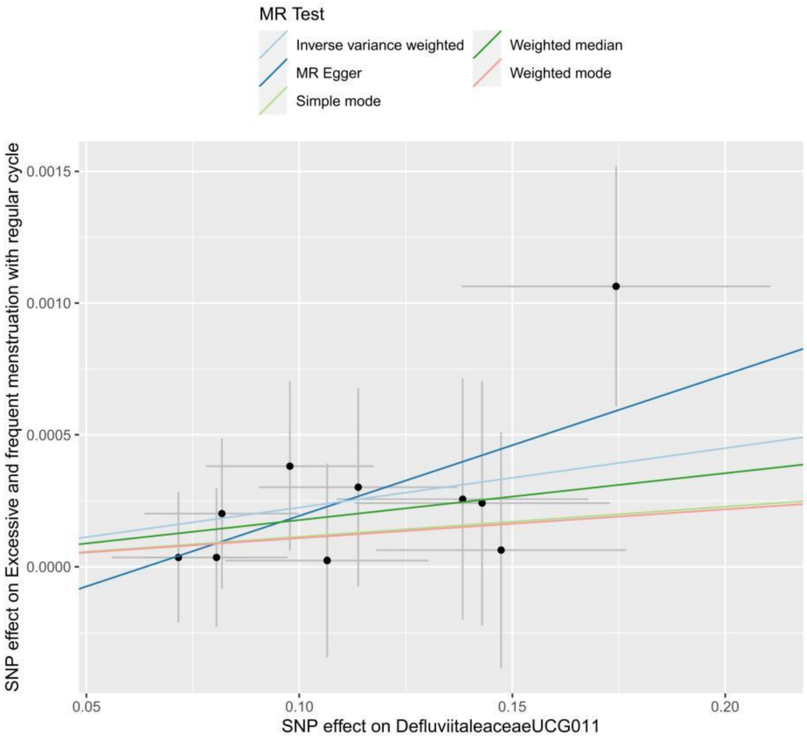

Escherichia.Shigella.scatter_plot of EFMR(main)

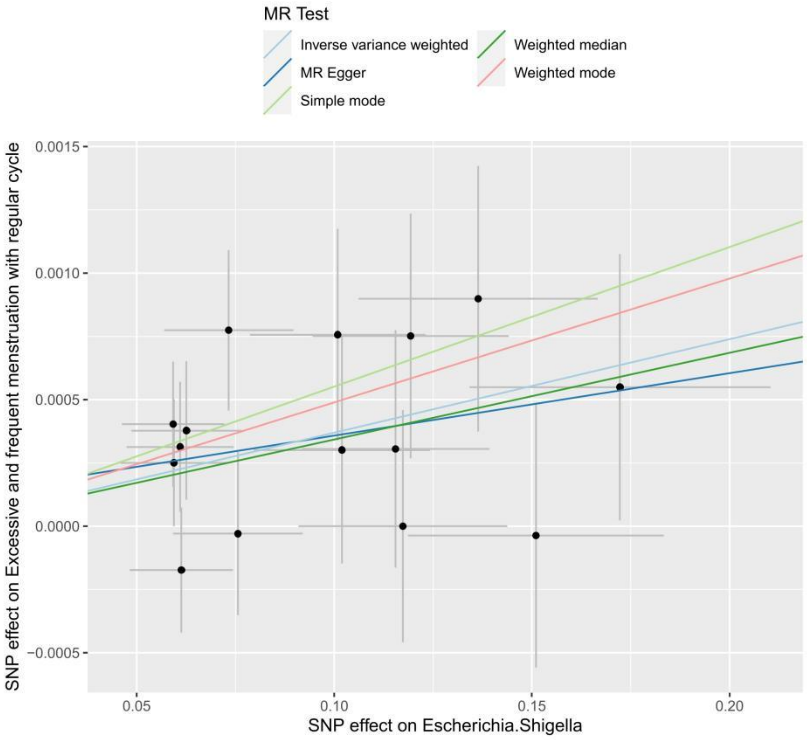

Haemophilus.scatter_plot of EFMR(main)

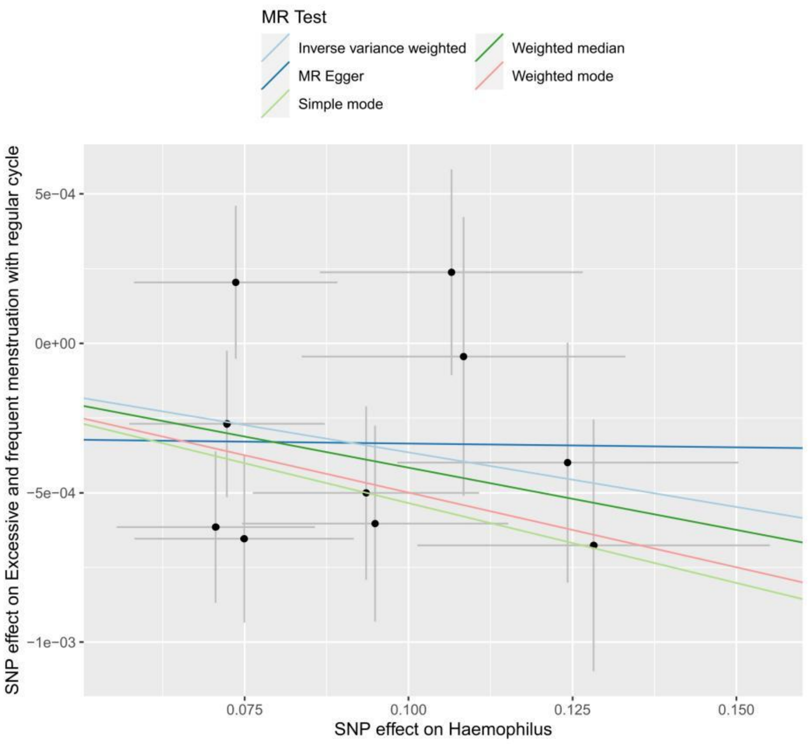

Phascolarcto bacterium.scatter_plot of EFMR(main)

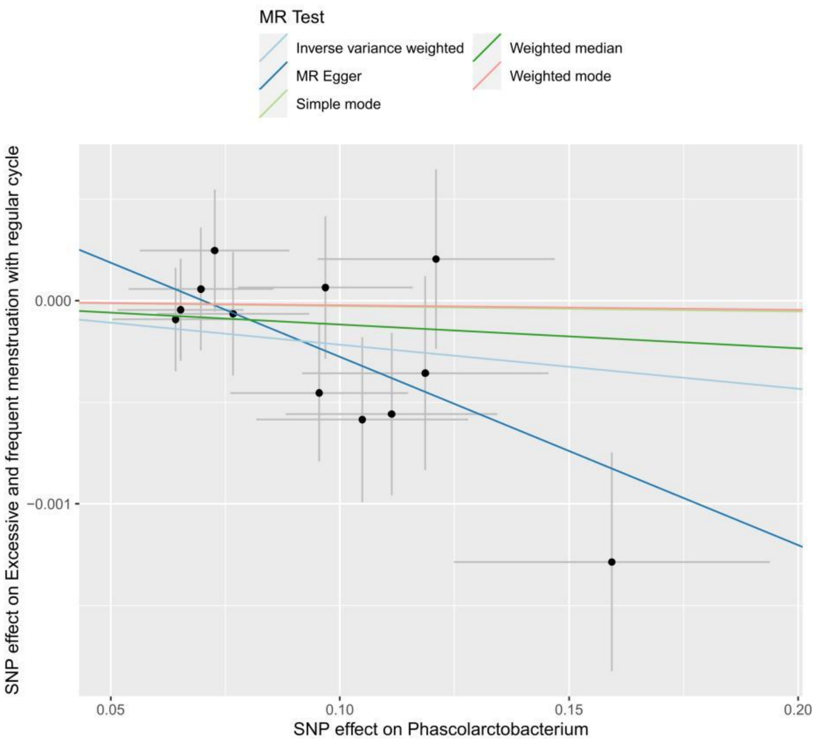

Lachnospira.scatter_plot of EFMR(main)

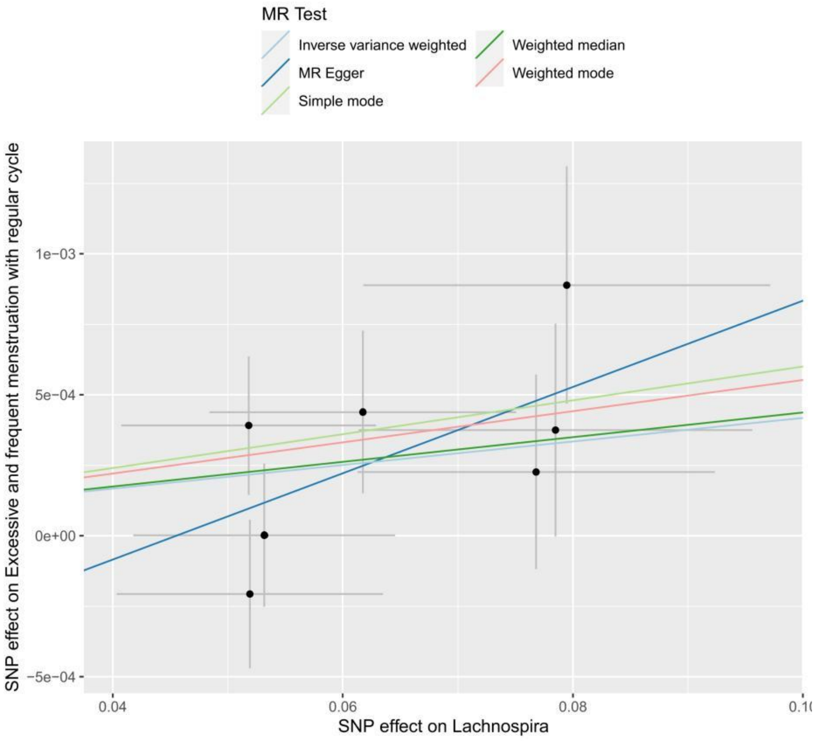

Cateni bacterium.scatter_plot of EFMR(main)

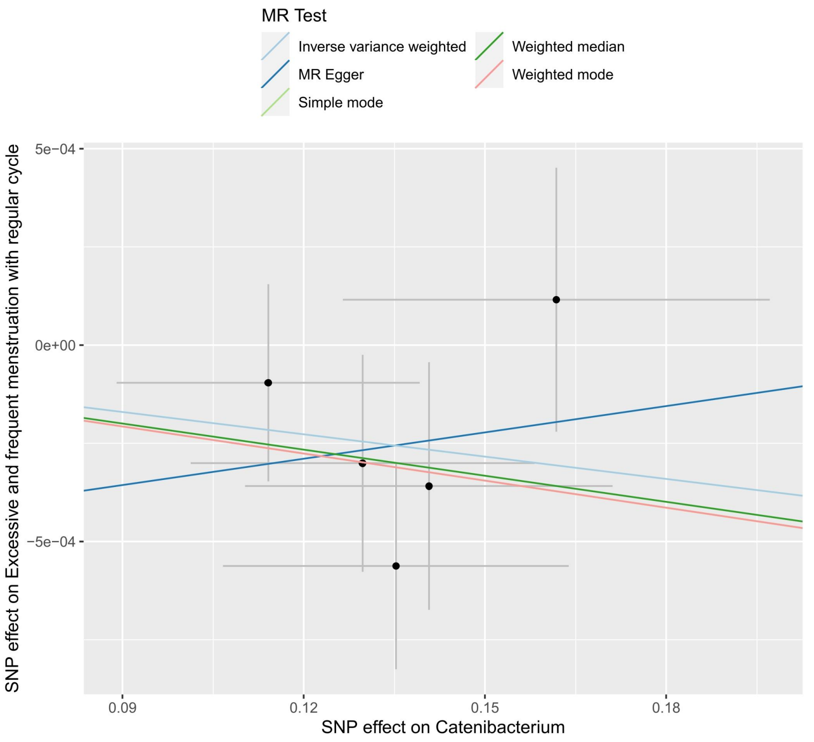

Anaerotruncus.scatter_plot of EFMR(main)

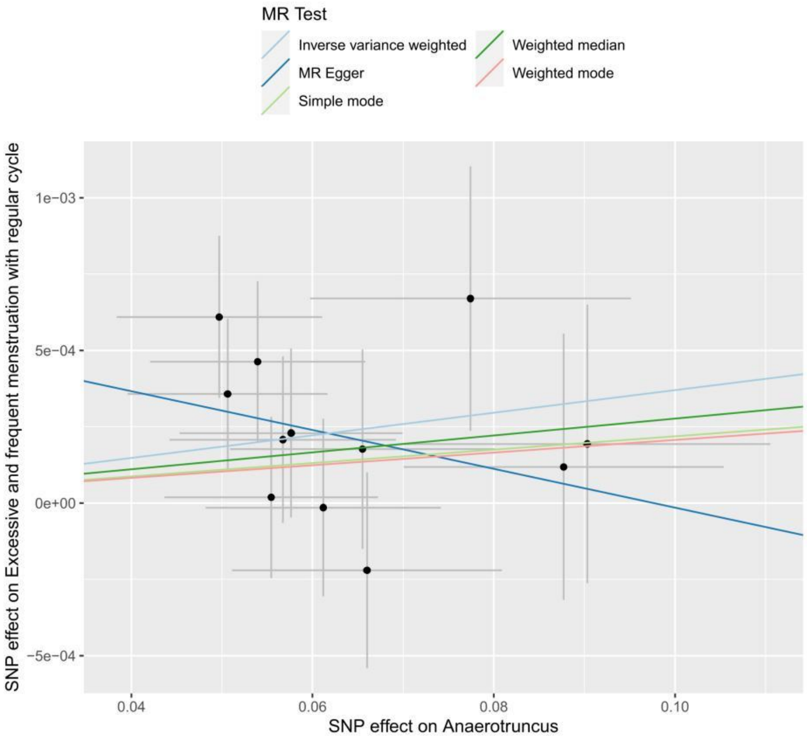

Blautia.scatter_plot of EFMR(main)

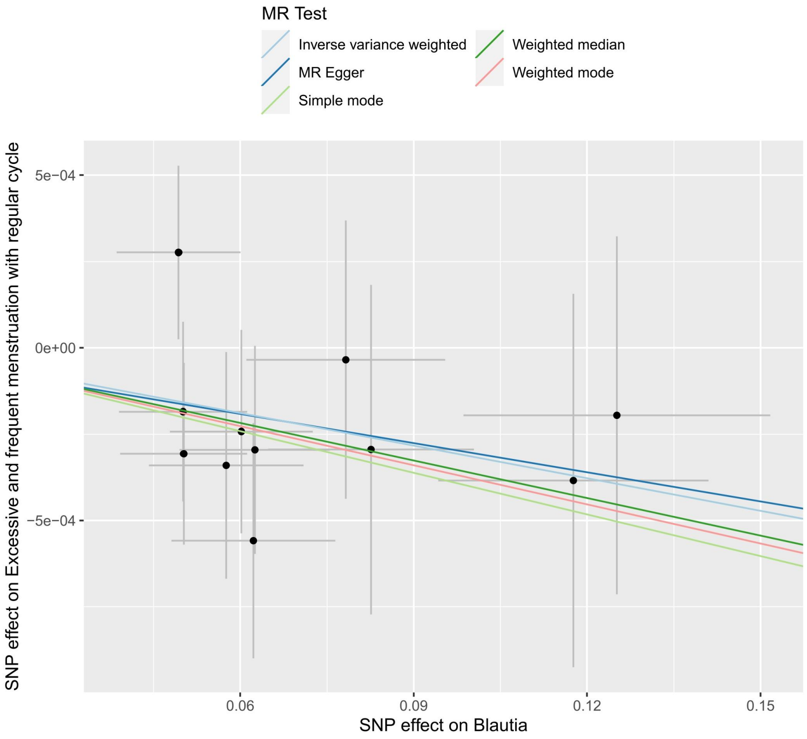

Marvinbryantia.scatter_plot of EFMR(main)

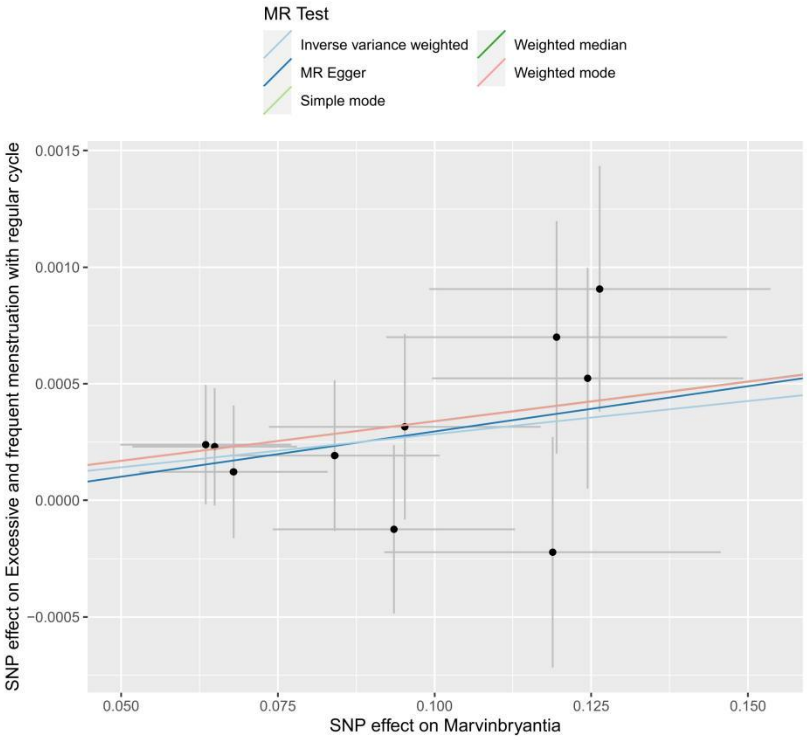

Ruminiclostridium5.scatter_plot of EFMR(secondary)

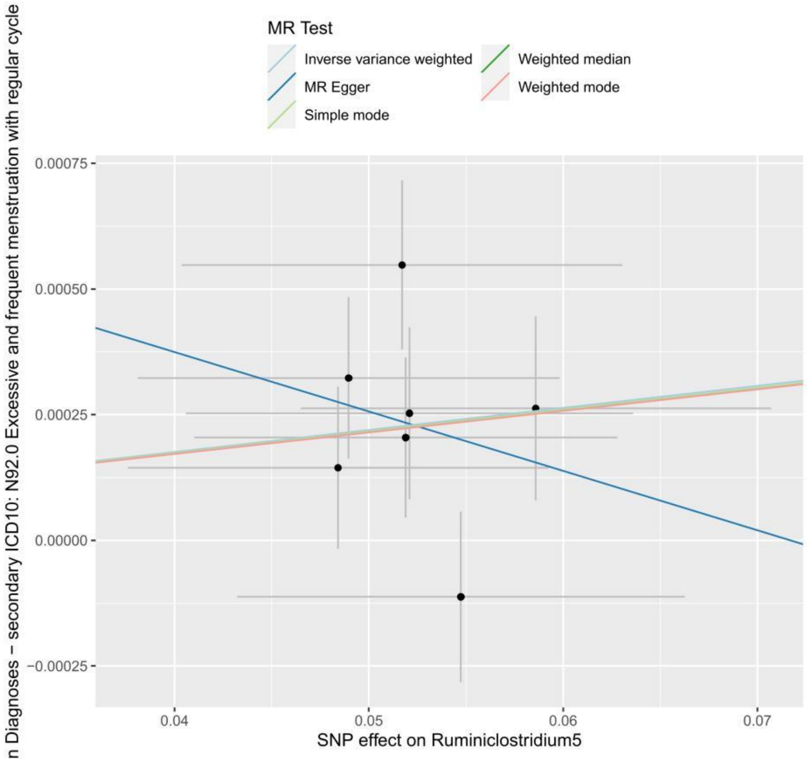

Prevotella9.scatter_plot of EFMR(secondary)

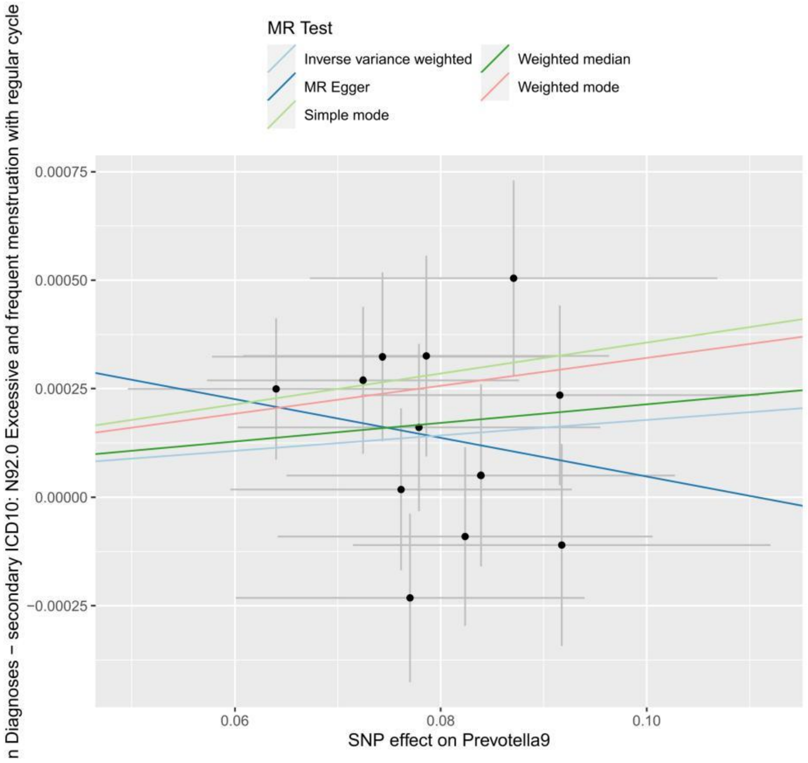

Desulfovibrio.scatter_plot of EFMR(secondary)

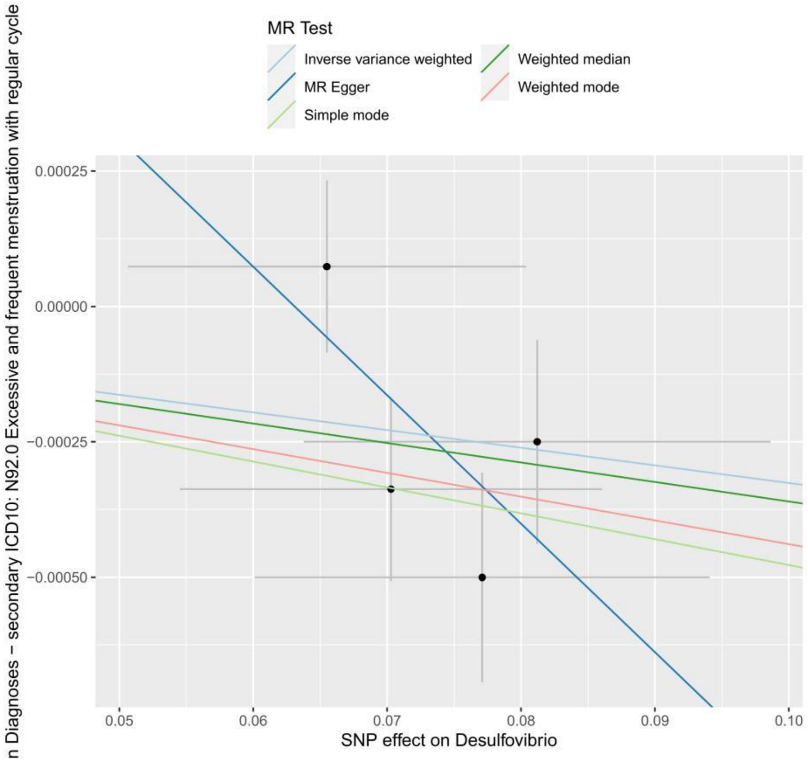

Erysipelatoclostridium.scatter_plot of EFMR(secondary)

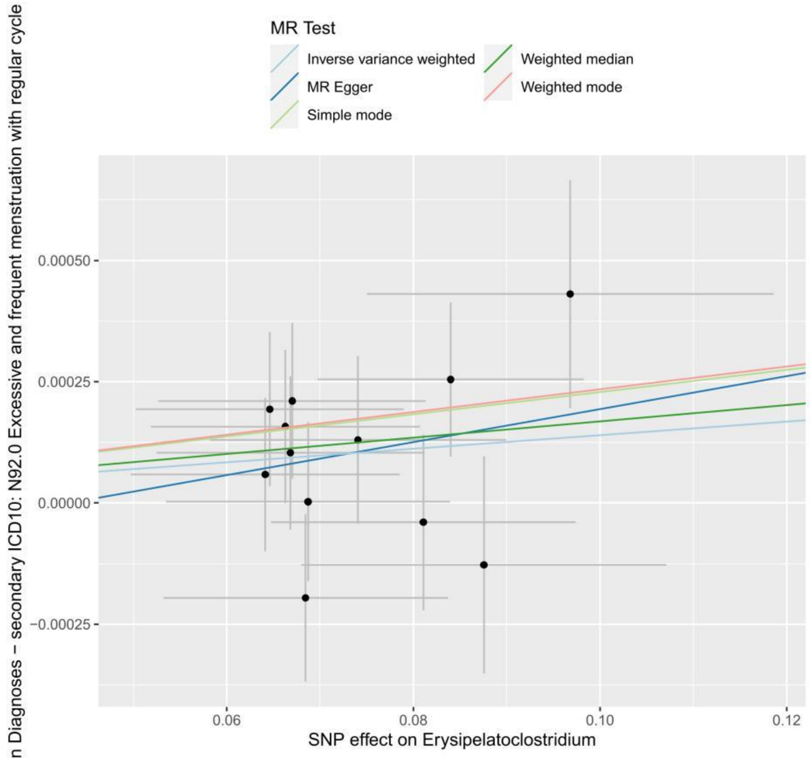

RuminococcaceaeUCG004.scatter_plot of EFMR(secondary)

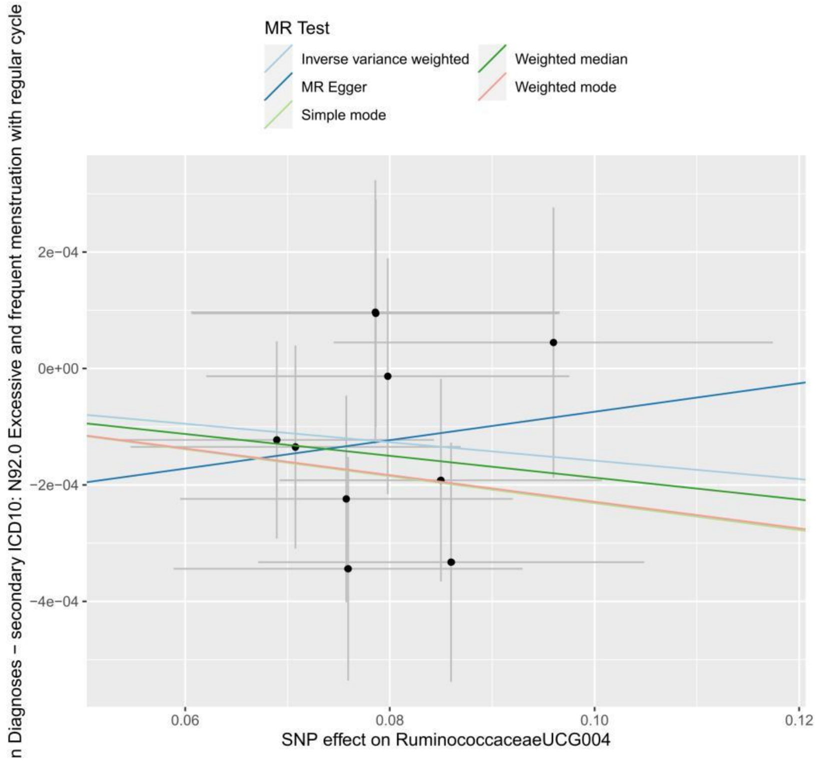

Eubacterium fissicatena group.scatter_plot of EFMR(secondary)

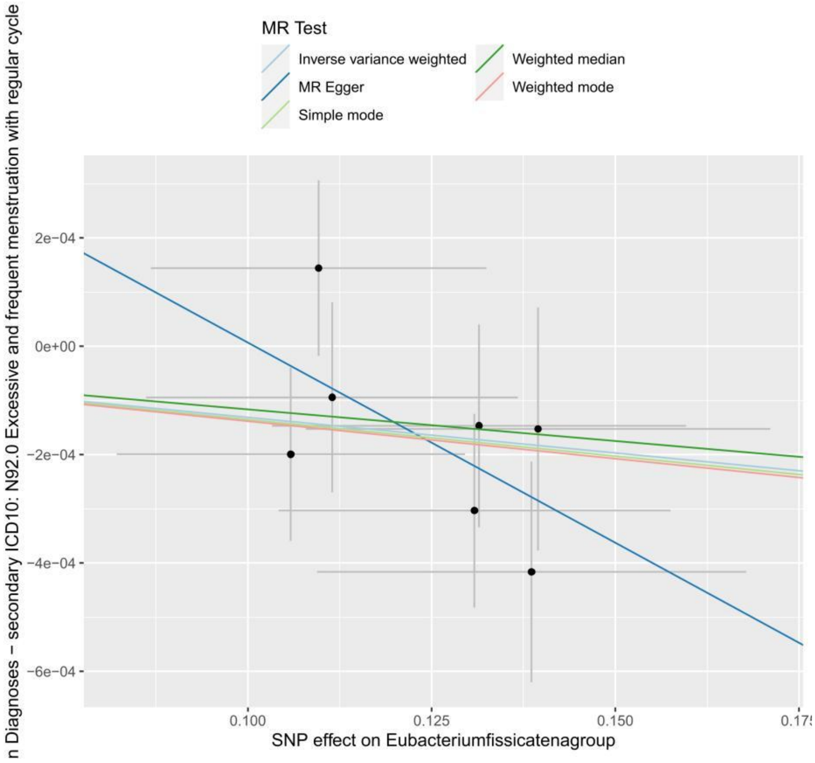

Eubacterium eligens group.scatter_plot of EFIM

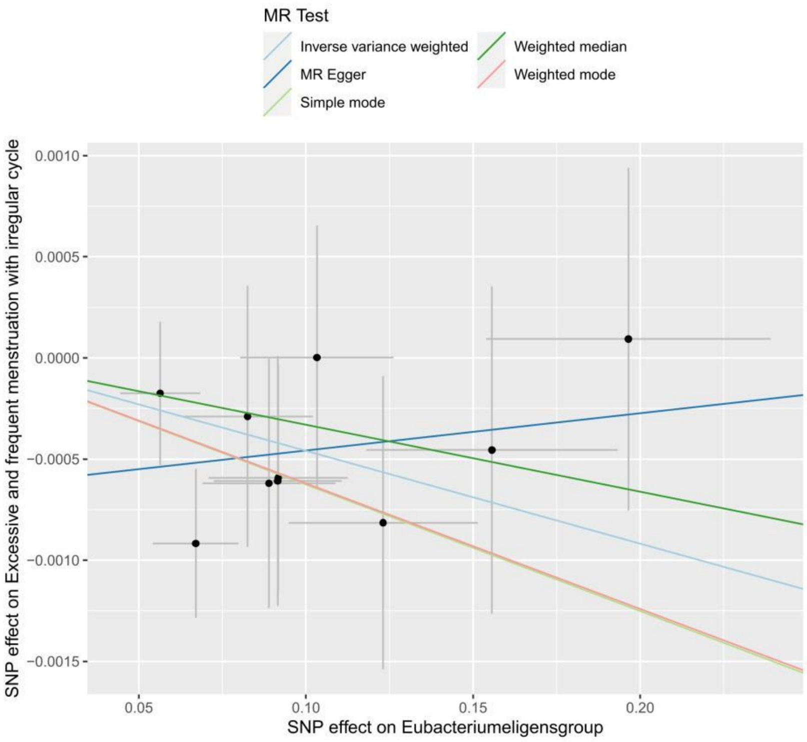

Eubacterium brachy group.scatter_plot of EFIM

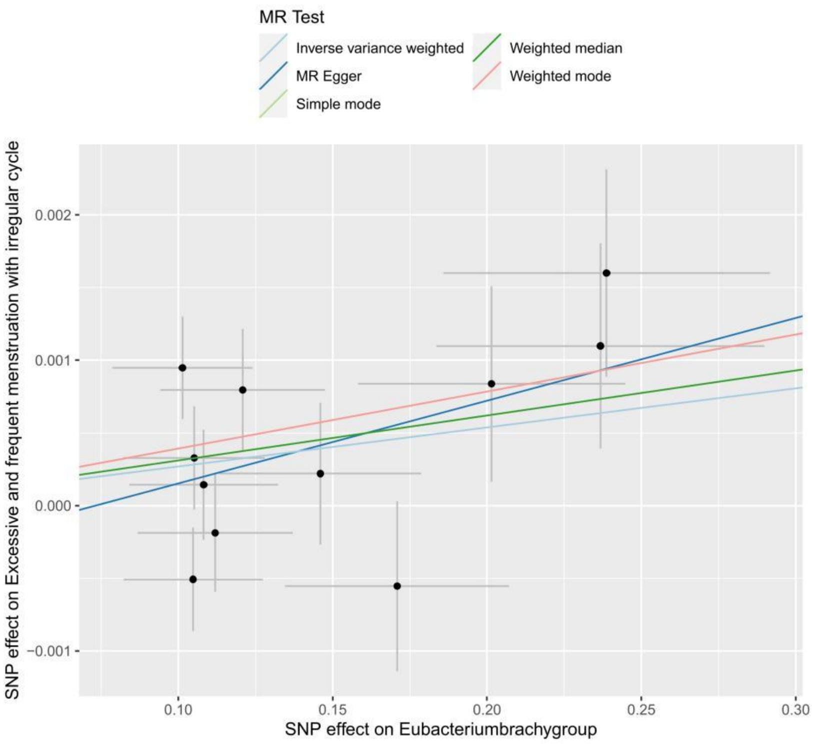

Veillonella.scatter_plot of EFIM

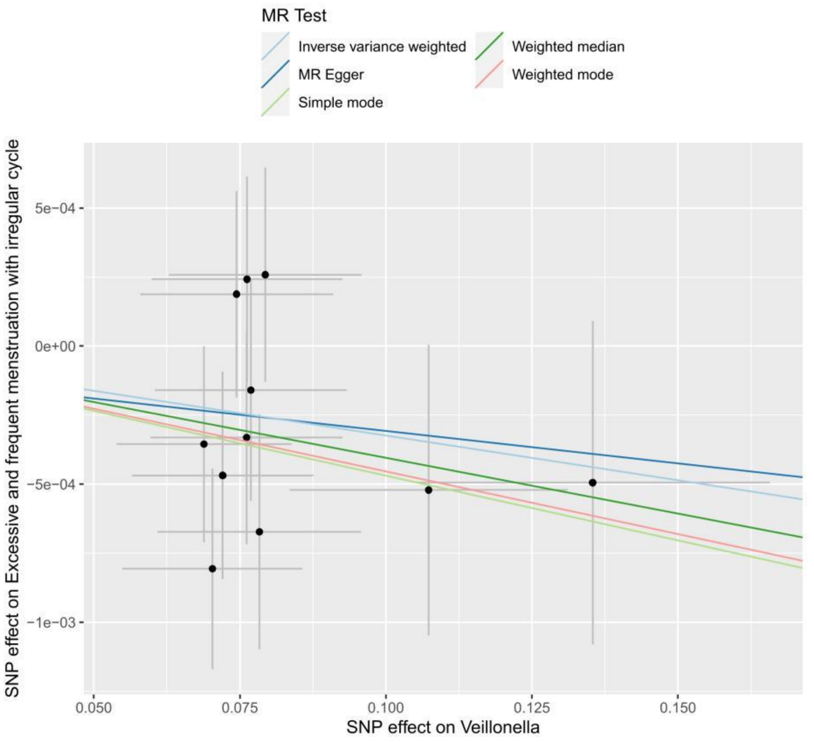

Enterorhabdus.scatter_plot of EFIM

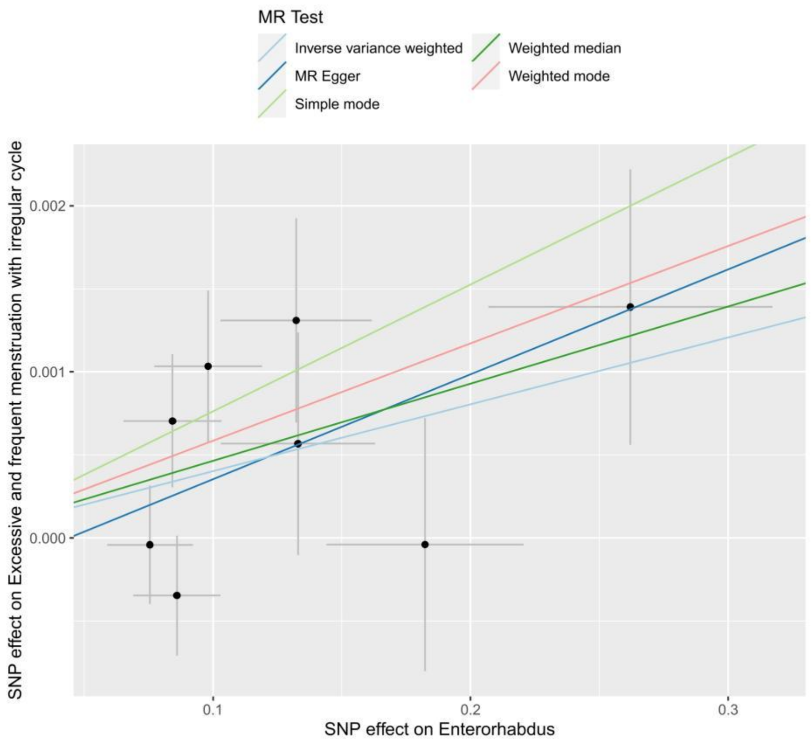

Lactococcus.scatter_plot of EFIM

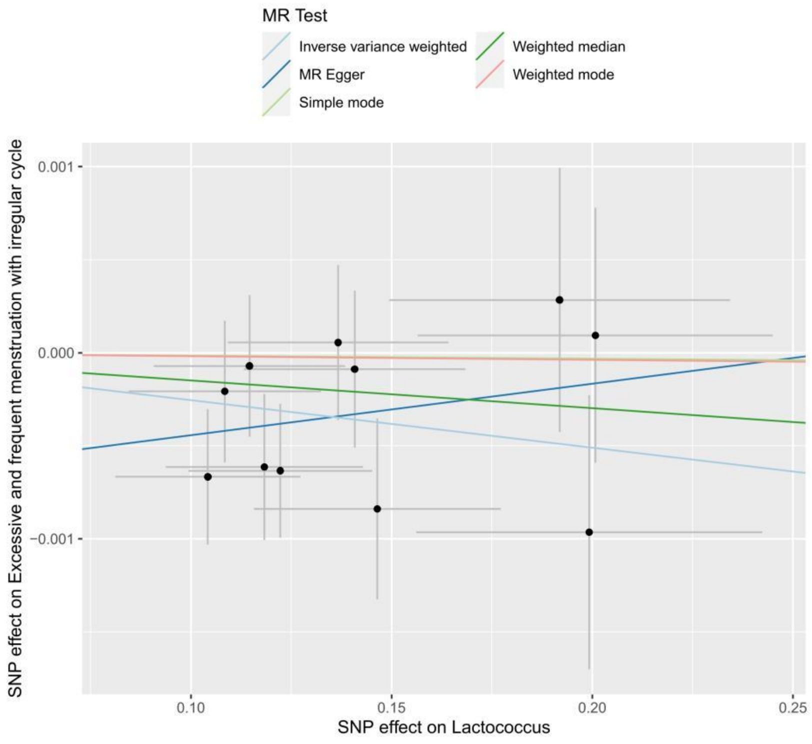

Blautia.scatter_plot of EFIM

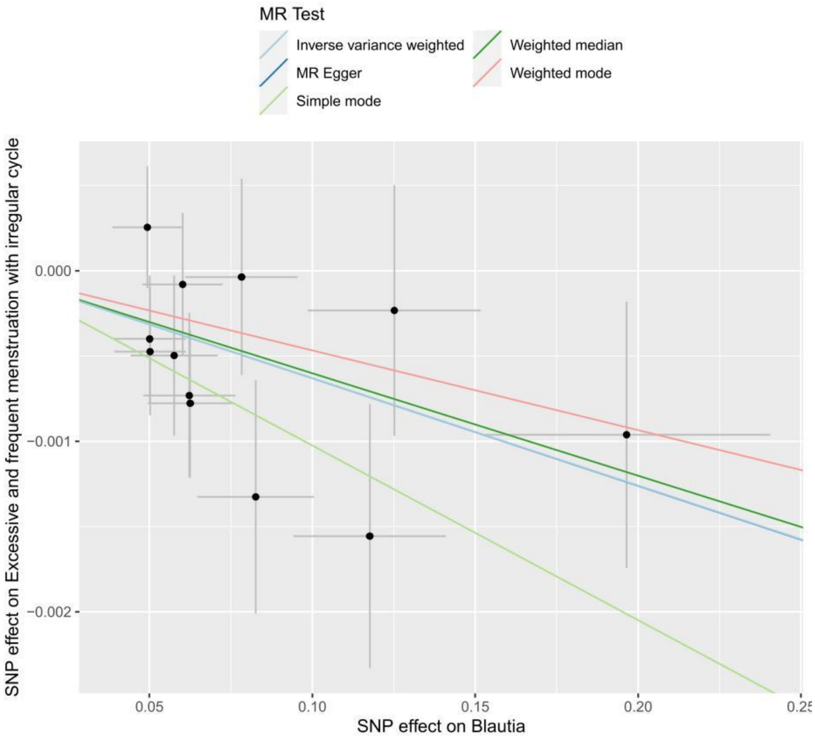

LachnospiraceaeUCG004.scatter_plot of IM(unspecified)

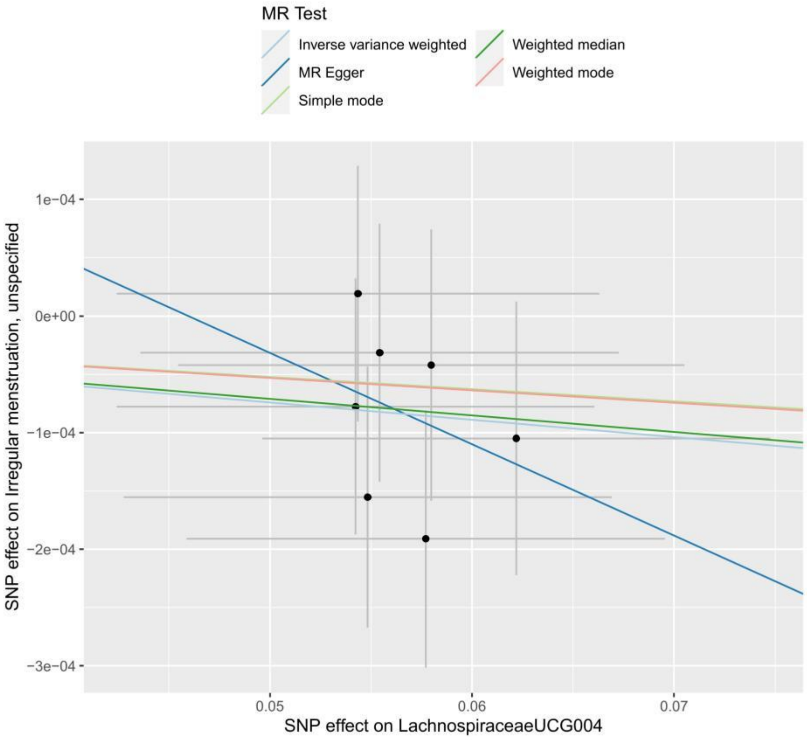

Dialister.scatter_plot of IM(unspecified)

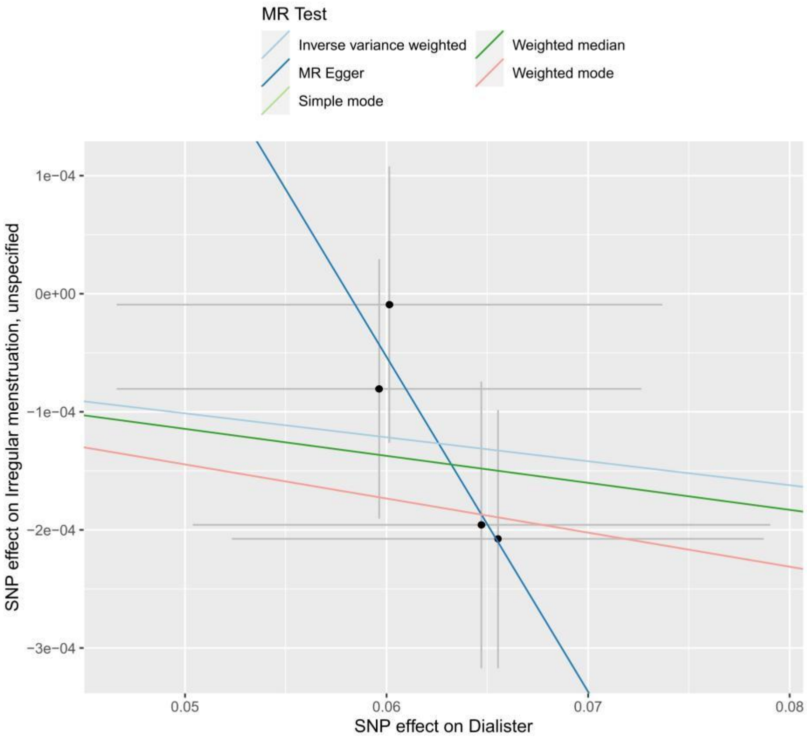

Supplement: Supplementary Figure S4 — Scatter plots of EFMR (main), EFMR (secondary), EFIM, IM(unspecified). [file Data_Sheet_4.PDF]
